# Ad35.CS.01 - RTS,S/AS01 Heterologous Prime Boost Vaccine Efficacy against Sporozoite Challenge in Healthy Malaria-Naïve Adults

**DOI:** 10.1371/journal.pone.0131571

**Published:** 2015-07-06

**Authors:** Christian F. Ockenhouse, Jason Regules, Donna Tosh, Jessica Cowden, April Kathcart, James Cummings, Kristopher Paolino, James Moon, Jack Komisar, Edwin Kamau, Thomas Oliver, Austin Chhoeu, Jitta Murphy, Kirsten Lyke, Matthew Laurens, Ashley Birkett, Cynthia Lee, Rich Weltzin, Ulrike Wille-Reece, Martha Sedegah, Jenny Hendriks, Isabella Versteege, Maria Grazia Pau, Jerold Sadoff, Yannick Vanloubbeeck, Marc Lievens, Dirk Heerwegh, Philippe Moris, Yolanda Guerra Mendoza, Erik Jongert, Joe Cohen, Gerald Voss, W. Ripley Ballou, Johan Vekemans

**Affiliations:** 1 Walter Reed Army Institute of Research, Silver Spring, MD, United States of America; 2 Center for Vaccine Development, University of Maryland School of Medicine, Baltimore, MD, United States of America; 3 PATH-MVI, Washington, DC, United States of America; 4 Naval Medical Research Center, Silver Spring, MD, United States of America; 5 Crucell Holland BV, Leiden, Netherlands; 6 GSK Vaccines, Rixensart, Belgium; Public Health England, UNITED KINGDOM

## Abstract

**Methods:**

In an observer blind, phase 2 trial, 55 adults were randomized to receive one dose of Ad35.CS.01 vaccine followed by two doses of RTS,S/AS01 (ARR-group) or three doses of RTS,S/AS01 (RRR-group) at months 0, 1, 2 followed by controlled human malaria infection.

**Results:**

ARR and RRR vaccine regimens were well tolerated. Efficacy of ARR and RRR groups after controlled human malaria infection was 44% (95% confidence interval 21%-60%) and 52% (25%-70%), respectively. The RRR-group had greater anti-CS specific IgG titers than did the ARR-group. There were higher numbers of CS-specific CD4 T-cells expressing > 2 cytokine/activation markers and more ex vivo IFN-γ enzyme-linked immunospots in the ARR-group than the RRR-group. Protected subjects had higher CS-specific IgG titers than non-protected subjects (geometric mean titer, 120.8 vs 51.8 EU/ml, respectively; *P* = .001).

**Conclusions:**

An increase in vaccine efficacy of ARR-group over RRR-group was not achieved. Future strategies to improve upon RTS,S-induced protection may need to utilize alternative highly immunogenic prime-boost regimens and/or additional target antigens.

**Trial Registration:**

ClinicalTrials.gov NCT01366534

## Introduction

The renewed emphasis on malaria control, elimination, and eventual eradication has stimulated significant investment in a variety of tools that prevent infection, decrease morbidity and mortality from the disease, and disrupt transmission of the parasite between the host and the mosquito vector. The incidence of malaria in much of Africa remains extremely high despite observed declines in morbidity across a range of settings where large scale malaria control programs have been implemented [1, 2, 3]. The development of a malaria vaccine has been identified as a key component of future integrated malaria control programs and an important step towards sustainable elimination of malaria [4, 5].

The RTS,S/AS01 candidate malaria vaccine consists of the recombinant protein RTS,S, which is comprised of part of the central repeat and C-terminal flanking regions of the CS protein and hepatitis B surface antigen (HBsAg), with the proprietary adjuvant AS01 [6–9]. The Ad35.CS.01 vaccine is comprised of the homologous 3D7 full-length CS minus the GPI anchor domain. The target malaria CS antigen is expressed on the surface of the infective stage sporozoites, and intrahepatic exoerythrocytic parasites. Immunization with RTS,S/AS01 consistently provides complete or partial protection in a significant proportion of malaria naïve volunteers undergoing controlled human malaria infections (CHMI) [10–14]. When evaluated in African adults and children exposed to malaria-infected mosquitoes, RTS,S/AS01 has consistently demonstrated partial vaccine efficacy against clinical uncomplicated malaria and severe disease [15–23].

Although implementation of the RTS,S/AS01 vaccine in immunization programs may result in a substantial reduction of malaria burden in children, increasing the magnitude and breadth of anti-CS immune responses could lead to improvements in vaccine efficacy levels for better malaria control, and may be a tool in future malaria elimination efforts. Potential ways to improve RTS,S include augmenting its antibody response by increasing the CD4 T cell responses elicited after immunization. CS-specific CD4 cellular responses assessed using ELISpot and/or intracellular cytokine staining (ICS) assays have been observed following RTS,S immunization in adults and children. In CHMI trials in adults and in one Phase 2B trial in children aged 5–17 months at first vaccination, anti-CS CD4 responses have been found to be associated with protection [13, 24–29].

Heterologous prime-boost strategies have been shown to increase the magnitude and/or breadth of the vaccine-specific immune responses [30, 31]. Sequential immunization with a vaccine candidate that elicits strong CS-specific T cell responses and RTS,S/AS01, a potent inducer of CS-specific antibody as well as CD4 T cell responses, could represent an ideal strategy towards increased vaccine efficacy. The full length CS-expressing replication-deficient recombinant human adenovirus 35 (Ad35.CS.01, Crucell NV, Leiden, The Netherlands) was shown in pre-clinical and clinical studies in mice, monkeys, and man to elicit both anti-CS antibody and T-cell responses [32–37]. The rationale for the current human clinical trial was based on a pre-clinical immunization regimen in non-human primates in which a single priming immunization of Ad35.CS.01 followed by two doses of RTS,S/AS01 showed greater numbers of CS-specific IFN-gaama ELISpot (16-fold increase) and CS-specific CD4^+^ T-cells expressing at least two cytokines (IL-2, IFN-γ, TNF-α) compared to the standard regimen of three doses of RTS,S/AS01 alone [35].

## Subjects, Material and Methods

### Study design and subjects

**Fig 1:**
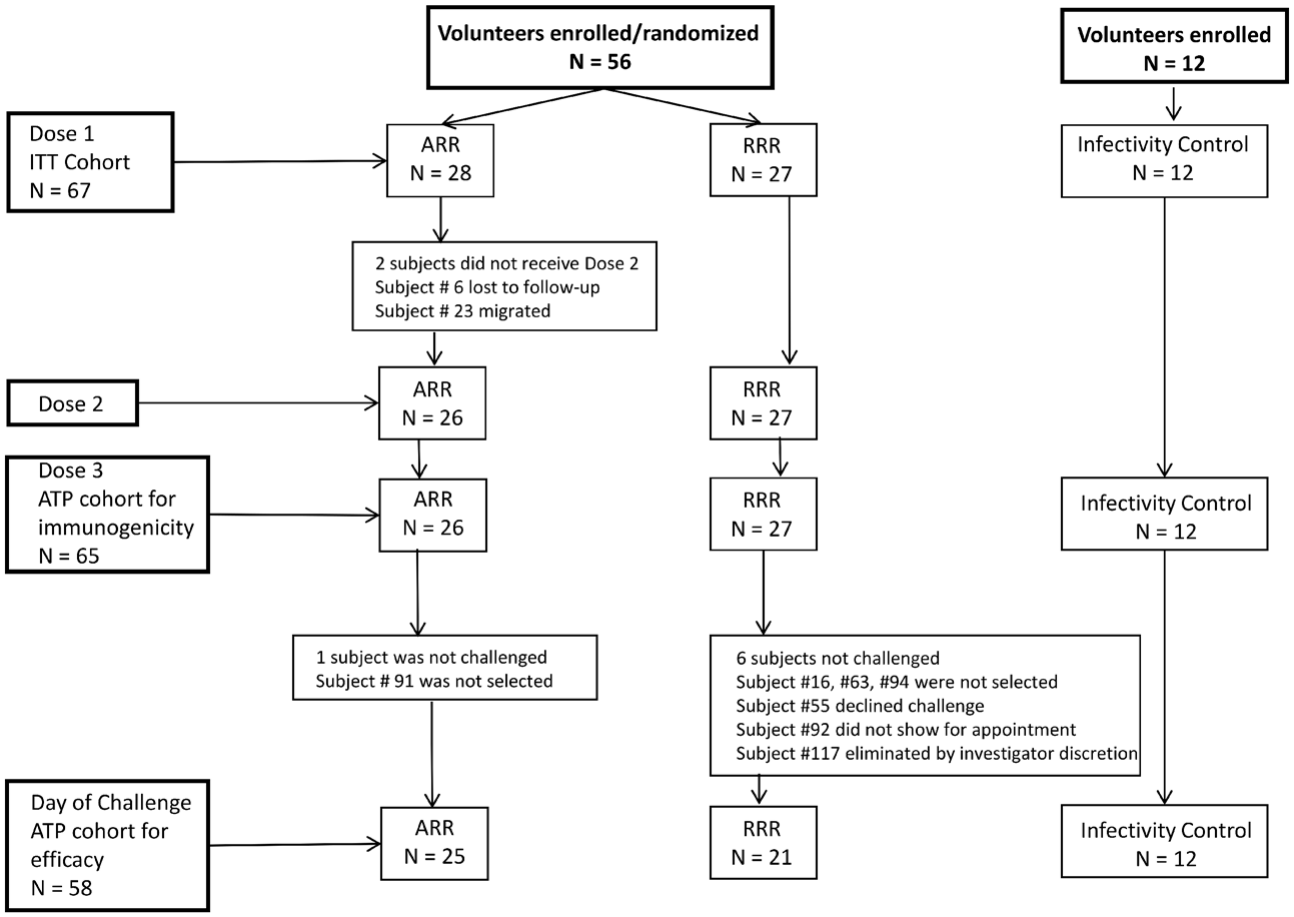
Flow diagram. The number of subjects completing each immunization and controlled human malaria infection. ARR = first dose with Ad35.CS, second and third doses with RTS,S/AS01_B_. RRR = three doses of RTS,S/AS01_B_

This was an observer-blind, randomized Phase 2a study in healthy malaria-naïve adults designed to evaluate safety, reactogenicity, immunogenicity, and efficacy of Ad35.CS.01 followed by two doses of RTS,S/AS01 (ARR group) compared to three doses of RTS,S/AS01 (RRR group) (www.clinicaltrials.gov NCT01366534; Fig 1), conducted at the Walter Reed Army Institute of Research (WRAIR) between August 2011 and July 2012. Vaccination and challenge were to be performed in three sequential cohorts (Cohorts A, B and C). Each cohort to be vaccinated was to include approximately 56 subjects of which a random selection of subjects eligible for challenge was done to ensure that a maximum of 46 vaccinated individuals progressed to the challenge phase, given logistical constraints. Eligibility criteria are presented in S1 Text available with this manuscript. The results in this paper refer only to those in Cohort A as pre-defined criteria to progress to cohort B and C were not met.

The protocol was approved by the WRAIR Institutional Review Board and PATH-Malaria Vaccine Initiative’s Western IRB. The trial was undertaken in accordance with the provisions of the International Conference on Harmonization and Good Clinical Practice guidelines. Written informed consent was obtained from each subject before study procedures were initiated.

### Study vaccines and vaccine administration

The RTS,S vaccine antigen consists of 19 NANP amino acid repeat units followed by the complete C-terminal domain minus the GPI anchor of the CS antigen, fused to the Hepatitis B virus S protein. The S protein corresponds to the surface antigen of Hepatitis B virus (HBsAg). The adult dose of RTS,S (50 μg) used in this study was formulated with a liposome Adjuvant System AS01 [6]. Ad35.CS.01 (5 x 10^10^ vp per dose) is a human adenovirus 35 vaccine candidate expressing recombinant full-length CS protein [36]. The Ad35 vaccine vector produced in the E1-complementing PER.C6 cell line has E1 and E3 regions deleted and is replication-incompetent. Ad35.CS.01, incorporates the full length CS gene. The N-terminus region has a consensus sequence designed to cover various circulating polymorphisms which is lacking in RTS,S. The C-terminus sequence is identical to that in the RTS,S (3D7-derived) vaccine. There are 27 NANP repeats, a cluster of 3 NANPNVDP repeats, and 1 separate NVDP repeat into the CS protein. Both RTS,S/AS01 and Ad35.CS.01 vaccines were administered intramuscularly at monthly intervals in the deltoid muscle of the non-dominant arm.

### Efficacy assessments

Controlled human malaria infection (CHMI) through the bite of five P. falciparum (3D7 strain) infected mosquitoes of vaccinated subjects and unvaccinated infectivity control volunteers occurred 3 weeks after the last vaccination visit on day of challenge (DoC, Day 77). Parasitemia was detected by review of daily blood films from 5 to 18 days post challenge, and every two days up to 28 days. Chloroquine phosphate was used to treat those volunteers who became infected with malaria.

### Safety assessments

Safety monitoring was performed by a Safety Monitoring Committee. Local injection site and general adverse events (AEs) were solicited over 7 days post each vaccination (Days 0–6). AEs were graded as mild, moderate or severe (Grades 1–3 respectively). All other AEs (unsolicited) were recorded over a 30 day period after each vaccination. Serious adverse events (SAEs) were captured throughout the study. See S1 Text for additional details on safety assessment including holding rules.

### Immunomonitoring

Antibody levels against CS repeat region were measured by standard ELISA methodology using plate-adsorbed R32LR antigen [NVDP(NANP)_15_]_2_LR and expressed in ELISA Units/milliliter (EU/mL) [38]. Methods measuring antibodies levels against Ad35-specific neutralizing antibody titers and methods describing the cell-mediated immune responses as measured by ELISpot and Intracellular Cytokine Staining (ICS) are described in S1 Text.

### Statistical analysis

All safety, immunomonitoring, and efficacy analyses were conducted according to a pre-defined plan and expanded details including on immunological analyses are available in S1 Text Analysis for efficacy was performed on the According to Protocol (ATP) cohort, and was assessed by comparison of parasitemia incidence after sporozoite challenge and secondly by time to onset of parasitemia. Vaccine efficacy (VE) was defined as 100*(1-Relative Risk). Fisher’s Exact test was used for the comparison of malaria incidence up until 28 days after challenge between the two vaccinated groups. Kaplan-Meier analysis was performed on time to onset of parasitemia, with testing between the two treatment groups using the log-rank statistic. Futility analyses were conducted on efficacy data collected up to 28 days post-challenge for the first 46 subjects vaccinated and challenged in Cohort A. The attack rate is defined as the: number of subjects with documented parasitemia after CHMI / total number of subjects per group challenged by five infectious mosquito bites in CHMI. Vaccination of Cohorts B and C could only proceed if the calculated point estimate of increase of VE in the ARR group over the RRR group was over 0%, providing a 4% risk of stopping the trial if the true increase in VE is 50%, assuming an attack rate of 50% in the RTS,S/AS01 group. The increase in VE of the ARR group over the RRR group was defined as 100*(1-AR Ad35.CS.01 / AR RTS,S) where AR is the attack rate.

## Results

### Subject cohort

A total of 67 subjects were enrolled in the study of which 55 subjects (28 ARR, 27 RRR) were randomly assigned to a study group and received at least one vaccine dose, and 12 subjects participated as infectivity controls. Fig 1 summarizes subject participation during the course of the study. In total, 58 subjects underwent sporozoite challenge (ATP efficacy cohort: 25 ARR group, 21 RRR group, 12 infectivity controls).

At the time of first vaccine administration the mean age of subjects was 30 years in both the ARR, RRR, and infectivity control groups. More females were enrolled in the ARR group (57%) and more males were enrolled in the RRR group (63%); gender was balanced in the infectivity control group.

### Safety outcomes

No SAE was reported in any subject and no subject was withdrawn due to an AE. Immunizations of subjects in both ARR and RRR groups were well tolerated and no safety halting criteria were met. No clinically concerning imbalances in solicited or unsolicited adverse events were observed between groups, overall or when considering dose 1 only (S1 Table, S1 Fig). There was no indication that Ad35.CS.01 priming influenced the occurrence of local or general symptoms following subsequent RTS,S/AS01 doses. The solicited local symptom with highest incidence post-vaccination per dose in both the ARR and RRR groups was pain (86.6% and 74.1%, respectively). Grade 3 pain (<2.5% of doses) and redness (1.2% of doses) were infrequent. The solicited general symptoms with highest incidence post-vaccination overall per dose in both the ARR and RRR groups were fatigue (31.7% and 39.5%, respectively) and headache (43.9% and 34.6%, respectively). The incidence of fever overall per dose was 7.3% and 12.3% in the ARR and RRR groups respectively. Laboratory abnormalities were infrequent, not clinically significant, and not related to vaccination.

### Efficacy outcomes

**Fig 2:**
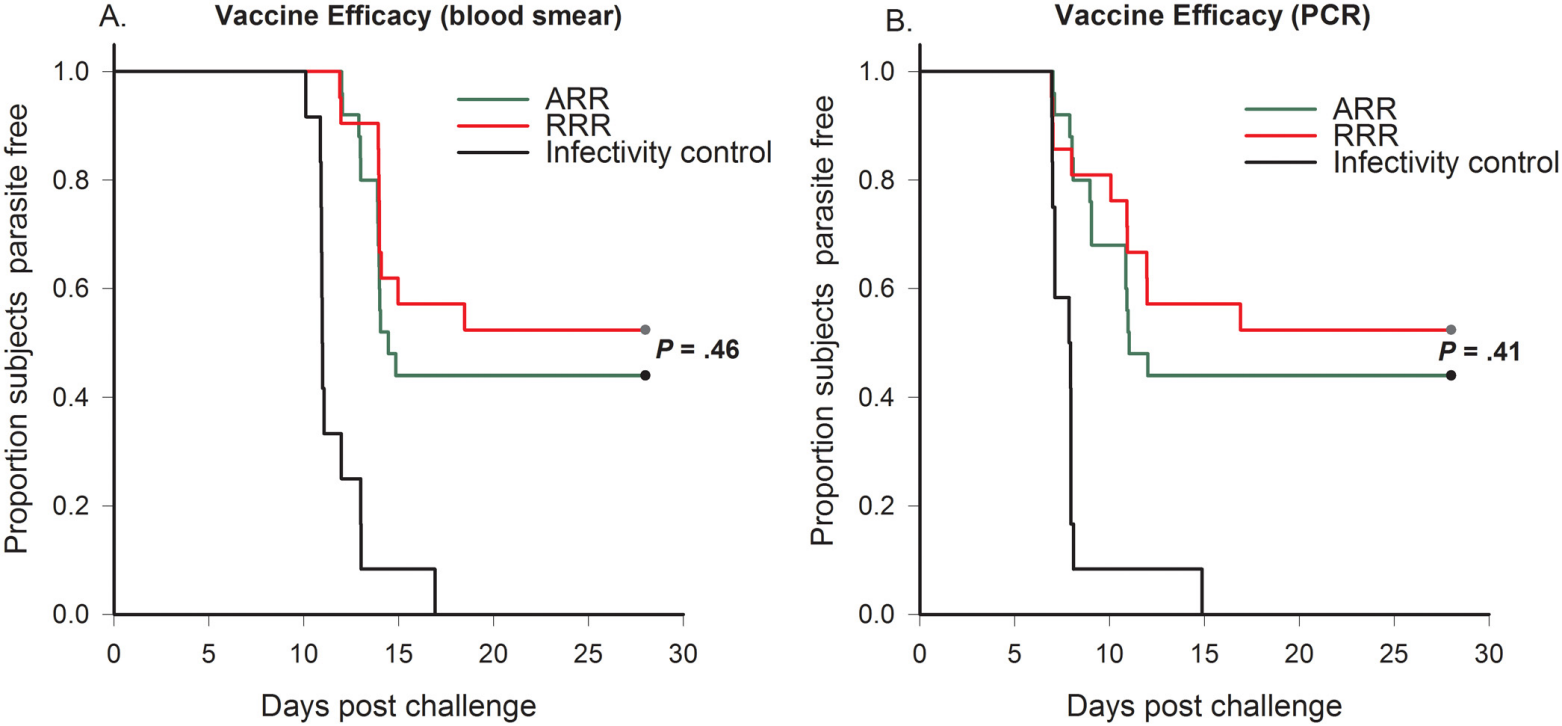
Kaplan-Meier curves for time to parasitemia by blood smear (A) and PCR (B) for ARR (green), RRR (red), infectivity control (black) subjects (ATP efficacy). Log-rank analysis showed statistically significant differences between ARR and control subjects (P < .0001) and RRR and control subjects (P < .0001).

Patent parasitemia developed in 14 of 25 (56.0%) ARR vaccinees, 10 of 21 (47.6%) RRR vaccinees, and 12 of 12 (100%) infectivity controls (Table 1). VE in the ARR group was 44% (95% CI: 21–60; p = .007) and in the RRR group 52% (95% CI: 25–70; p = .002), representing a decrease in VE of -18% (95% CI: -108, -33; p = .77) in ARR. The time to first parasitemia is shown in a plot of the Kaplan Meier survival estimate in Fig 2, indicating no significant difference between ARR and RRR groups by blood smear (*P* = .46) and by PCR (*P* = .41) in contrast to highly significant differences between ARR and RRR compared to infectivity controls (*P* < .0001). The futility criteria for efficacy were met, and it was determined that further immunization of ARR and RRR groups in cohorts B and C were not indicated.

**Table 1:** Vaccine efficacy against positive blood slide parasitemia after sporozoite challenge (ATP cohort for vaccine efficacy).

Group	# Subjects	# subjects positive parasitemia	% subjects with parasitemia (95% CI)	VE (95% CI)	*P*-value
Control	12	12	100.0 (73.5–100.0)	-	-
ARR	25	14	56.0 (34.9–75.6)	44.0 (20.7–60.4)	.007
RRR	21	10	47.6 (25.7–70.2)	52.4 (25.4–69.6)	.002

Incremental VE of ARR over RRR, -17.6 (-107.4–33.3); P = .768

ARR; first dose with Ad35.CS, second and third doses with RTS,S/AS01B

RRR; three doses of RTS,S/AS01B

VE (%), Vaccine Efficacy

CI, 95% Lower and Upper Confidence Intervals

P-value; Two-sided Fisher Exact Test

Vaccine efficacy; 100*(1 –attack rate ARR/ attack rate RRR)

### Immunogenicity outcomes

#### Anti-CS repeat antibody and cell-mediated immune responses by group

In the ARR group, at one month post Dose 1, 69.2% of subjects were sero-positive for anti-CS amino acid repeat (NANP) antibodies while 100% of subjects in the RRR group sero-converted after a single dose of RTS,S/AS01. Anti-CS repeat geometric mean titer (GMT) antibody levels were significantly higher in the RRR group compared to the ARR group at all post vaccination time points except at day 236 (Table 2). Priming with Ad35.CS.01 in the ARR group did not elicit higher anti-CS protein titers after two RTS,S/AS01 booster doses compared to titers in the RRR group after the second immunization (*P* = .191). There were no Ad35 seropositive subjects in the ARR group at screening. It is therefore not possible to assess whether pre-vaccination anti-Ad35 immunity may affect Ad35.CS.01 immunogenicity. Ad35.CS.01 vaccination induced detectable anti-Ad35 antibodies in some subjects in the ARR group. At 28 days post Dose 1, the anti-Ad35 seropositivity rate in the ARR group was 30.8%.

**Table 2:** Seropositivity rates and Geometric Mean Titer (GMT) for anti-CS antibodies overall and by protection status (ATP cohort for immunogenicity).

Days	Group	Seropositivity rates (%)	GMT EU/ml (95% CI)	P value NP vs P	P value ARR vs RRR
D0 (Screening)	ARR	7.7	0.3 (0.2–0.3)		.603
	ARR NP		0.3 (0.2–0.3)	.856	
	ARR P		0.3 (0.2–0.3)		
	RRR	3.7	0.3 (0.2–0.3)		
	RRR NP		0.3 (0.3–0.3)	.354	
	RRR P		0.3 (0.2–0.3)		
D28 (Post 1)	ARR	69.2	0.9 (0.6–1.4)		< .0001
	ARR NP		0.8 (0.4–1.4)	.797	
	ARR P		0.9 (0.4–2.1)		
	RRR	100	14.7 (8.7–24.9)		
	RRR NP		9.3 (3.4–25.3)	.071	
	RRR P		24.7 (13.3–46)		
D56 (Post 2)	ARR	100	14.3 (9.4–21.6)		< .0001
	ARR NP		12.4 (6.1–25.4)	.632	
	ARR P		15.2 (9.6–24)		
	RRR	100	79.2 (51.0–122.9)		
	RRR NP		60.5 (30.4–120.4)	.067	
	RRR P		129.9 (73.8–228.9)		
D77 (Post 3, DoC)	ARR	100	55.5 (40.2–76.6)		.005
	ARR NP		42.6 (28.9–62.9)	.055	
	ARR P		77.6 (45.1–133.4)		
	RRR	100	115.9 (77.0–174.5)		
	RRR NP		68.1 (36.6–126.9)	.010	
	RRR P		188.2 (113.5–312)		
D236 (Post 3)	ARR	100	21.4 (15.3–30.1)		.223
	ARR NP		16.1 (10.4–24.9)	.047	
	ARR P		31.6 (17.8–56)		
	RRR	100	29.2 (19.7–43.4)		
	RRR NP		18.2 (10.3–32.3)	.019	
	RRR P		48.7 (26–91.2)		

ARR = first dose with Ad35.CS, and the second and third doses with RTS,S/AS01_B_

RRR = three doses with RTS,S/AS01_B_

NP = non-protected

P = protected

P values calculated using log-transformed antibody levels in Students t-test

**Fig 3:**
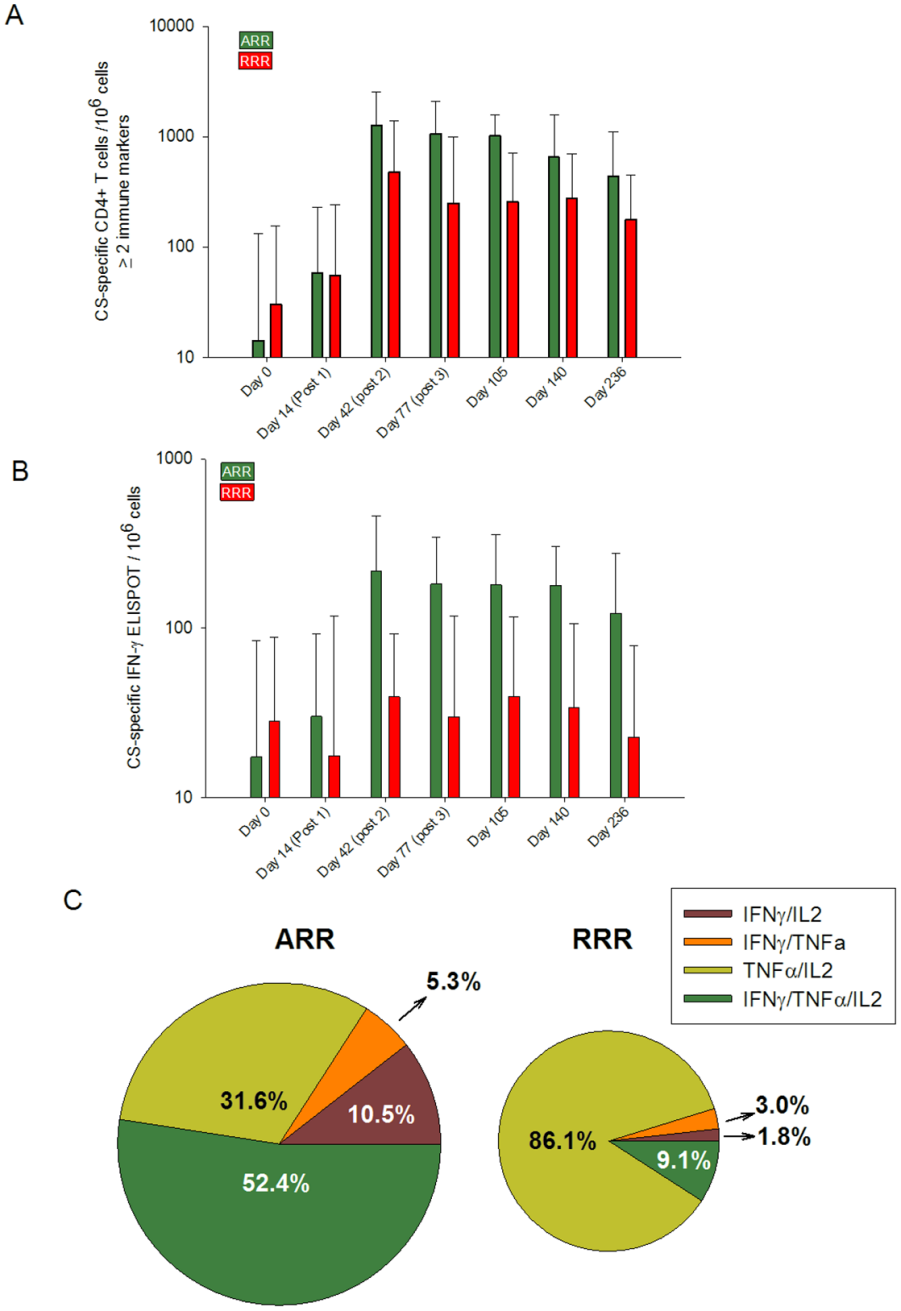
Cellular immune responses by vaccine group and days post immunization. A. Geometric mean CS-specific CD4^+^ T cells producing IFN-γ by ELISpot. Comparisons between ARR and RRR on days 42, 77, 105,140, and 236 were significant (P < .05) by Student’s *t*-test on log-transformed data. Error bars represent standard deviation of the mean. B. Geometric mean CS-specific CD4^+^ T cell responses expressing ≥2 cytokine/activation markers among IL2, IFN-γ, TNF-α, and CD40L, per million PBMC. Comparisons between ARR and RRR on days 42, 77, 105, 140 were significant (P < .05) by Student’s *t*-test on log-transformed data. Error bars represent standard deviation of the mean. C. Pie charts showing the pattern of CS-specific polyfunctional CD4^+^ CD40L^+^ T cells expressing ≥2 cytokine markers at Day 77 (day of challenge). Each slice within the pie chart represents a specific combination of two or three cytokines. The size of the pie chart is proportional to the magnitude of the response.

CS-specific IFN-γ ELISpot responses were significantly greater in the ARR group as compared to the RRR group at all time points from day 42 (post dose 2) through day 236 (Fig 3A). CS-specific CD4 T cell responses expressing two or more immune markers among CD40L, IL-2, TNF-α and IFN-γ were induced post vaccination in both vaccine groups, and T cell responses were significantly higher in the ARR group compared to the RRR group from day 42 (post dose 2) to day 140 (Fig 3B). There was a trend towards more simultaneous expression of cytokine/activation markers (polyfunctionality) and differentiation towards IFN-γ production in the ARR group as compared to the RRR group. The proportion and magnitude of these polyfunctional CD4 T cell responses differed between the two vaccine groups (Fig 3C).

Low CS-specific CD8+ T-cells expressing at least 2 cytokines/activation markers were observed from post Dose 2 in the ARR group (S2 Table). No significant CD8+ T-cell responses were observed in the RRR group and no statistical differences between vaccine groups were observed at any time point.

#### Anti-CS antibody and cell-mediated immune responses by protection

**Fig 4:**
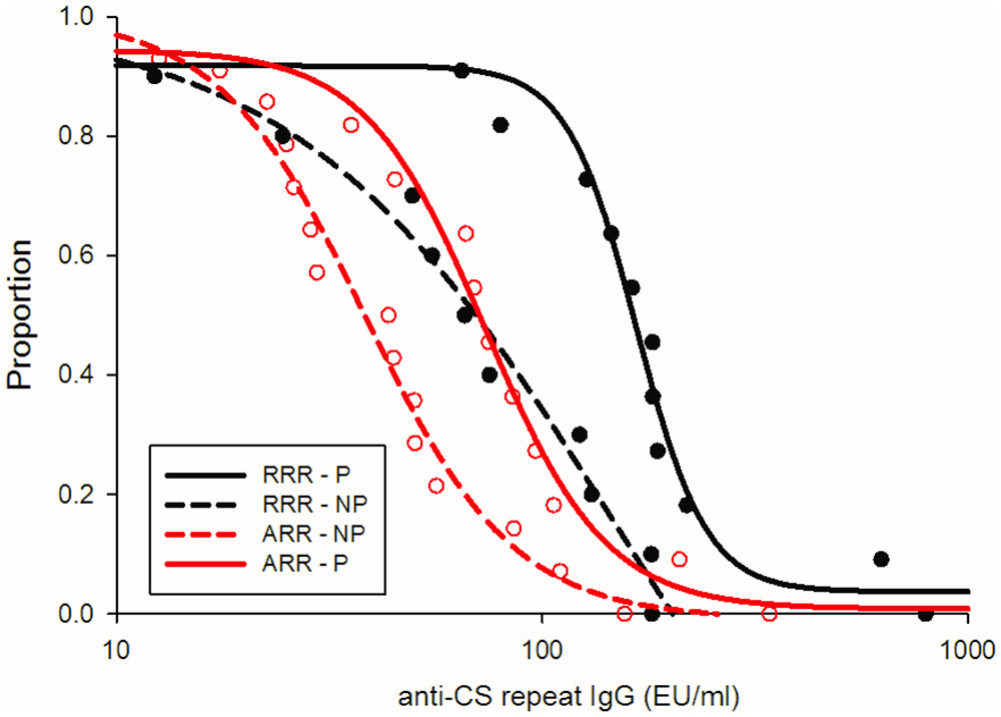
Reverse cumulative distribution curves by post challenge protection status for CS anti-repeat antibodies on day 77-Day of challenge for subjects in ARR protected (solid red) and non-protected (dashed red) compared to RRR protected (solid black) and non-protected (dashed black) subjects. *Y*-axis represents proportion of subjects at each antibody level.

Protected subjects had higher anti-CS repeat IgG titers than non-protected subjects (geometric mean titer, 120.8 vs 51.8 EU/ml, respectively; *P* = .0013). The IgG titers were significantly higher in protected compared to non-protected subjects post Dose 3 (Day 77; DOC) in the RRR group (*P* = .01) (Table 2). Reverse cumulative distribution curves (RCDC) comparing protected and non-protected subjects in both groups confirmed these differences over a range of antibody titers (Fig 4). Of note, the RCDC of protected subjects in the ARR group overlapped with the RCDC from non-protected subjects in the RRR group suggesting that immune response(s) other than anti-CS repeat IgG or contributions from the Fc immunoglobulin domain accounted for the differences in protection between the two sub-groups.

There were no significant differences between protected and non-protected subjects in IFN-γ secretion by CS-specific T cells in ELISpot assay for the entire cohort (p = .285), nor were there any differences detected in ELISpot assays when subgroups (ARR, p = .233; RRR, p = .827) were analyzed. No significant differences at DoC in CD4^+^ T cell responses analyzed by ICS were observed in protected compared to non-protected subjects when analyzed together (p = .332, not shown), or stratified by vaccine group ARR (p = .647), and RRR p = .168) (Table 3). The difference in CD8+ T-cell responses at DoC in protected compared to non-protected subjects in the ARR group was not significant (p = .7631) (S2 Table).

**Table 3:** Frequency of CS-specific CD4+ T cells expressing at least 2 cytokines/activation markers per million PBMC, by treatment group and protection status (ATP cohort for immunogenicity).

Days	ARR Protection Status (N)	ICS (95% CI)	P value (NP vs P)	RRR Protection Status (N)	ICS (95% CI)	P value (NP vs P)
D0 (screening)	NP (14)	14 (3,63)	.822	NP (9)	49 (8,316)	.341
	P (11)	18 (3, 100)		P (10)	17 (3,100)	
D14 (Post 1)	NP (14)	33 (12,100)	.011	NP (9)	123 (32,501)	.210
	P (11)	170 (100, 316)		P (9)	25 (3,251)	
D42 (Post 2)	NP (14)	1380 (1000,1995)	.384	NP (9)	490 (200,1259)	.908
	P (11)	1023 (501,1995)		P (10)	457 (200,1000)	
D77 (Post 3, DoC)	NP (12)	1000 (631,1585)	.647	NP (8)	537 (200,1259)	.168
	P (9)	1148 (631,1995)		P (8)	229 (79,631)	
D236	NP (13)	355 (126,1000)	.423	NP (6)	251 (50,1259)	.482
	P (6)	693 (251,1995)		P (7)	112 (13,1000)	

ARR = first dose with Ad35.CS, and the second and third doses with RTS,S/AS01_B_

RRR = three doses with RTS,S/AS01_B_

P values calculated using log-transformed cell frequency per million PBMC in Students t-test

NP = non-protected; P = protected

One of the questions we sought to address in this trial was to assess whether cell-mediated immunity (ICS and/or IFN-γ ELISpot) contributed to protection over and above that contributed by anti-CS antibodies. Several multiple logistic regression models (see S1 Text) were prescribed in the analytic plan and the combination of ELISpot and polyfunctional T cells was chosen to represent cellular immune function. The models examined the probability of being protected against malaria by either CS antibodies alone (model 1) or the combination of CS antibodies, CD4 T-cells expressing ≥ 2 cytokine/activation markers, and IFN-γ ELISpot (model 2) (see S1 Text for details). When vaccine groups were pooled (ARR + RRR), increasing anti-CS antibody levels increases the probability of being protected (S32 Table). When the analysis by vaccine group was performed separately, the log-likelihood ratio test in the ARR group (but not RRR) was significant (*p* = .034) suggesting that the CD4 polyfunctional cells and IFN-γ ELISpot responses add significantly to the explanation of the protection probability over and above what is explained by anti-CS antibody levels alone.

## Discussion

RTS,S/AS01 is the most advanced candidate malaria vaccine and is in late stage phase 3 testing in infants and young children in Sub-Saharan Africa. Data through 12 months follow-up shows that the vaccine provides partial protection against both clinical and severe disease [22–23]. Measured vaccine efficacy may be influenced by the age at time of vaccination and/or co- administration of other pediatric vaccines part of the Expanded Programme on Immunization. Second generation vaccines should elicit higher levels of protection that persist for a longer duration. In this study, we show that a heterologous prime-boost immunization regimen comprising a first dose of Ad35.CS.01 followed by two doses of RTS,S/AS01 did not induce increased protection as compared to three doses of RTS,S/AS01.

Antibodies elicited against epitopes located within the repeat region of the CS antigen correlate with protection, as demonstrated in the CHMI model [10–13] and in field studies [20, 39–40]. This was confirmed in the present study, showing higher antibody levels in the protected individuals as compared to those who developed malaria after challenge. Ad35.CS.01 did not induce priming immune response favorable to antibody production, as anti-CS protein titers after the last immunization were lower in the ARR group as compared to the RRR group. Two rather than three doses of RTS,S/AS01 in the ARR group probably may have contributed to overall lesser amount of CS-specific IgG and lower protection in the ARR group. It remains unclear whether increasing the number of RTS,S/AS01 doses from two to three in the prime-boost regimen would have induced higher protection as compared to three doses of RTS,S/AS01 alone.

There is no quantitative metric that reliably predicts protection above a certain antibody threshold, as the overlap in antibody titers with respect to protection outcome is considerable. When applied to different clinical endpoints, some models have predicted a step-wise increase in protection associated with antibody levels in the range of 40 EU/ml [20, 39], while others have shown a continuous pattern of relationship (40). While there is a strong body of evidence for an important role of anti-CS antibodies in mediating vaccine-induced protection, other factors are involved, as demonstrated by the fact that protected subjects from the ARR group and unprotected subjects from the RRR group have identical anti-CS antibody RCDC.

We are currently investigating whether antibody affinity maturation, somatic hypermutation of CS-specific immunoglobulin genes, and other T-cell markers are correlates or surrogates of protective immunity. Systems biology approaches using gene expression and RNA sequencing analysis are also ongoing and will be reported later

Several studies showed that RTS,S vaccination induces CS-specific CD4 T-cells expressing a number of cytokines, notably IL2, IFN-γ, and TNF-α as detected by ELISpot assay or by ICS. A limited number of studies have suggested that such CD4 T cell responses correlate with protection against infection in CHMI studies [13, 25], and in children under natural malaria exposure [28–29]. With the demonstration that Ad35.CS.01 followed by booster immunizations with RTS,S/AS01 elicited 16-fold higher numbers of CS-specific CD4^+^ T-cells in rhesus macaques [35], we hypothesized that significantly increased protection over RTS,S alone would be observed using a viral prime- protein boost second generation approach.

In this study, we confirmed the original hypothesis that the magnitude of antigen-specific CD4^+^ T-cells was significantly higher in the prime-boost ARR group compared to RRR alone, however, this did not translate into increased protection. Multiple logistic regression analysis showed that when both groups are considered together, or when considering the RRR alone, antibody response was the most important factor associated with protection. When considering the ARR group only, adding IFN-γ ELISpot and polyfunctional T cells in the model constitutes a significant change relative to the model considering antibodies only, with some evidence of a positive association with protection for polyfunctional T cells but negative for IFN- γ ELISpot. This should be interpreted with caution, in light of the limited number of observations. This may nevertheless help explain why the reverse cumulative distribution curves of antibody levels in the ARR protected group were similar to the reverse cumulative distribution curves in the RRR non protected group or, in other words, why lower levels of antibody that were not protective in the RRR group were protective in the ARR group. We also cannot exclude the possibility that Ad35.CS.01 N-terminal CS amino acid sequences, lacking in RTS, may have immunologically primed ARR subjects, thus contributing to a small component of protective immunity against challenge. CD4 themselves may be involved in protection or they may be associated with production of an antibody that is more protective or both. This warrants further investigation. In a past study, heterologous prime-boost immunization with RTS,S/AS02A and the poxvirus MVA-CS induced marginally increased T cell responses and no increased antibody response, with no improvement on protection in the CHMI model, as compared to RTS,S/AS02 alone [41]. It would be premature to imply from the results presented here that CD4^+^ T-cells do not contribute to inducing a protective immune response. First, the number of subjects in the study is small, and the analysis of CD4 responses may need to take HLA into account.

Second, CD4^+^ T-cells may be active in an anatomical compartment other than that detectable in the blood. Third, CD4^+^ cells displaying phenotype/functions other than that evaluated here may play a role. Fourth, a threshold level of CD4^+^ cells may need to be reached in order to observe a significant impact on efficacy and this threshold may not have been achieved in this study.

In contrast to preclinical studies [42], CD8^+^ T-cell activation was only marginally increased in the ARR group only, and it remains to be seen whether CS-specific CD8^+^ T-cell responses can protect vaccinated subjects against malaria. In light of this study, it is prudent to consider alternative vaccine strategies to improve the efficacy and increase the durability of protective immune responses. Future strategies to improve upon RTS,S-induced protection may need to utilize alternative highly immunogenic prime-boost regimens and/or additional target antigens.

## Supporting Information

S1 CONSORT Checklist CONSORT Checklist. (PDF)

S1 Fig Summary of solicited local and general AEs. Paired columns denote percentage of doses of AEs by intensity occurring within 7 days of immunization for subjects in ARR (yellow) and RRR (green) groups. Grade 3 AEs are denoted by orange bars.
(DOCX)

S1 Protocol Study protocol. (PDF)

S1 Table Unsolicited symptoms following dose 1. (DOCX)

S2 Table Frequency of CS-specific CD8+ T-cells expressing at least 2 cytokines/activation markers between IL- 2, IFN-γ, TNF-α and CD40L, per million PBMC (ATP cohort for immunogenicity) (DOCX)

S3 Table Logistic regression analysis modeling the probability of being protected. (DOCX)

S1 Text Additional information provided on safety analysis, immunomonitoring, statistical analysis, and eligibility criteria to support data and conclusions in manuscript. Supplementary tables and figure are available within in the Supplementary Information files.
(DOCX)
