# Supplementary material for: Ad35.CS.01 - RTS,S/AS01 Heterologous Prime Boost Vaccine Efficacy against Sporozoite Challenge in Healthy Malaria-Naïve Adults
Source: PLoS One. 2015 Jul 6;10(7):e0131571. doi: 10.1371/journal.pone.0131571 (PMC4492580; doi:10.1371/journal.pone.0131571)
Supplement: S1 Fig — Paired columns denote percentage of doses of AEs by intensity occurring within 7 days of immunization for subjects in ARR (yellow) and RRR (green) groups. Grade 3 AEs are denoted by orange bars. (DOCX) [file pone.0131571.s002.docx]

**S1 Fig. Summary of solicited local and general AEs.**


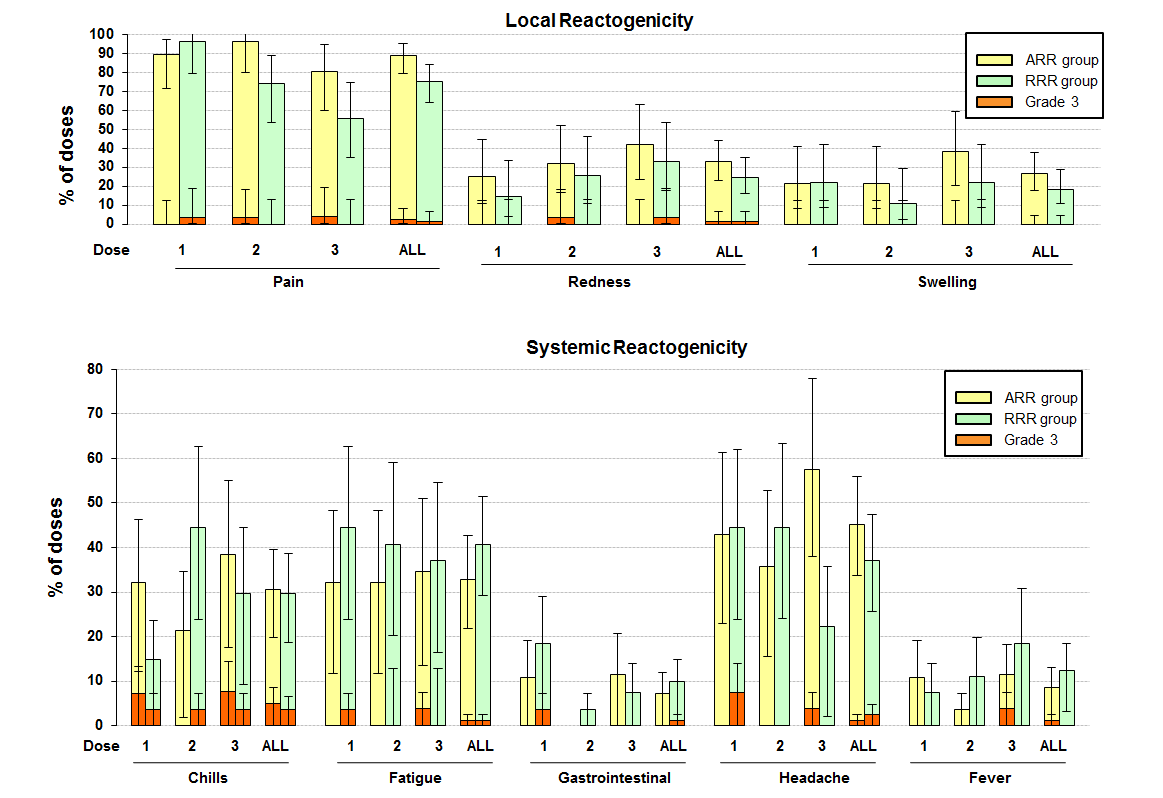


Paired columns denote percentage of doses of AEs by intensity occurring within 7 days of immunization for subjects in ARR (yellow) and RRR (green) groups. Grade 3 AEs are denoted by orange bars. Safety criteria are defined in Supplementary Data text.
