# Supplementary material for: Ad35.CS.01 - RTS,S/AS01 Heterologous Prime Boost Vaccine Efficacy against Sporozoite Challenge in Healthy Malaria-Naïve Adults
Source: PLoS One. 2015 Jul 6;10(7):e0131571. doi: 10.1371/journal.pone.0131571 (PMC4492580; doi:10.1371/journal.pone.0131571)
Supplement: S1 Protocol — (PDF) [file pone.0131571.s003.pdf]

Clinical Study Protocol
Sponsor:
GlaxoSmithKline Biologicals
89 Rue de l'Institut
Rixensart, Belgium

**Primary Study vaccine and number**	GlaxoSmithKline (GSK) Biologicals' candidate *Plasmodium falciparum* malaria vaccine RTS,S/AS01$_B$ (257049)
**Other Study vaccine**	Crucell's replication deficient adenovirus type 35 circumsporozoite malaria vaccine (Ad35.CS.01)
**eTrack study number and Abbreviated Title**	114460 (Malaria-068)
**Investigational New Drug (IND) number**	***BB-IND-14701*** **(Amended 09 June 2011)**
**Date of protocol**	03 February 2011
**Date of protocol amendment 1**	Amendment 1 Final: 09 June 2011
**Title**	Safety, immunogenicity and efficacy against malaria in the sporozoite challenge model of one dose of Ad35.CS.01 malaria vaccine followed by two doses of malaria 257049 vaccine in healthy malaria-naïve adults.
**Detailed Title**	Phase I/IIa, observer-blind, randomized, controlled human challenge study, to evaluate safety, reactogenicity, immunogenicity and efficacy of Crucell's candidate replication deficient adenovirus type 35 circumsporozoite malaria vaccine administered intramuscularly followed one month later by two doses of GSK Biologicals' candidate malaria vaccine RTS,S/AS01$_B$ administered intramuscularly at one month intervals, compared to three doses of RTS,S/AS01$_B$ administered intramuscularly at one month intervals in healthy malaria-naïve volunteers aged 18-50 years.
**Co-ordinating author**	Liliana Manciu, Scientific Writer
**Contributing authors**	• Ripley Ballou, Vice-President & Head Clinical Research & Translational Science<br>• Yolanda Guerra, Manager Safety Physician, Clinical Safety and Pharmacovigiliance<br>• Erik Jongert, Clinical Immunology Read Outs Manager, R&D

- Marc Lievens, Bio-Statistician Manager, Global Clinical R&D
- Saartje Vansteenkiste, Global Study Manager
- Shantala Rao, Clinical Data Coordinator
- Johan Vekemans, Director, Clinical Development (Malaria Vaccine)
- Pascale Vandoolaeghe, Senior Scientist, Clinical Regulatory, Clinical Reg. & Labelling

GSK Biologicals' Protocol DS v 13.2

Copyright 2011 the GlaxoSmithKline group of companies. All rights reserved. Unauthorised copying or use of this information is prohibited.

Protocol Amendment 1 Sponsor Signatory Approval

**eTrack study number and Abbreviated Title**	114460 (Malaria-068)
**IND number**	***BB-IND-14701*** **(Amended 09 June 2011)**
**Date of protocol**	03 February 2011
**Date of protocol amendment 1**	Amendment 1: Final 09 June 2011
**Detailed Title**	Phase I/IIa, observer-blind, randomized, controlled human challenge study, to evaluate safety, reactogenicity, immunogenicity and efficacy of Crucell's candidate replication deficient adenovirus type 35 circumsporozoite malaria vaccine administered intramuscularly followed one month later by two doses of GSK Biologicals' candidate malaria vaccine RTS,S/$AS01_B$ administered intramuscularly at one month intervals, compared to three doses of RTS,S/$AS01_B$ administered intramuscularly at one month intervals in healthy malaria-naïve volunteers aged 18-50 years.
**Sponsor signatory**	______________________________
**Signature**	______________________________
**Date**	______________________________

For internal use only

- ----------------Checksum----------------!Ver.!Created On - -
ac77581444799105ae4098b6a1e70d5be1264a8b 2.0 6/15/2011 8:43:00 AM - -
-- -

Protocol Amendment 1 Rationale

**Amendment number:** Amendment 1
**Rationale/background for changes:**<br><br>In order to reduce the risk of high reactogenicity reactions and in line with a FDA recommendation, the Ad35.CS.01 dose for injection was lowered from $1x10^{11}$ vp to $5x10^{10}$ vp in Table 21.<br><br>In addition, the following minor changes have been included:<br><br>• The IND number has been added on the Title page, Sponsor and Investigator Signatory pages.<br>• Blood sampling for smear and PCR was deleted at Visit 41 in Synopsis Table 4, Table 6. Consequently, the volume of blood to be collected at Visits 41 and 42 has been corrected throughout the protocol (i.e. Synopsis Table 4, Tables 6, 10 and 13).<br>• The reference to Table 27 has been corrected to Table 28 in Section 4.4. In addition, a note has been added to clarify that NHANES I criteria will be applied for all subjects including subjects aged 18-35 years old.<br>• The trade mark for Malarone has been added in Section 5.4.2.2.<br>• Information concerning timings post-challenge for a volunteer who develops malaria and has 3 consecutive negative smears following initial treatment has been corrected in Section 5.4.3.5 and Table 9.<br>• Information on weekly evaluations after malaria treatment has been deleted in Section 5.4.3.6 as these weekly visits are usually not conducted as part of the standard sporozoite challenge model.<br>• One re-bleeding during screening, as an acceptable way to refine or confirm whether a potential participant is eligible or not, or to complete an insufficient blood volume is allowed, when medically justified, at investigators discretion. Examples include a clotted or hemolysed sample, or transient borderline abnormal blood values considered non-clinically significant. This information has been added in Table 7.<br>• One re-bleeding at each time point that a blood sample is taken during the study, after the screening epoch, is also allowed, if necessary, at investigators discretion to cover for technical issues (insufficient blood volume, clotted sample, hemosysis…). This information has been added in Tables 8, 9 and 10.<br>• One urine re-sampling is allowed, if necessary, at each time point that a urine sample is taken during the study, at investigators discretion, to cover for technical issues and guarantee a clean urine catch. This information has been added in Tables 7 and 8.<br>• Information on the follow-up period for non-serious AEs has been deleted in Table 8.

- The order in which study procedures related to the assignment of study subject number and the check of inclusion/exclusion criteria are presented has been switched in Table 7 and Section 5.6.2 to better reflect what will actually be done.
- Information on cytokine/chemokines analysis has been corrected throughout the protocol (i.e. Section 5.6.2.8, Table 15 and Appendix C) as this analysis will be performed on serum instead of plasma.
- Information concerning the temperature storage for the serum to be used for cytokine/chemokines analysis has been modified from -20°C to -80°C in Section 5.6.2.8 and Appendix C.
- Section 5.6.3.9 has been modified with the purpose to standardize reactogenicity data collection on the diary cards.
- The laboratory performing deep sequencing has been updated in Table 16 from ISB to Seattle Biomed.
- Table 20 has been modified to add Visit 16 (D57) timepoint which was erroneously missing.
- Information on the diluent to be used to dilute Ad35.CS.01 to the intended dose for the study ($5x10^{10}$ vp instead of $1x10^{11}$ vp per injected dose) has been added in Table 21 and Sections 6.2.2 and 6.3.2.2.
- Temperature deviation for the Ad35.CS.01 vaccine has to be reported to the sponsor. Once the sponsor becomes aware of a temperature deviation, he must inform Crucell IMMEDIATELY. This information has been corrected in Section 6.2.2.
- The time window for recording concomitant medication has been corrected in Section 6.7.6.2 to extend it to the screening period (as specified in Table 7).
- Information concerning the number of timepoints for microarray assays has been corrected in Appendix C from 15 to 14 timepoints (as specified in Table 6).
- Minor corrections such as clarifications and typos have been made in Appendix B, Section 5.4.2.1.

Protocol Amendment 1 Investigator Agreement

I agree:

- To conduct the study in compliance with this protocol, any mutually agreed future protocol amendments or protocol administrative changes, and with any other study conduct procedures and/or study conduct documents provided by GlaxoSmithKline Biologicals (GSK Biologicals).
- To assume responsibility for the proper conduct of the study at this site.
- That I am aware of, and will comply with, ‘Good Clinical Practice’ (GCP) and all applicable regulatory requirements.
- To ensure that all persons assisting me with the study are adequately informed about the GSK Biologicals’ investigational product(s) and other study-related duties and functions as described in the protocol.
- To acquire the reference ranges for laboratory tests performed locally and, if required by local regulations, obtain the laboratory’s current certification or Quality Assurance procedure manual.
- To ensure that no clinical samples (including serum samples) are retained onsite or elsewhere without the approval of GSK Biologicals and the express written informed consent of the subject and/or the subject’s legally authorised representative.
- To perform no other biological assays on the clinical samples except those described in the protocol or its amendment(s).
- To co-operate with a representative of GSK Biologicals in the monitoring process of the study and in resolution of queries about the data.
- That I have been informed that certain regulatory authorities require the sponsor to obtain and supply, as necessary, details about the investigator’s ownership interest in the sponsor or the investigational product, and more generally about his/her financial ties with the sponsor. GSK Biologicals will use and disclose the information solely for the purpose of complying with regulatory requirements.

Hence I:

- Agree to supply GSK Biologicals with any necessary information regarding ownership interest and financial ties (including those of my spouse and dependent children).
- Agree to promptly update this information if any relevant changes occur during the course of the study and for one year following completion of the study.
- Agree that GSK Biologicals may disclose any information it has about such ownership interests and financial ties to regulatory authorities.
- Agree to provide GSK Biologicals with an updated Curriculum Vitae and other documents required by regulatory agencies for this study.

**eTrack study number and Abbreviated Title**	114460 (Malaria-068)
**IND number**	***BB-IND-14701*** **Amended 09 June 2011)**
**Date of protocol**	03 February 2011
**Date of protocol amendment 1**	Amendment 1 Final: 09 June 2011
**Detailed Title**	Phase I/IIa, observer-blind, randomized, controlled human challenge study, to evaluate safety, reactogenicity, immunogenicity and efficacy of Crucell's candidate replication deficient adenovirus type 35 circumsporozoite malaria vaccine administered intramuscularly followed one month later by two doses of GSK Biologicals' candidate malaria vaccine RTS,S/AS01$_B$ administered intramuscularly at one month intervals, compared to three doses of RTS,S/AS01$_B$ administered intramuscularly at one month intervals in healthy malaria-naïve volunteers aged 18-50 years.
**Investigator name**	______________________
**Signature**	______________________
**Date**	______________________

For internal use only

- ----------------Checksum----------------!Ver.!Created On - -
ac77581444799105ae4098b6a1e70d5be1264a8b 2.0 6/15/2011 8:43:00 AM - -
-- -

SYNOPSIS

**Detailed Title**	Phase I/IIa, observer-blind, randomized, controlled human challenge study, to evaluate safety, reactogenicity, immunogenicity and efficacy of Crucell's candidate replication deficient adenovirus type 35 circumsporozoite malaria vaccine administered intramuscularly followed one month later by two doses of GSK Biologicals' candidate malaria vaccine RTS,S/AS01$_B$ administered intramuscularly at one month intervals, compared to three doses of RTS,S/AS01$_B$ administered intramuscularly at one month intervals in healthy malaria-naïve volunteers aged 18-50 years.
**Indication**	Primary immunization of infants and children living in malaria endemic areas for the prevention of malaria disease due to *Plasmodium falciparum* (*P. falciparum*). The indication of the immunization regimen may, depending on the risk/benefit profile, be extended to a wider age group.
**Rationale for the study and study design**	• Rationale for the study<br><br>GSK Biologicals in collaboration with MVI and Crucell is developing an Ad35.CS.01 prime-RTS,S/AS01$_B$ boost immunization regimen for prevention of malaria due to *P. falciparum* in infants and children living in malaria endemic areas.<br><br>While both RTS,S/AS01$_B$ and Ad35.CS.01 candidate malaria vaccines tested in this study have been administered to humans before, this trial will be the first one where they are combined in one immunization schedule.<br><br>• Rationale for the study design<br><br>This study is designed to evaluate safety, reactogenicity, immunogenicity and efficacy of Crucell's candidate replication deficient adenovirus type 35 circumsporozoite malaria vaccine (Ad35.CS.01) administered intramuscularly followed one month later by two doses of GSK Biological's malaria candidate vaccine RTS,S/AS01$_B$ administered intramuscularly at one month intervals, compared to three doses of RTS,S/AS01$_B$ administered intramuscularly at one month intervals in healthy malaria-naïve volunteers aged 18 to 50 years. The sporozoite challenge model, in the RTS,S/AS candidate vaccine development program, has demonstrated a high relevance in its ability to predict efficacy under conditions of natural exposure in malaria-endemic countries. Approximately 168 participants will be enrolled in the study.

Three cohorts of approximately equal size will be enrolled, vaccinated then challenged separately. This design takes into account logistical restrictions on the size of human challenge procedures and allows for a futility analysis to be conducted after the results of the malaria challenge in the first cohort. The proposed study design is such that the sample size will provide 80% power to demonstrate a 50% increase in efficacy of an immunization regimen including an initial dose of Ad35.CS.01 over efficacy of a regimen based on RTS,S/AS01$_B$ alone, assuming 50% vaccine efficacy of the RTS,S/AS01$_B$ group over challenge infectivity controls.

The study design is similar to that of other past studies successfully conducted. Healthy adults will be enrolled and followed up in closely controlled conditions by investigators experienced in *P. falciparum* challenge studies. Immunological investigations are planned with the intent to characterize qualitatively and quantitatively the immune response induced by the immunization regimen and assess the presence of a correlation of specific effector immune function(s) with protection against malaria infection in the sporozoite challenge model. Protection will be evaluated by the proportion of immunized participants who remain free of *P. falciparum* infection following sporozoite challenge and by a delay in the pre-patent period leading to infection.

Objectives

Primary

- To compare the efficacy (occurrence of *P. falciparum* parasitemia, assessed by blood slide) of an immunization regimen comprising of one dose of Ad35.CS.01 followed one month later by two doses of RTS,S/AS01$_B$ administered at one month intervals, with that of three doses of RTS,S/AS01$_B$ administered at one month intervals, in healthy malaria-naïve volunteers aged 18-50 years in the sporozoite challenge model.
- To assess the safety (reactogenicity, unsolicited adverse events, SAEs up to 30 days post last vaccination [day of vaccination and 29 subsequent days], SAEs up to study conclusion) of an immunization regimen comprising of one dose of Ad35.CS.01 followed one month later by two doses of RTS,S/AS01$_B$ administered at one month intervals in healthy malaria-naïve volunteers aged 18-50 years.

Secondary

- To compare the efficacy (time to *P. falciparum* parasitemia, assessed by blood slide) of an immunization regimen comprising of one dose of Ad35.CS.01 followed one month later by two doses of RTS,S/AS01$_B$ administered at one month intervals, with that of three doses of RTS,S/AS01$_B$ administered at one month intervals, in healthy malaria-naïve volunteers aged 18-50 years in the sporozoite challenge model.
- To assess the humoral and cell-mediated immune response to malaria and hepatitis B antigens (by ELISA, ICS and ELISPOT) induced by one dose of Ad35.CS.01 followed one month later by two doses of RTS,S/AS01$_B$ administered at one month intervals in healthy malaria-naïve volunteers aged 18-50 years.
- To assess the humoral immune response to adenovirus antigen induced by one dose of Ad35.CS.01 followed one month later by two doses of RTS,S/AS01$_B$ administered at one month intervals in healthy malaria-naïve volunteers aged 18-50 years.

Exploratory objectives

- To compare the efficacy (occurrence of *P. falciparum* parasitemia and time to *P. falciparum* parasitemia, assessed by PCR) of an immunization regimen comprising of one dose of Ad35.CS.01 followed one month later by two doses of RTS,S/AS01$_B$ administered at one month intervals, with that of three doses of RTS,S/AS01$_B$ administered at one month intervals, in healthy malaria-naïve volunteers aged 18-50 years in the sporozoite challenge model.
- To assess the immune response to malaria, hepatitis B and adenovirus antigens (by various methods including high-throughput technologies such as microarray analysis and deep sequencing, as part of a systems biology approach) induced by one dose of Ad35.CS.01 followed one month later by two doses of RTS,S/AS01$_B$ administered at one month intervals in healthy malaria-naïve volunteers aged 18-50 years.

Study design

- Experimental design: Phase I/II a, observer-blind, randomised, controlled, multi-centric, single-country study with two parallel groups and infectivity controls.
- Duration of the study: For each vaccinated subject, the study duration will be approximately 11 months. For each

infectivity control subject, the study duration will be approximately 8 months.

- Screening epoch: Screening (and enrollment) starting Visit 1 (Day -90 to -3) and ending Visit 2 (Day -7 to -1).
- Vaccination epoch: Vaccination starting Visit 3 (Day 0) and ending Visit 19 (Day 62)
- Challenge epoch: Challenge starting Visit 20 (Day 77) and ending Visit 40 (Day 105).
- Follow-up epoch: Follow-up starting Visit 41 (Day 140) and ending Visit 42 (Day 236).

Synopsis Table 1 Study groups and epochs foreseen in the study

Study groups	Number of subjects	Age (Min/Max)	Epochs			
			Screening epoch	Vaccination epoch	Challenge epoch	Follow-up epoch
RRR	84	18 - 50 years	x	x	x	x
ARR	84	18 - 50 years	x	x	x	x
Infectivity controls	4 - 6 per challenge day	18 - 50 years	x		x	x

ARR = first dose with Ad35.CS.01, second and third doses with RTS,S/AS01$_B$
RRR = three doses of RTS,S/AS01$_B$

- Control: active control. The group receiving three doses of RTS,S/AS01$_B$ will be the comparator group. In addition, there will be infectivity controls for the challenge epoch (i.e. volunteers who will not receive any immunization but will be subjected to the sporozoite challenge).
- Vaccination schedule: 0-1-2 months followed by a challenge 21 days (3 weeks) ± 7 days after the third vaccination.
- Safety considerations:

  Vaccination and challenge will be performed in a staggered fashion. Three cohorts (Cohort A, B and C) of approximately equal size will be enrolled, vaccinated and challenged separately. Each cohort which enters the vaccination phase will include approximately 56 subjects. Among the 56 vaccinated individuals a random selection of subjects eligible for challenge will be done according to the local SOP to ensure that a maximum of 46 vaccinated individuals (balanced by group) will progress to the challenge phase. The remaining subjects will not be challenged but they will be followed for safety and immunogenicity up to 6 months after the last vaccination (Study Day 236). Vaccination and challenge will be performed first in Cohort A. Safety and efficacy data post-

challenge from this initial cohort and 4 to 6 infectivity controls will be reviewed prior to the start of vaccination in Cohorts B and C, based on pre-defined group holding criteria for safety review (see Section 5.4.4.1) and pre-defined criteria for efficacy futility analysis (see Section 5.4.4.2). Vaccination and challenge of subjects in Cohorts B and C is likely to take place in parallel at two different sites (see Section 4.2 for more details).

In addition, the Principal Investigator (PI) will have the responsibility to withdraw from further vaccination individual study participants who present at least one of the pre-defined stopping rules for individual subjects (see Section 5.4.4.1).

- Safety and immunogenicity will be evaluated during the study through 6 months after the last vaccination (Study Day 236).
- Study groups: refer to Synopsis Table 2

Synopsis Table 2 Study groups

Treatment Identifier	Vaccine/Product name	Study groups		
		RRR	ARR	Infectivity controls
Ad35.CS.01	Ad35.CS.01	-	x	-
RTS,S/AS01B	RTS,S	x	x	-
	AS01B	x	x	-

- Treatment allocation: randomised (1:1).
- Blinding: observer-blind as to immunization regimens (see Synopsis Table 3).

Synopsis Table 3 Blinding of study epochs

Study Epochs	Study groups	Blinding
Screening epoch	Immunization groups	N/A
	Infectivity controls	N/A
Vaccination epoch	Immunization groups	observer-blind
	Infectivity controls	N/A
Challenge epoch	Immunization groups	observer-blind
	Infectivity controls	open
Follow-up epoch	Immunization groups	open
	Infectivity controls	open

N/A: Not applicable

- Blood sampling: refer to Synopsis Table 4.

Synopsis Table 4 Sampling timepoints and volume of blood collected

Epoch	Screening		Vaccination											
**Study Day**	**-90 to -3**	**-7 to-1**	**0**	**1**	**2**	**6**	**14**	**28**	**29**	**34**	**42**	**56**	**57**	**62**
**Visit number**	**1**	**2**	**3**	**4**	**5**	**7**	**8**	**9**	**10**	**13**	**14**	**15**	**16**	**19**
Safety labs (7ml)*	7		7			7		7		7		7		7
Additional screening labs (15 ml)**	15													
Humoral response (20 ml)		20						20				20		
Cell-mediated immunity assays (60 ml)		60					60				60			
Exploratory cytokine/chemokine analysis (Luminex) (2 ml)		2		2	2	2	2	2	2	2		2	2	2
Microarray and deep sequencing analysis (12 ml)		12		12	12	12	12	12	12	12		12	12	12
Plasmablasts FACS and B cell ELISPOT (10 ml)		10						10		10		10		10
**Blood volume in mL per visit**	**22**	**104**	**7**	**14**	**14**	**21**	**74**	**51**	**14**	**31**	**60**	**51**	**14**	**31**
Cumulative blood vol. - Immunized groups (ml)	22	126	133	147	161	182	256	307	321	352	412	463	477	508
Cumulative blood vol. – Infectivity controls (ml)	22	-	-	-	-	-	-	-	-	-		-	-	-

*Safety labs include hemoglobin, WBC, PLT, ALT, AST, creatinine. **Additional screening labs include HIV, HCV, HBV.

Epoch	Challenge																				Follow-up	
	DoC*	Early Post-challenge					Hotel/inpatient Phase										Late Post-challenge				Final Visits	
**Study day**	**77**	**78**	**82**	**83**	**84**	**85**	**86**	**87**	**88**	**89**	**90**	**91**	**92**	**93**	**94**	**95**	**97**	**99**	**101**	**103**	**105**	**140**	**236**
**Challenge day**	**0**	**1**	**5**	**6**	**7**	**8**	**9**	**10**	**11**	**12**	**13**	**14**	**15**	**16**	**17**	**18**	**20**	**22**	**24**	**26**	**28**	**63**	**159**
**Visit number**	**20**	**21**	**22**	**23**	**24**	**25**	**26**	**27**	**28**	**29**	**30**	**31**	**32**	**33**	**34**	**35**	**36**	**37**	**38**	**39**	**40**	**41**	**42**
Safety labs (7 ml)**	7													7***							7	7	7
Smear and PCR (2 ml)			2	2	2	2	2	2	2	2	2	2	2	2	2‡	2‡	2‡	2‡	2‡	2‡	2‡		
Humoral immunity (ELISA) (20 ml)	20																				20	20	20
Cellular immunity (60 mL)	60																				60	60	60
Exploratory cytokine/chemokine analysis (Luminex) (2 ml)	2	2	2																		2		
Microarray and deep sequencing analysis (12 ml)	12	12	12																				
**Blood volume in ml per visit**	**101**	**14**	**16**	**2**	**2**	**2**	**2**	**2**	**2**	**2**	**2**	**2**	**2**	**9****	**2**	**2**	**2**	**2**	**2**	**2**	**91**	**87**	**87**
Cumulative blood vol. - Immunized groups (ml)	609	623	639	641	643	645	647	649	651	653	655	657	659	668	670	672	674	676	678	680	771	858	945
Cumulative blood vol. – Infectivity controls (ml)	123	137	153	155	157	159	161	163	165	167	169	171	173	182	184	186	188	190	192	194	285	372	459

*DoC: day of challenge. **Safety labs include hemoglobin, WBC, PLT, ALT, AST, creatinine. ***Day of parasitemia and number of parasitemic individuals is undetermined.
‡ For volunteers who develop malaria: blood smears and PCR may be discontinued once the volunteer has 3 consecutive (separated by greater than 12 hours) negative smears following initial treatment. **(Amended 09 June 2011)**

- Type of study: self-contained
- Data collection: Electronic Case Report Form (eCRF)

Number of subjects

The target enrollment for this study will be 168 healthy malaria-naïve adult subjects (84 subjects per group), to ensure 138 subjects will undergo sporozoite challenge (69 subjects per group), given an approximate 20% estimated drop-out rate, based on past experience. Additional volunteers to serve as infectivity controls will be enrolled. Four to six volunteers (not immunized) will be enrolled for each sporozoite challenge day.

Endpoints

Primary

Primary efficacy endpoint

- Occurrence of *P. falciparum* parasitemia, defined by a positive blood slide, following sporozoite challenge.

Primary safety endpoints

- Occurrence of each solicited adverse event within 7-day follow-up period (day of vaccination and 6 subsequent days) after each vaccination .
  - Solicited local adverse event (any, grade 3).
  - Solicited general adverse event (any, grade 3, related).
- Occurrence of unsolicited adverse events within 30 days (day of vaccination and 29 subsequent days) after each vaccination, according to the Medical Dictionary for Regulatory Activities (MedDRA) classification.
- Occurrence of unsolicited adverse events within 30 days (day of challenge and 29 subsequent days) after challenge, according to the Medical Dictionary for Regulatory Activities (MedDRA) classification.
- Occurrence of serious adverse events (SAEs) within 30 days (day of vaccination and 29 subsequent days) after each vaccination, according to the Medical Dictionary for Regulatory Activities (MedDRA) classification.
- Occurrence of SAEs during the whole study period (from dose 1 up to study conclusion), according to the Medical Dictionary for Regulatory Activities (MedDRA) classification.

Secondary

Secondary efficacy endpoint

- Time to *P. falciparum* parasitemia, defined by a positive blood slide, following sporozoite challenge..

Secondary immunogenicity endpoints

- Anti-CS (RT) and anti-HBs antibody titres at specified time points.
- Anti-Ad35 neutralizing antibody titers at specified time points.
- Frequency of CS (total CS or RT)-specific, HBs-specific T cells at specified time points.

Exploratory

Exploratory efficacy endpoints

- Occurrence of *P. falciparum* parasitemia, defined by a positive PCR, following sporozoite challenge.
- Time to *P. falciparum* parasitemia, defined by a positive PCR, following sporozoite challenge.

Exploratory immunogenicity endpoints

- Frequency of Ad35-specific T cells at specified time points.
- Antibody titres against full-length CS and CS specific peptides other than repeat.
- Isotype, affinity and functionality of CS-specific antibodies.
- Multivariant analysis (systems biology approach) using high-throughput technologies such as gene expression profiling, multiplex analysis of cytokines and chemokines (Luminex), and multiparameter flow cytometry for plasmablast analysis and B cell ELISPOT.

TABLE OF CONTENTS

PAGE

SYNOPSIS 8

LIST OF ABBREVIATIONS 24

GLOSSARY OF TERMS 27

TRADEMARKS 31

1. INTRODUCTION 32
    1.1. Background 32
        1.1.1. RTS,S candidate vaccine 33
        1.1.2. CS-expressing recombinant human adenovirus 35 vaccine 33
    1.2. Rationale for the study and study design 37
        1.2.1. Rationale for the study 37
        1.2.2. Rationale for the study design 37

2. OBJECTIVES 38
    2.1. Primary objective 38
    2.2. Secondary objectives 38
    2.3. Exploratory objectives 38

3. STUDY DESIGN OVERVIEW 39

4. STUDY COHORT 43
    4.1. Number of subjects/centers 43
    4.2. Overview of the recruitment plan 43
    4.3. Inclusion criteria for enrollment 43
    4.4. Exclusion criteria for enrollment 44

5. CONDUCT OF THE STUDY 46
    5.1. Regulatory and ethical considerations, including the informed consent process 46
    5.2. Subject identification and randomization of treatment 47
        5.2.1. Subject identification 47
        5.2.2. Randomization of treatment 47
            5.2.2.1. Randomization of supplies 47
            5.2.2.2. Treatment allocation to the subject 47
    5.3. Method of blinding 48
    5.4. General study aspects 48
        5.4.1. Screening 48
        5.4.2. Risks to the subjects and precautions to minimize risk 49
            5.4.2.1. Risks associated with vaccination and mitigation of risks 49
            5.4.2.2. Risks associated with malaria challenge and mitigation of risks 50
        5.4.3. Malaria Challenge 51
            5.4.3.1. Contraindications to malaria challenge 52
            5.4.3.2. Parasite and mosquito strains 53

5.4.3.3. Infection of Human Volunteers ... 53
5.4.3.4. Determining Parasitemia ... 53
5.4.3.5. Management of Infected Human Volunteers ... 54
5.4.3.6. Malaria Treatment ... 55
5.4.4. Safety monitoring plan ... 56
5.4.4.1. Safety review ... 57
5.4.4.1.1. Data reviewed by the SMC on a real-time basis ... 57
5.4.4.1.2. Data review by the SMC between vaccine doses (for each Cohort separately only) ... 57
5.4.4.1.3. Cohort A data review by the SMC prior to vaccinations in Cohort B and Cohort C: ... 58
5.4.4.2. Efficacy futility review ... 60
5.5. Outline of study procedures ... 61
5.6. Detailed description of study procedures ... 68
5.6.1. Procedures prior to study participation ... 68
5.6.1.1. Informed consent and protocol comprehension assessment ... 68
5.6.2. Procedures during the screening epoch ... 68
5.6.2.1. Check inclusion and exclusion criteria ... 68
5.6.2.2. Assign subject number ... 68
5.6.2.3. Check electrocardiogram and NHANES I criteria ... 68
5.6.2.4. Collect demographic data ... 68
5.6.2.5. Medical history ... 68
5.6.2.6. Physical examination ... 69
5.6.2.7. Urine pregnancy test ... 69
5.6.2.8. Blood sampling for safety and immune response assessments ... 69
5.6.2.9. Urine sampling for safety assessment ... 70
5.6.2.10. Check and record concomitant medication/vaccination ... 70
5.6.2.11. Recording of SAEs ... 70
5.6.3. Procedures during the vaccination epoch (only for vaccinees) ... 70
5.6.3.1. Check and record intercurrent medical conditions ... 70
5.6.3.2. Urine pregnancy test ... 70
5.6.3.3. Check contraindications, warnings and precautions to vaccination ... 71
5.6.3.4. Assess pre-vaccination body temperature ... 71
5.6.3.5. Randomization ... 71
5.6.3.6. Treatment number assignment ... 71
5.6.3.7. Vaccination ... 71
5.6.3.8. Buccal swab sampling ... 71
5.6.3.9. Recording of AEs, SAEs and pregnancies ... 71
5.6.3.10. Standard neurological assessment ... 72
5.6.4. Procedures during challenge epoch ... 72
5.6.4.1. Distribution of emergency notification card ... 72
5.6.4.2. Urine pregnancy test ... 72

5.6.4.3. Blood sampling for PCR and smear 73
5.6.4.4. Check contraindications to challenge 73
5.6.4.5. Challenge 73
5.6.4.6. Conclusion of the vaccination and challenge epochs 73
5.6.5. Procedures during follow-up epoch 73
5.6.5.1. Study conclusion 74
5.7. Biological sample handling and analysis 74
5.7.1. Use of specified study materials 75
5.7.2. Biological samples 75
5.7.3. Laboratory assays 76
5.7.4. Biological samples evaluation 80
5.7.4.1. Blood testing plan 80
5.7.5. Immunological correlates of protection 84

6. STUDY VACCINES AND ADMINISTRATION 85
6.1. Description of study vaccines 85
6.2. Storage and handling of study vaccines 86
6.2.1. RTS,S/AS01$_B$ (0.5 mL dose) 86
6.2.2. Ad35.CS.01 (0.5 mL dose) 86
6.3. Dosage and administration of study vaccines 87
6.3.1. Injection technique 87
6.3.2. Injection instructions 87
6.3.2.1. RTS,S/AS01$_B$ (0.5 mL dose) 87
6.3.2.2. Ad35.CS.01 (0.5 mL dose) 88
6.4. Replacement of unusable vaccine doses 88
6.5. Contraindications to vaccination 89
6.5.1. Absolute contraindications to vaccination 89
6.5.2. Indications for deferral of vaccination 89
6.6. Warnings and precautions 89
6.7. Concomitant medication/vaccination 89
6.7.1. Medications/products that may lead to the elimination of a subject from ATP analyses 90
6.7.2. Time window for recording concomitant medication/vaccination in the eCRF 90
6.8. Intercurrent medical conditions that may lead to elimination from an ATP cohort 91

7. HEALTH ECONOMICS 91

8. ADVERSE EVENTS AND SERIOUS ADVERSE EVENTS 91
8.1. Safety definitions 91
8.1.1. Definition of an adverse event 91
8.1.2. Definition of a serious adverse event 92
8.1.3. Solicited adverse events 93
8.1.4. Clinical laboratory parameters and other abnormal assessments qualifying as adverse events or serious adverse events 94
8.1.5. AEs of specific interest 94
8.1.5.1. Potential immune-mediated diseases 94
8.1.5.2. New onset of neurological diseases 96

8.2. Events or outcomes not qualifying as adverse events or serious adverse events 96
8.2.1. Pregnancy 96
8.3. Detecting and recording adverse events, serious adverse events and pregnancies 96
8.3.1. Time period for detecting and recording adverse events, serious adverse events and pregnancies 96
8.3.2. Evaluation of adverse events and serious adverse events 99
8.3.2.1. Active questioning to detect adverse events and serious adverse events 99
8.3.2.2. Assessment of adverse events 100
8.3.2.2.1. Assessment of intensity 100
8.3.2.2.2. Assessment of causality 102
8.3.2.3. Assessment of outcomes 104
8.4. Reporting of serious adverse events, pregnancies and other events 104
8.4.1. Prompt reporting of serious adverse events, pregnancies and other events to GSK Biologicals, MVI and Crucell 104
8.4.2. Contact information for reporting serious adverse events and other events to GSK Biologicals 104
8.4.3. Completion and transmission of SAEs reports to GSK Biologicals, MVI and Crucell 105
8.4.3.1. Back-up system in case the electronic SAE reporting system does not work 105
8.4.3.2. Updating of SAE information after freezing of the subject's eCRF 105
8.4.4. Completion and transmission of pregnancy reports to GSK Biologicals 106
8.4.5. Reporting of pIMDs to GSK Biologicals 106
8.4.6. Regulatory reporting requirements for serious adverse events 106
8.5. Follow-up of adverse events, serious adverse events, and pregnancies 107
8.5.1. Follow-up of adverse events and serious adverse events 107
8.5.2. Follow-up of pregnancies 107
8.6. Treatment of adverse events 108
8.7. Unblinding 108
8.8. Emergency unblinding 108
8.9. Subject card 109

9. SUBJECT COMPLETION AND WITHDRAWAL 109
9.1. Subject completion 109
9.2. Subject withdrawal 109
9.2.1. Subject withdrawal from the study 109
9.2.2. Subject withdrawal from investigational vaccine 110

10. DATA EVALUATION: CRITERIA FOR EVALUATION OF OBJECTIVES 111
10.1. Primary endpoint 111
10.2. Secondary endpoints 111
10.3. Exploratory endpoints 112
10.4. Estimated sample size 112
10.5. Study cohorts to be evaluated 113
10.5.1. Total vaccinated cohort 113

10.5.2. According-to-protocol (ATP) cohort for analysis of efficacy 113
10.5.3. According-to-protocol (ATP) cohort for analysis of immunogenicity 113
10.6. Derived and transformed data 113
10.7. Conduct of analyses 114
10.7.1. Sequence of analyses 114
10.7.2. Statistical considerations for interim analyses 115
10.8. Statistical methods 115
10.8.1. Analysis of demographics/baseline characteristics 115
10.8.2. Analysis of efficacy 115
10.8.3. Analysis of immunogenicity 115
10.8.4. Analysis of safety 116

11. ADMINISTRATIVE MATTERS 117
11.1. Remote Data Entry instructions 117
11.2. Monitoring by GSK Biologicals 117
11.3. Archiving of data at study sites 118
11.4. Audits 119
11.5. Posting of information on Clinicaltrials.gov 119
11.6. Ownership, confidentiality and publication 119
11.6.1. Ownership 119
11.6.2. Confidentiality 120
11.6.3. Publication 120
11.6.4. Provision of study results to investigators, posting to the clinical trials registers and publication 120

12. COUNTRY SPECIFIC REQUIREMENTS 121

13. REFERENCES 121

LIST OF TABLES

		PAGE
Table 1	Number (%) of subjects with solicited systemic reactions, by maximal severity, after each injection	35
Table 2	Number (%) of subjects with unsolicited adverse events	35
Table 3	Study groups and epochs foreseen in the study	40
Table 4	Study groups	41
Table 5	Blinding of study epochs	41
Table 6	Sampling timepoints and volume of blood collected	42
Table 7	List of study procedures – Screening epoch (vaccinees and infectivity controls)	62
Table 8	List of study procedures - Vaccination epoch (vaccinees)	63
Table 9	List of study procedures - Challenge epoch (vaccinees and infectivity controls)	65
Table 10	List of study procedures - Follow-up epoch (vaccinees and infectivity controls)	67
Table 11	Intervals between study visits for vaccinees	67
Table 12	Intervals between study visits for infectivity controls	68
Table 13	Biological samples	76
Table 14	Humoral Immunity (Antibody determination)	77
Table 15	Cell-Mediated Immunity (CMI)	78
Table 16	Gene expression profiling as part of a systems biology approach	78
Table 17	Molecular Biology (PCR tests)	78
Table 18	Haematology, Serum Chemistry, Urine tests	79
Table 19	Urine analysis for safety	79
Table 20	Summary of blood sampling time points and laboratory assays	80
Table 21	Study vaccines	85
Table 22	Dosage and administration	87
Table 23	Solicited local adverse events	93

Table 24 Solicited general adverse events....93

Table 25 List of potential immune-mediated diseases....95

Table 26 Reporting periods for adverse events, serious adverse events and pregnancies....98

Table 27 Intensity scales for solicited symptoms....100

Table 28 Normal ranges and toxicity grading scales for blood testing....102

Table 29 Time frames for submitting SAEs, pregnancy and other events reports to GSK Biologicals, MVI and Crucell....104

Table 30 Percentages with 80 percent power to detect event rate fold increase in the ARR group....112

LIST OF APPENDICES

PAGE

Appendix A NHANES I cardiovascular risk criteria 123

Appendix B Neurologic Examination Form 126

Appendix C Laboratory assays 128

Appendix D Amendments and administrative changes to the protocol 133

LIST OF ABBREVIATIONS

**Ab**	Antibody
**Ad35.CS.01**	Crucell's candidate replication deficient adenovirus type 35 circumsporozoite malaria vaccine
**AE**	Adverse event
**ALT**	Alanine Aminotransferase
**AST**	Aspartate Aminotransferase
**ATP**	According-To-Protocol
**β-HCG**	Urine beta-human chorionic gonadotrophin
**eCRF**	electronic Case Report Form
**CBC**	Complete blood count
**CI**	Confidence Interval
**CMI**	Cell mediated immunity
**CP**	Concept protocol
**CS**	Circumsporozoite protein
**CTA**	Clinical Trial Agreement
**CVD**	Center for Vaccine Development
**ELISA**	Enzyme-linked immunosorbent assay
**EPI**	Expanded Programme on Immunization
**FDA**	Food and Drug Administration, United States
**GCP**	Good Clinical Practice
**GMT**	Geometric mean titer
**GSK**	GlaxoSmithKline
**HBsAg**	Hepatitis B surface antigen
**HIV**	Human immunodeficiency virus
**HLA**	Human Leukocyte Antigen

**ICF**	Informed Consent Form
**ICS**	Intra-cellular cytokine staining
**IEC**	Independent Ethics Committee
**IFNγ**	Interferon gamma
**IM**	Intramuscular
**IRB**	Institutional Review Board
**IND**	Investigational New Drug
**ITT**	Intent-to-treat cohort
**μg**	Microgram
**LSC**	Local Study Contact
**mL**	Millilitre
**MedDRA**	Medical Dictionary for Regulatory Activities
**MPL**	3-deacylated monophosphoryl lipid A
**MVI**	PATH Malaria Vaccine Initiative
**NHANES I**	National Health and Nutrition Examination Survey I
**NMRC**	Naval Medical Research Center
**PCR**	Polymerase Chain Reaction
**PI**	Principal Investigator
P. falciparum	*Plasmodium falciparum*
**PBMC**	Peripheral blood mononuclear cells
**QCM**	Quartz Crystal Microbalance technology Attana Cell 100 instrument
**QS-21**	Quillaja saponaria 21 (a triterpene glycoside purified from the bark of the soap bark tree, *Quillaja saponaria)*
**RAP**	Report analysis plan
**RDE**	Remote data entry

**RTS,S/AS**	GSK Biologicals' candidate *P. falciparum* malaria vaccine adjuvanted with GSK Biologicals' proprietary Adjuvant Systems
**SAE**	Serious Adverse Event
**SBIR**	Simply the Best Internet Randomization (a central randomization system on Internet)
**SMC**	Safety Monitoring Committee
**SPM**	Study Procedures Manual
**SSP**	Study Specific Procedure
**ULN**	Upper Limit of the Normal range
**VE**	Vaccine efficacy
**WRAIR**	Walter Reed Army Institute of Research
**WBC**	White Blood Cells
**WHO**	World Health Organization

GLOSSARY OF TERMS

Adequate FDA-approved contraception:	Adequate FDA-approved contraception is defined as a contraceptive method with failure rate of less than 1% per year when used consistently and correctly (when applicable, as mentioned in the product label) for example abstinence, combined or progestogen oral contraceptives, injectable progestogen, implants of levonorgestrel, oestrogenic vaginal ring, percutaneous contraceptive patches or intrauterine device or intrauterine system, vasectomy with documented azoospermia of the sole male partner or male condom combined with a vaginal spermicide (foam, gel, film, cream or suppository) or male condom combined with a female diaphragm, either with or without a vaginal spermicide (foam, gel, film, cream, or suppository).<br><br>For azoospermia, 'documented' refers to the outcome of the investigator's/designee's medical examination of the subject or review of the subject's medical history for study eligibility, as obtained via a verbal interview with the subject or from the subject's medical records.
Adverse event:	Any untoward medical occurrence in a patient or clinical investigation subject, temporally associated with the use of a medicinal product, whether or not considered related to the medicinal product.<br><br>An AE can therefore be any unfavourable and unintended sign (including an abnormal laboratory finding), symptom, or disease (new or exacerbated) temporally associated with the use of a medicinal product. For marketed medicinal products, this also includes failure to produce expected benefits (i.e. lack of efficacy), abuse or misuse.
Blinding:	A procedure in which one or more parties to the trial are kept unaware of the treatment assignment in order to reduce the risk of biased study outcomes. The level of blinding is maintained throughout the conduct of the trial, and only when the data are cleaned to an acceptable level of quality will appropriate personnel be unblinded or when required in case of a serious adverse event. In an observer-blind study, the subject and the site and sponsor personnel involved in the clinical evaluation of the subjects are blinded while other study personnel may be aware of the treatment assignment (see Section 5.3 for details on observer-blinded studies). Partially-blind is to

	be used for study designs with different blinding levels between different groups, e.g. double-blinded consistency lots which are open with respect to the control group.
Child in care:	A child who has been placed under the control or protection of an agency, organisation, institution or entity by the courts, the government or a government body, acting in accordance with powers conferred on them by law or regulation. The definition of a child in care can include a child cared for by foster parents or living in a care home or institution, provided that the arrangement falls within the definition above. The definition of a child in care does not include a child who is adopted or has an appointed legal guardian.
Eligible:	Qualified for enrolment into the study based upon strict adherence to inclusion/exclusion criteria.
Epoch:	An epoch is a well defined part of a protocol that covers a set of consecutive timepoints. Generally, an epoch is self-contained and allows to perform a data analysis to address some of the trial objectives (e.g. primary, booster, yearly follow-ups,…).
eTrack:	GSK's tracking tool for clinical trials.
Evaluable:	Meeting all eligibility criteria, complying with the procedures defined in the protocol, and, therefore, included in the according-to-protocol (ATP) analysis (see Sections 6.7.1 and 10.5 for details on criteria for evaluability).
Investigational vaccine/product:<br><br>(Synonym of Investigational Medicinal Product)	A pharmaceutical form of an active ingredient or placebo being tested or used as a reference in a clinical trial, including a product with a marketing authorisation when used in a way different from the approved form, or when used for an unapproved indication, or when used to gain further information about an approved use.
Menarche:	Menarche is the onset of menses for the first time in a young female and is preceded by several changes associated with puberty including breast development and pubic hair growth. Menarche usually occurs within 1-2 years of breast development, thelarche. However, a young female can become pregnant before her first menses. Thus, a conservative definition of non-childbearing potential in a pre-menarcheal female is a young female who has not yet entered puberty as evidenced by lack of

	breast development (palpable glandular breast tissue).
Menopause:	Menopause is the age associated with complete cessation of menstrual cycles, menses, and implies the loss of reproductive potential by ovarian failure. A practical definition accepts menopause after 1 year without menses with an appropriate clinical profile at the appropriate age e.g. > 45 years.
Protocol amendment:	The International Conference on Harmonisation (ICH) defines a protocol amendment as: 'A written description of a change(s) to or formal clarification of a protocol.' GSK Biologicals further details this to include a change to an approved protocol that affects the safety of subjects, scope of the investigation, study design, or scientific integrity of the study.
Protocol administrative change:	A protocol administrative change addresses changes to only logistical or administrative aspects of the study.<br><br>NB Any change that falls under the definition of a protocol amendment (e.g. a change that affects the safety of subjects, scope of the investigation, study design, or scientific integrity of the study) MUST be prepared as an amendment to the protocol.
Randomisation:	Process of random attribution of treatment to subjects in order to reduce bias of selection.
Site Monitor:	An individual assigned by the sponsor who is responsible for assuring proper conduct of clinical studies at one or more investigational sites.
Solicited adverse event:	Adverse events (AEs) to be recorded as endpoints in the clinical study. The presence/occurrence/intensity of these events is actively solicited from the subject or an observer during a specified post-vaccination follow-up period.
Sub-cohort:	A group of subjects for whom specific data are collected compared to other subjects.
Subject:	Term used throughout the protocol to denote an individual who has been contacted in order to participate or participates in the clinical study, either as a recipient of the product(s) or as a control.
Subject number:	A unique number identifying a subject, assigned to each subject consenting to participate in the study.

Treatment:	Term used throughout the clinical study to denote a set of investigational product(s) or marketed product(s) or placebo intended to be administered to a subject, identified by a unique number, according to the study randomisation or treatment allocation.
Treatment number:	A number identifying a treatment to a subject, according to the study randomisation or treatment allocation.
Unsolicited adverse event:	Any adverse event (AE) reported in addition to those solicited during the clinical study. Also any 'solicited' symptom with onset outside the specified period of follow-up for solicited symptoms will be reported as an unsolicited adverse event.

TRADEMARKS

The following trademarks are used in the present protocol.

Note: In the body of the protocol (including the synopsis), the names of the vaccines will be written without the superscript symbol ™ or ®.

Trademarks of the GlaxoSmithKline group of companies	Generic description
Not applicable	

Trademarks not owned by the GlaxoSmithKline group of companies	Generic description
Not applicable	

1. INTRODUCTION

1.1. Background

The development of a malaria vaccine has been identified as a key component of future integrated malaria control programs and an important step towards sustainable elimination of malaria. Improved control of malaria would have significant benefits in global health.

GlaxoSmithKline (GSK) Biologicals is developing a pre-erythrocytic *Plasmodium falciparum* (*P falciparum)* malaria vaccine (RTS,S/AS01 vaccine) for routine immunization of infants and children living in malaria-endemic areas as part of the Expanded Program of Immunization (EPI). Refer to section 1.1.1 for further details on RTS,S/AS01 vaccine.

Although the implementation of RTS,S/AS01 vaccine in immunization programs would lead to a substantial reduction of malaria burden, the available data suggest that further improvements in vaccine efficacy levels may lead to further benefits in malaria control.

Extensive pre-clinical studies demonstrated the complementary role of anti-CS humoral and T-cell mediated immune responses, including CTLs and IFN-gamma (IFN-γ)-secreting Th1 lymphocytes responses, in the ability to protect against malaria. In humans, an association between anti-CS antibody levels as well as, independently, activated CD4+ T cells with vaccine-induced protection against infection in the challenge model has been demonstrated [Kester, 2009]. One way of increasing the level of efficacy provided by a CS-targeted vaccination regimen would be to increase the level of immunogenicity and expand the magnitude, breadth and functionality characteristics of the anti-CS immune response.

Heterologous prime-boost strategies involving the sequential administration of two or more vaccines using the same antigen of interest, whether it is encoded in plasmid DNA, encoded in recombinant viral vectors or as purified protein have been shown to increase the magnitude and/or breadth of the vaccine-specific immune responses [Hu, 1992; Lubeck, 1997; Rodríguez, 2008; Radosević, 2009; Lu, 2009]. Within the field of pre-erythrocytic malaria vaccine candidates, although an enhanced immunogenicity of heterologous prime-boost strategies involving DNA, modified vaccinia Ankara (MVA) and/or fowlpoxvirus 9 (FP9) have been reported, these have failed to elicit significant levels of protection in clinical studies in malaria endemic regions [Radosević, 2009; Hill, 2010]. The reasons for these failures are unknown but it is hypothesized that some of these so called “T-cell inducing” vaccine candidates are not sufficiently immunogenic and do not elicit potent T cell responses. In addition, whether alone or in prime-boost, these T cell inducing vaccine strategies do not likely induce sufficient Ag-specific antibodies which represent an efficient arm of the anti-malaria pre-erythrocytic protective immune response. Altogether, this suggests that heterologous prime-boost strategies should be selected on their ability to induce both potent Ag-specific T cell and antibody responses. Therefore, the sequential immunization with RTS,S/AS01 (potent inducer of CS-specific antibody and CD4 T cell responses) and a vaccine candidate that elicits strong CS-specific T cell responses would represent an ideal strategy to increase

RTS,S/AS01 vaccine efficacy. In particular, we feel that recombinant adenoviral vectors could be used as potent T cell inducer.

Crucell has generated a CS-expressing recombinant human adenovirus 35 (Ad35.CS.01) aimed at protecting vaccinated subjects against malaria through the generation of strong T-cell mediated anti-CS immune responses. Refer to Section 1.1.2 for further details on Ad35.CS.01 vaccine.

GSK Biologicals in collaboration with MVI and Crucell is developing an Ad35.CS.01 prime-RTS,S/AS01$_B$ boost immunization regimen for the prevention of malaria due to *P. falciparum* in infants and children living in malaria endemic areas. The indication of the immunization regimen may, depending on the risk/benefit profile, be extended to a wider age group.

For an extensive review of the Ad35.CS.01 preclinical and clinical experience, refer to the Ad35.CS.01 Investigator Brochure. For an extensive review of the RTS,S/AS01$_B$ preclinical and clinical experience, refer to the RTS,S/AS01$_B$ Investigator Brochure. For a review of the experience with the Ad35.CS.01 prime - RTS,S/AS01$_B$ boost immunization regimen refer to the Ad35.CS.01 prime- RTS,S/AS01$_B$ boost Investigator Brochure.

1.1.1. RTS,S candidate vaccine

The approach of GSK Biologicals' in designing the RTS,S/AS01 vaccine has been to target the free sporozoite and intra-hepatic stages of the parasites (i.e. the pre-erythrocytic stage). The target antigen is the circumsporozoite protein (CS), a 412 amino acid protein abundantly associated with the sporozoite surface, also expressed by liver forms and exported in the cytoplasm of hepatocytes. The hypothesized mode of action of a CS-targeted vaccine is to both reduce the sporozoites load reaching the liver through the intervention of antibodies and due to the short life span of free sporozoites, to promote the destruction of infected liver cells or to impede further intracellular parasite development through the action of T cells. This would lead to a significant decrease in the load of parasites emerging from the liver with a subsequent impact on disease rates and severity.

The RTS,S/AS01 candidate malaria vaccine consists of the recombinant protein RTS,S, which is comprised of sequences of the circumsporozoite (CS) protein and hepatitis B surface antigen (HBsAg), with the proprietary adjuvant AS01 (proprietary liposomes, MPL and Stimulon QS21 immunostimulants). The vaccine also induces a strong immune response against hepatitis B. In studies completed to date, the RTS,S candidate malaria vaccine adjuvanted with GSK's proprietary Adjuvant Systems have consistently demonstrated efficacy in the pediatric population in malaria endemic countries. It has a favorable safety profile when given to young children and infants in co-administration with EPI routine vaccines.

1.1.2. CS-expressing recombinant human adenovirus 35 vaccine

Adenoviruses, one of the common causes of upper respiratory and gastrointestinal infections, have been extensively studied in clinical trials and were one of the first gene

therapy vectors. Recombinant adenoviruses disabled by deletion of the early E1 genes are replication-deficient. As adenoviruses can directly infect dendritic cells, they promise to provide robust antibody and T cell responses to heterologous inserted and expressed genes.

Recombinant human adenovirus vaccine vectors, especially those using adenovirus serotype 5 (Ad5), have shown in pre-clinical and clinical Phase I studies to elicit potent antigen-specific cellular immune responses [Graham, 2005; Catanzaro, 2006; Harro, 2009]. However, the high prevalence of pre-existing anti-Ad5 immunity in human populations, particularly in the developing world, is likely to reduce the immune response of a recombinant Ad5 vaccine [Vogels, 2003; Kostense, 2004]. Other adenovirus serotypes such as Ad35, associated with lower worldwide seroprevalence, are therefore likely to be more relevant for use as vaccine candidates for the developing world.

Crucell has generated a CS-expressing recombinant human adenovirus 35 (Ad35.CS.01) which has already been evaluated in pre-clinical studies in mice and non-human primates as a stand alone or in a homologous prime-boost regimen, and showed the capability of inducing CS-specific antibodies as well as potent T-cell responses [Stewart, 2007; Shott, 2008; Rodríguez, 2008]. The Ad35 vaccine vector produced in the E1-complementing PER.C6®cell line has E1 and E3 regions deleted and is replication-incompetent. Ad35.CS.01 incorporates the full length CS gene. The N-terminus region has a consensus sequence designed to cover various circulating polymorphisms. The C-terminus sequence resembles that in RTS,S (3D7-derived). There are 27 NANP repeats, a cluster of 3 NANPNVDP repeats, and 1 separate NVDP repeat into the CS protein. An Ad35.CS.01 prime- RTS,S/AS01 boost regimen in a preclinical non-human primate study showed enhanced immunogenicity when compared to either vaccine candidate given alone. In particular, the prime-boost regimen led to a significant enhancement of the vaccine specific CD4 T cell responses when compared to the 3x RTS,S/AS01 immunization regimen [Stewart, 2007]. This Ad35.CS.01 vaccine candidate from Crucell has recently been tested in a Phase I clinical study in the U.S.

Preliminary clinical data with Ad35.CS.01 are available from a randomized, double-blind Phase I study carried out by the US National Institute of Allergy and Infectious Diseases, which evaluated the safety, tolerability and immunogenicity of increasing doses (10^8 to 10^{11} vp) of Ad35.CS.01. A total of 60 subjects (14, 15, 15 and 16 at the 10^8, 10^9, 10^{10}, and 10^{11} vp dose level, respectively) received vaccine, 12 subjects received a saline placebo at 0, 1, and 6 months.

The safety and tolerability of the Ad35.CS.01 malaria vaccine was evaluated through the assessment of local and systemic solicited reactions, unsolicited adverse events and clinical laboratory parameters.

The local tolerability of the vaccine was acceptable at all doses. The 10^{11} vp dose was more frequently associated with pain of mild to moderate severity than the lower doses. The frequency of erythema was dose-dependent as well, but all the events were consistently mild across all doses. All solicited local reactions resolved without sequelae.

Systemic tolerability was assessed through the following solicited symptoms and signs: headache, malaise, myalgia, chill, nausea, vomiting and fever. Symptoms were scored

daily by subjects on diaries for 14 days after each dose. Results are summarized in Table 1.

Table 1 Number (%) of subjects with solicited systemic reactions, by maximal severity, after each injection

		Placebo (N=12)	Ad35.CS.01 10^8 (N= 14)	Ad35.CS.01 10^9 (N=15)	Ad35.CS.01 10^{10} (N=15)	Ad35.CS.01 10^{11} (N=16)
First dose	Mild	1 (8)	3 (21)	2 (13)	4 (27)	6 (38)
	Moderate	2 (17)	2 (14)	0 (0)	1 (7)	5 (31)
	Severe	0 (0)	0 (0)	0 (0)	1 (7)	2 (13)
		**N=12**	**N=14**	**N=13**	**N=15**	**N=14**
Second dose	Mild	4 (33)	2 (14)	3 (23)	4 (27)	7 (50)
	Moderate	1 (8)	2 (14)	3 (23)	4 (27)	4 (29)
	Severe	0 (0)	1 (7)	0 (0)	0 (0)	1 (7)
		**N=12**	**N=12**	**N=13**	**N=14**	**N=12**
Third dose	Mild	2 (17)	6 (50)	1 (8)	1 (7)	6 (50)
	Moderate	0 (0)	2 (17)	1 (8)	5 (36)	3 (25)
	Severe	0 (0)	0 (0)	0 (0)	0 (0)	1 (8)
Any dose	Mild	5 (42)	5 (36)	4 (27)	4 (27)	4 (25)
	Moderate	2 (17)	4 (29)	4 (27)	6 (40)	7 (44)
	Severe	0 (0)	1 (7)	0 (0)	1 (7)	4 (25)

Systemic reactogenicity following the 3 lower doses was comparable to (10^8 and 10^9 vp) or only slightly higher (10^{10} vp) than that observed after placebo, while the dose of 10^{11} vp induced more frequently moderate to severe systemic reactions, in 2 cases leading to treatment discontinuation after the first dose.

The number and the percentage of subjects with unsolicited AEs (assessed by neutral questioning) ranged from 87% (Ad35.CS.01 10^{11} vp) to 100% (Ad35.CS.01 10^9vp and 10^{10}vp). The incidence rate was comparable to placebo (92%) (see Table 2).

Table 2 Number (%) of subjects with unsolicited adverse events

Subjects with	Placebo (N = 12)	Ad35.CS.01 10^8vp (N = 14)	Ad35.CS.01 10^9 vp (N = 15)	Ad35.CS.01 10^{10} vp (N = 15)	Ad35.CS.01 10^{11} vp (N = 16)
AEs	11 (92)	13 (93)	15 (100)	15 (100)	14 (87)
AEs related to IMP	4 (33)	3 (21)	9 (60)	7 (47)	7 (44)
AEs leading to discontinuation	0 (0)	0 (0)	0 (0)	0 (0)	2 (13)
SAEs	1 (8)	0 (0)	0 (0)	0 (0)	0 (0)

Percentages are based on N (number of subjects dosed in the treatment group). AE = adverse event; IMP = investigational medicinal product (Ad35.CS.01 or placebo); SAE = serious AE

The analysis of the AEs did not show any consistent pattern that might suggest an association of Ad35.CS.01 with specific AEs. The AEs judged to be related to Ad35.CS.01 were mostly mild and moderate in severity.

The only SAE reported in the study (a small bowel obstruction requiring hospitalization and surgery) was reported in a subject receiving placebo.

Unsolicited AEs leading to study discontinuation were reported by 2 subjects in the 10^{11} vp dose group: one subject complained of 2 episodes of weakness in the vaccinated arm after the first and second vaccination, respectively, noticed on the first instance during recreational weightlifting. He also reported a transient lack of balance on return from a boat trip; one subject reported pain in the forearm, classified as a brachial plexopathy of moderate severity after dose 2. He was a guitar player who had experienced similar symptoms in the past. Both subjects were withdrawn from the study prior to the administration of the third vaccination.

Symptoms in the vaccinated arms were reported by other two subjects who completed the study, one at the dose of 10^8 vp and one at the dose of 10^{11} vp. The former subject (10^8 vp dose) complained of paresthesias of moderate severity in the vaccinated arm after the second and third dose of vaccine. The symptoms resolved without sequelae in 1 day in both cases. The subject had a history of a similar epidose occurred years before. The second subject complained of a mild sensation of heaviness in the elbow of the vaccinated arm after the first dose of the vaccine at the dose of 10^{11} vp. The symptoms resolved without sequelae within 48 hours, and did not recur following the second and third dose of the vaccine.

In summary, symptoms that might be referred to as peripheral nervous impairment in the vaccinated arm were reported by 4 subjects: the symptoms were mild to moderate, resolved without sequelae in all cases, within 1 to 3 days in all subjects.

Blood laboratory test abnormalities were recorded with a similar frequency in the vaccinated and placebo groups, without suggesting any relationship with Ad35.CS.01.

Urinalysis findings showed more frequently positive blood urine tests in the Ad35.CS.01 groups than in the placebo group; this finding, which will be closely monitored in future studies, may be related to the unbalanced distribution of female subjects in the vaccine groups (47 to 60%) compared to placebo group (33%). All subjects with hematuria were in fact females with menses at the time of urine collection.

Immunogenicity was evaluated through assessment of both humoral immune response (anti-CS antibody levels) and cellular immune responses. Humoral responses against the vector were assessed as well by Ad35 neutralization assay.

Anti-CS antibodies were assessed at baseline, 1 month after each dose of vaccine (month 1, 2 and 7) and at month 6 (5 months after the second dose and prior to the last dose of vaccine) with an ELISA assay using a $(NANP)_6$ peptide as capture antigen. The two lowest doses induced poor or no humoral responses. Anti-CS antibody levels following the 10^8 vp dose were not distinguishable from those recorded in the placebo group, while in the 10^9 vp dose approximately 30% of the subjects showed a ≥4 fold increase vs. baseline in anti-CS antibodies at any time during the study. On the contrary, in the remaining 2 groups, anti-CS antibodies levels showed a dose dependent increase, with 53% and 79% of subjects showing a ≥4 fold increase vs. baseline in the 10^{10} and 10^{11} vp dose group, respectively, 1 month after the second dose of vaccine. The immune response was further boosted by the third dose, particularly in the 10^{11} vp dose group.

Cellular immune responses, evaluated through an IFN-γ ELISPOT, are more difficult to interpret due to the small sample sizes, and sub-optimal collection and processing of PBMC's. However, the dose of 10^{11} vp consistently provided some degree of cellular response at all time points evaluated.

Anti-Ad35 antibodies were elicited in a minority of subjects. The responses showed some level of dose-dependency, with no sign of interference with the anti-CS immunogenicity.

In conclusion, the administration of Ad35.CS.01 induces an antibody response against the CS protein in a dose dependent way. Priming is successfully achieved in most of the subjects receiving the doses of 10^{10} and 10^{11} vp, with higher titers observed in the 10^{11} vp dose group. Boosting with further dose(s) of Ad35.CS.01 has proven to be possible in this study, particularly at the higher dose. Additional studies are warranted to assess whether other boosting modalities can be used to achieve higher humoral responses.

1.2. Rationale for the study and study design

1.2.1. Rationale for the study

GSK Biologicals in collaboration with MVI and Crucell is developing an Ad35.CS.01 prime-RTS,S/AS01$_B$ boost immunization regimen for prevention of malaria due to *P. falciparum* in infants and children living in malaria endemic areas.

While both RTS,S/AS01$_B$ and Ad35.CS.01 candidate malaria vaccines tested in this study have been administered to humans before, this trial will be the first one where they are combined in one immunization schedule.

1.2.2. Rationale for the study design

This study is designed to evaluate safety, reactogenicity, immunogenicity and efficacy of Crucell's candidate replication deficient adenovirus type 35 circumsporozoite malaria vaccine (Ad35.CS.01) administered intramuscularly followed one month later by two doses of GSK Biological's malaria candidate vaccine RTS,S/AS01$_B$ administered intramuscularly at one month intervals, compared to three doses of RTS,S/AS01$_B$ administered intramuscularly at one month intervals in healthy malaria-naïve volunteers aged 18 to 50 years. The sporozoite challenge model, in the RTS,S/AS candidate vaccine development program, has demonstrated a high relevance in its ability to predict efficacy under conditions of natural exposure in malaria-endemic countries. Approximately 168 participants will be enrolled in the study. Three cohorts of approximately equal size will be enrolled, vaccinated then challenged separately. This design takes into account logistical restrictions on the size of human challenge procedures and allows for a futility analysis to be conducted after the results of the malaria challenge in the first cohort. The proposed study design is such that the sample size will provide 80% power to demonstrate a 50% increase in efficacy of an immunization regimen including an initial dose of Ad35.CS.01 over efficacy of a regimen based on RTS,S/AS01$_B$ alone, assuming 50% vaccine efficacy of the RTS,S/AS01$_B$ group over challenge infectivity controls.

The study design is similar to that of other past studies successfully conducted. Healthy adults will be enrolled and followed up in closely controlled conditions by investigators

experienced in *P. falciparum* challenge studies. Immunological investigations are planned with the intent to characterize qualitatively and quantitatively the immune response induced by the immunization regimen and assess the presence of a correlation of specific effector immune function(s) with protection against malaria infection in the sporozoite challenge model. Protection will be evaluated by the proportion of immunized participants who remain free of *P. falciparum* infection following sporozoite challenge and by a delay in the pre-patent period leading to infection.

2. OBJECTIVES

2.1. Primary objective

- To compare the efficacy (occurrence of *P. falciparum* parasitemia, assessed by blood slide) of an immunization regimen comprising of one dose of Ad35.CS.01 followed one month later by two doses of RTS,S/$AS01_B$ administered at one month intervals, with that of three doses of RTS,S/$AS01_B$ administered at one month intervals, in healthy malaria-naïve volunteers aged 18-50 years in the sporozoite challenge model.
- To assess the safety (reactogenicity, unsolicited adverse events, SAEs up to 30 days post last vaccination [day of vaccination and 29 subsequent days], SAEs up to study conclusion) of an immunization regimen comprising of one dose of Ad35.CS.01 followed one month later by two doses of RTS,S/$AS01_B$ administered at one month intervals in healthy malaria-naïve volunteers aged 18-50 years.

Refer to Section 10.1 for the definition of the primary endpoints.

2.2. Secondary objectives

- To compare the efficacy (time to *P. falciparum* parasitemia, assessed by blood slide) of an immunization regimen comprising of one dose of Ad35.CS.01 followed one month later by two doses of RTS,S/$AS01_B$ administered at one month intervals, with that of three doses of RTS,S/$AS01_B$ administered at one month intervals, in healthy malaria-naïve volunteers aged 18-50 years in the sporozoite challenge model.
- To assess the humoral and cell-mediated immune response to malaria and hepatitis B antigens (by ELISA, ICS and ELISPOT) induced by one dose of Ad35.CS.01 followed one month later by two doses of RTS,S/$AS01_B$ administered at one month intervals in healthy malaria-naïve volunteers aged 18-50 years.
- To assess the humoral immune response to adenovirus antigen induced by one dose of Ad35.CS.01 followed one month later by two doses of RTS,S/$AS01_B$ administered at one month intervals in healthy malaria-naïve volunteers aged 18-50 years.

Refer to Section 10.2 for the definition of the secondary endpoints.

2.3. Exploratory objectives

- To compare the efficacy (occurrence of *P. falciparum* parasitemia and time to *P. falciparum* parasitemia, assessed by PCR) of an immunization regimen comprising of one dose of Ad35.CS.01 followed one month later by two doses of RTS,S/$AS01_B$

administered at one month intervals, with that of three doses of RTS,S/AS01$_B$ administered at one month intervals, in healthy malaria-naïve volunteers aged 18-50 years in the sporozoite challenge model.

- To assess the immune response to malaria, hepatitis B and adenovirus antigens (by various methods including high-throughput technologies such as microarray analysis and deep sequencing, as part of a systems biology approach) induced by one dose of Ad35.CS.01 followed one month later by two doses of RTS,S/AS01$_B$ administered at one month intervals in healthy malaria-naïve volunteers aged 18-50 years.

Refer to Section 10.3 for the definition of the exploratory endpoints.

3. STUDY DESIGN OVERVIEW

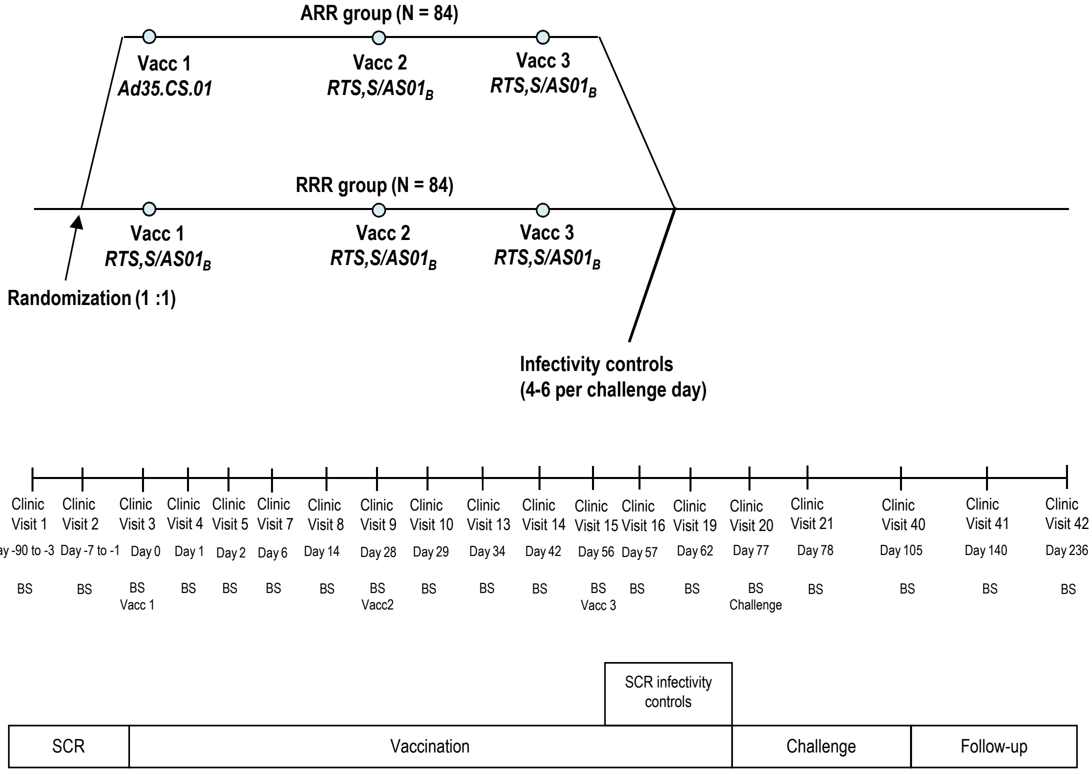

Vacc = vaccination; **SCR** = screening; **BS** = Blood sampling; **RRR** group = RTS,S/AS01$_B$ group; **ARR** group = Ad35.CS.01 + RTS,S/AS01$_B$ group

- Experimental design: Phase I/II a, observed-blind, randomised, controlled, multi-centric, single-country study with two parallel groups and infectivity controls.
- Duration of the study: For each vaccinated subject, the study duration will be approximately 11 months. For each infectivity control subject, the study duration will be approximately 8 months.
  - Screening epoch: Screening (and enrollment) starting Visit 1 (Day -90 to -3) and ending Visit 2 (Day -7 to -1).
  - Vaccination epoch: Vaccination starting Visit 3 (Day 0) and ending Visit 19 (Day 62).

- Challenge epoch: Challenge starting Visit 20 (Day 77) and ending Visit 40 (Day 105).
- Follow-up epoch: Follow-up starting Visit 41 (Day 140) and ending Visit 42 (Day 236).

Table 3 Study groups and epochs foreseen in the study

Study groups	Number of subjects	Age (Min/Max)	Epochs			
			Screening epoch	Vaccination epoch	Challenge epoch	Follow-up epoch
RRR	84	18 - 50 years	x	x	x	x
ARR	84	18 - 50 years	x	x	x	x
Infectivity controls	4 - 6 per challenge day	18 - 50 years	x		x	x

ARR = first dose with Ad35.CS.01, second and third doses with RTS,S/$AS01_B$
RRR = three doses of RTS,S/$AS01_B$

- Control: active control. The group receiving three doses of RTS,S/$AS01_B$ will be the comparator group. In addition, there will be infectivity controls for the challenge epoch (i.e. volunteers who will not receive any immunization but will be subjected to the sporozoite challenge).
- Vaccination schedule: 0-1-2 months followed by a challenge 21 days (3 weeks) ± 7 days after the third vaccination.
- Safety considerations:

  Vaccination and challenge will be performed in a staggered fashion. Three cohorts (Cohort A, B and C) of approximately equal size will be enrolled, vaccinated and challenged separately. Each cohort which enters the vaccination phase will include approximately 56 subjects. Among the 56 vaccinated individuals a random selection of subjects eligible for challenge will be done according to the local SOP to ensure that a maximum of 46 vaccinated individuals (balanced by group) will progress to the challenge phase. The remaining subjects will not be challenged but they will be followed for safety and immunogenicity up to 6 months after the last vaccination (Study Day 236). Vaccination and challenge will be performed first in Cohort A. Safety and efficacy data post-challenge from this initial cohort and 4 to 6 infectivity controls will be reviewed prior to the start of vaccination in Cohorts B and C, based on pre-defined group holding criteria for safety review (see Section 5.4.4.1) and pre-defined criteria for efficacy futility analysis (see Section 5.4.4.2). Vaccination and challenge of subjects in Cohorts B and C is likely to take place in parallel at two different sites (see Section 4.2 for more details).

  In addition, the Principal Investigator (PI) will have the responsibility to withdraw from further vaccination individual study participants who present at least one of the pre-defined stopping rules for individual subjects (see Section 5.4.4.1).
- Safety and immunogenicity will be evaluated during the study through 6 months after the last vaccination (Study Day 236).
- Study groups: refer to Table 4.

Table 4 Study groups

Treatment name	Vaccine/Product name	Study groups		
		RRR	ARR	Infectivity controls
Ad35.CS.01	Ad35.CS.01		x	
RTS,S/AS01B	RTS,S	x	x	
	AS01B	x	x	

- Treatment allocation: randomised (1:1).
- Blinding: observer-blind as to immunization regimens (see Table 5).

Table 5 Blinding of study epochs

Study Epochs	Study groups	Blinding
Screening epoch	Immunization groups	N/A
	Infectivity controls	N/A
Vaccination epoch	Immunization groups	observer-blind
	Infectivity controls	N/A
Challenge epoch	Immunization groups	observer-blind
	Infectivity controls	open
Follow-up epoch	Immunization groups	open
	Infectivity controls	open

N/A: Not applicable

- Blood sampling: refer to Table 6.

Table 6 Sampling timepoints and volume of blood collected

**Epoch**	**Screening**		**Vaccination**											
**Study Day**	**-90 to -3**	**-7 to-1**	**0**	**1**	**2**	**6**	**14**	**28**	**29**	**34**	**42**	**56**	**57**	**62**
**Visit number**	**1**	**2**	**3**	**4**	**5**	**7**	**8**	**9**	**10**	**13**	**14**	**15**	**16**	**19**
Safety labs (7ml)*	7		7			7		7		7		7		7
Additional screening labs (15 ml)**	15													
Humoral response (20 ml)		20						20				20		
Cell-mediated immunity assays (60 ml)		60					60				60			
Exploratory cytokine/chemokine analysis (Luminex) (2 ml)		2		2	2	2	2	2	2	2		2	2	2
Microarray and deep sequencing analysis (12 ml)		12		12	12	12	12	12	12	12		12	12	12
Plasmablasts FACS and B cell ELISPOT (10 ml)		10						10		10		10		10
**Blood volume in mL per visit**	**22**	**104**	**7**	**14**	**14**	**21**	**74**	**51**	**14**	**31**	**60**	**51**	**14**	**31**
Cumulative blood vol. - Immunized groups (ml)	22	126	133	147	161	182	256	307	321	352	412	463	477	508
Cumulative blood vol. – Infectivity controls (ml)	22	-	-	-	-	-	-	-	-	-		-	-	-

*Safety labs include hemoglobin, WBC, PLT, ALT, AST, creatinine. **Additional screening labs include HIV, HCV, HBV.

**Epoch**	**Challenge**																					**Follow-up**	
	**DoC***	**Early Post-challenge**					**Hotel/inpatient Phase**										**Late Post-challenge**					**Final Visits**	
**Study day**	**77**	**78**	**82**	**83**	**84**	**85**	**86**	**87**	**88**	**89**	**90**	**91**	**92**	**93**	**94**	**95**	**97**	**99**	**101**	**103**	**105**	**140**	**236**
**Challenge day**	**0**	**1**	**5**	**6**	**7**	**8**	**9**	**10**	**11**	**12**	**13**	**14**	**15**	**16**	**17**	**18**	**20**	**22**	**24**	**26**	**28**	**63**	**159**
**Visit number**	**20**	**21**	**22**	**23**	**24**	**25**	**26**	**27**	**28**	**29**	**30**	**31**	**32**	**33**	**34**	**35**	**36**	**37**	**38**	**39**	**40**	**41**	**42**
Safety labs (7 ml)**	7													7***							7	7	7
Smear and PCR (2 ml)			2	2	2	2	2	2	2	2	2	2	2	2	2	2	2‡	2‡	2‡	2‡	2‡		
Humoral immunity (ELISA) (20 ml)	20																				20	20	20
Cellular immunity (60 mL)	60																				60	60	60
Exploratory cytokine/chemokine analysis (Luminex) (2ml)	2	2	2																		2		
Microarray and deep sequencing analysis (12ml)	12	12	12																				
**Blood volume in ml per visit**	**101**	**14**	**16**	**2**	**2**	**2**	**2**	**2**	**2**	**2**	**2**	**2**	**2**	**9****	**2**	**2**	**2**	**2**	**2**	**2**	**91**	**87**	**87**
Cumulative blood vol. - Immunized groups (ml)	609	623	639	641	643	645	647	649	651	653	655	657	659	668	670	672	674	676	678	680	771	858	945
Cumulative blood vol. – Infectivity controls (ml)	123	137	153	155	157	159	161	163	165	167	169	171	173	182	184	186	188	190	192	194	285	372	459

*DoC: day of challenge. **Safety labs include hemoglobin, WBC, PLT, ALT, AST, creatinine. ***Day of parasitemia and number of parasitemic individuals is undetermined.

‡ For volunteers who develop malaria: blood smears and PCR may be discontinued once the volunteer has 3 consecutive (separated by greater than 12 hours) negative smears following initial treatment. **(Amended 09 June 2011)**

- Type of study: self-contained.
- Data collection: Electronic Case Report Form (eCRF).

4. STUDY COHORT

4.1. Number of subjects/centers

The target enrollment for this study will be 168 healthy malaria-naïve adult subjects (84 subjects per group), to ensure 138 subjects will undergo sporozoite challenge (69 subjects per group), given an approximate 20% estimated drop-out rate, based on past experience. Additional volunteers to serve as infectivity controls will be enrolled. Between four to six control volunteers (not immunized) will be enrolled for each sporozoite challenge day.

Refer to Section 10.4 for a detailed description of the criteria used in the estimation of sample size.

4.2. Overview of the recruitment plan

- This will be a multicenter study to be conducted in the United States of America (USA).
- Recruitment will be conducted using multiple methods such as advertisements, flyers, posters, word of mouth and other IRB approved methods. There will be an opportunity for volunteers who enroll in the study to receive compensation for recruitment of additional volunteers.
- The study will include three cohorts (Cohort A, B and C) of approximately equal size. First, recruitment of subjects in Cohort A will take place at WRAIR. Subsequent recruitment of subjects in Cohorts B and C is likely to take place in parallel at WRAIR and the University of Maryland, Center for Vaccine Development (CVD).
- The recruitment period will be approximately 3 months per cohort.
- Recruitment (as defined by subjects receiving at least one immunization) will be terminated when 168 subjects have been recruited.

4.3. Inclusion criteria for enrollment

All subjects must satisfy ALL the following criteria at study entry:

- Subjects who the investigator believes can and will comply with the requirements of the protocol (e.g. completion of the diary cards, return for follow-up visits).
- A male or non-pregnant female 18 to 50 years of age (inclusive) at the time of first vaccination.
- Written informed consent obtained from the subject before screening procedures.
- Free of obvious health problems as established by medical history and clinical examination before entering into the study.

- Available to participate for the duration of the study (approximately 11 months per vaccinated subject or approximately 8 months per infectivity control).
- Female subjects of non-childbearing potential may be enrolled in the study.
  - Non-childbearing potential is defined as pre-menarche, current tubal ligation, hysterectomy, ovariectomy or post-menopause.

  Please refer to the glossary of terms for the definition of menarche and menopause.
- Female subjects of childbearing potential may be enrolled in the study, if the subject:
  - has practiced adequate FDA-approved contraception for 30 days prior to vaccination, and
  - has a negative pregnancy test on the day of vaccination, and
  - has agreed to continue adequate FDA-approved contraception during the entire treatment period and for 2 months after completion of the vaccination series and/or malaria challenge.

  Please refer to the glossary of terms for the definition of adequate FDA-approved contraception.
- Prior to entry into this study, subjects must score at least 80% correct on a short multiple-choice quiz that assesses their understanding of this study. If they do not score 80% on the initial quiz, the protocol information will be reviewed with them to ensure comprehension and they will have the opportunity to retest. Subjects who fail the Comprehension Assessment for the second time will not be enrolled.

4.4. Exclusion criteria for enrollment

The following criteria should be checked at the time of study entry. If ANY exclusion criterion applies, the subject must not be included in the study:

- Use of any investigational or non-registered product (drug or vaccine) within 30 days preceding the first dose of study vaccine, or planned use of any investigational or non-registered product (drug or vaccine) other than the study vaccines during the study period.
- Planned administration/ administration of a vaccine not foreseen by the study protocol within 7 days of the first dose of vaccines.
- Prior receipt of an investigational malaria or adenovirus vaccine.
- Chronic use of antibiotics with antimalarial effects (e.g., tetracyclines for dermatologic patients, sulfa for recurrent urinary tract infections, etc.).
- History of malaria chemoprophylaxis within 60 days prior to vaccination.
- Any history of malaria.
- Planned travel to malaria endemic areas during the study period.
- History of allergic disease or reactions likely to be exacerbated by any component of the vaccine(s) including latex.

- History of allergic disease or reactions likely to be exacerbated by chloroquine.
- History of psoriasis and porphyria, which may be exacerbated after chloroquine treatment.
- Current use of medications known to cause drug reactions to chloroquine, such as antacids and kaolin.
- Any history of anaphylaxis in reaction to any previous vaccination.
- History of severe reactions to mosquito bites.
- Administration of immunoglobulins and/or any blood products within the three months preceding the first dose of study vaccine or planned administration during the study period.
- Chronic administration (defined as more than 14 days) of immunosuppressants or other immune modifying drugs within six months prior to first vaccine dose. For corticosteroids, this will mean prednisone, or equivalent, greater than or equal to 20 mg /day. Inhaled and topical steroids are allowed.
- Any confirmed or suspected immunosuppressive or immunodeficient condition, including immunodeficiency virus (HIV) infection.
- Family history of congenital or hereditary immunodeficiency.
- History of splenectomy.
- Major congenital defects or serious chronic illness.
- History of any neurological disorders or seizures.
- Acute disease and/or fever at the time of enrollment.
  - Acute disease is defined as the presence of a moderate or severe illness with or without fever. Subjects with a minor illness (such as mild diarrhoea, mild upper respiratory infection) without fever may be enrolled at the discretion of the investigator.
  - Fever is defined as temperature ≥ 38.0°C (100.4°F) on oral, axillary or tympanic setting. The preferred route for recording temperature in this study will be oral.
- Acute or chronic, clinically significant pulmonary, cardiovascular, hepatic or renal functional abnormality, as determined by physical examination or laboratory screening tests.
- Any abnormal baseline laboratory screening tests: ALT, AST, creatinine, hemoglobin, platelet count, total white blood cell count, out of normal range as defined in the protocol ***Table 28***. **(Amended 09 June 2011)**
- Evidence of increased cardiovascular disease risk, "moderate" or "high", according to the NHANES I criteria (see Appendix A).

  Note: NHANES I criteria will be applied for all subjects including subjects aged 18-35 years old. **(Amended 09 June 2011)**

- An abnormal baseline screening electrocardiogram (EKG), defined as one showing pathologic Q waves and significant ST-T wave changes; left ventricular hypertrophy; any non-sinus rhythm excluding isolated premature atrial contractions; right or left bundle branch block; or advanced (secondary or tertiary) A-V heart block.
- Hepatomegaly, right upper quadrant abdominal pain or tenderness.
- Personal history of autoimmune disease.
- Seropositive for hepatitis B surface antigen or Hepatitis C virus (antibodies to HCV).
- Pregnant or lactating female.
- Female who intends to become pregnant during the study or planning to discontinue contraceptive measures.
- Suspected or known current alcohol abuse.
- Chronic or active intravenous drug use.
- History of blood donation within 56 days preceding enrolment.
- Any other significant finding that in the opinion of the investigator would increase the risk of having an adverse outcome from participating in this study.

A list of criteria that may eliminate subjects from ATP analyses can be found in Sections 6.7.1, 6.8 and 10.5.

5. CONDUCT OF THE STUDY

5.1. Regulatory and ethical considerations, including the informed consent process

The study will be conducted in accordance with all applicable regulatory requirements.

The study will be conducted in accordance with the ICH Guideline for Good Clinical Practice (GCP) all applicable subject privacy requirements and the guiding principles of the Declaration of Helsinki.

GSK will obtain favourable opinion/approval to conduct the study from the appropriate regulatory agency, in accordance with applicable regulatory requirements, prior to a site initiating the study in that country.

Conduct of the study includes, but is not limited to, the following:

- Institutional Review Board (IRB)/Independent Ethics Committee (IEC) review and favourable opinion/approval of study protocol and any subsequent amendments.
- Subject informed consent.
- Investigator reporting requirements as stated in the protocol.

GSK will provide full details of the above procedures to the investigator, either verbally, in writing, or both.

Freely given and written informed consent must be obtained from each subject prior to participation in the study.

GSK Biologicals will prepare a model Informed Consent Form (ICF) which will embody the ICH GCP and GSK Biologicals required elements. While it is strongly recommended that this model ICF is to be followed as closely as possible, the informed consent requirements given in this document are not intended to pre-empt any local regulations which require additional information to be disclosed for informed consent to be legally effective. Clinical judgement, local regulations and requirements should guide the final structure and content of the local version of the ICF.

The investigator has the final responsibility for the final presentation of the ICF, respecting the mandatory requirements of local regulations. The ICF generated by the investigator with the assistance of the sponsor's representative must be acceptable to GSK Biologicals and be approved (along with the protocol, and any other necessary documentation) by the IRB/IEC.

5.2. Subject identification and randomization of treatment

5.2.1. Subject identification

Subject numbers will be assigned sequentially to subjects consenting to participate in the study, according to the range of subject numbers allocated to each study center.

5.2.2. Randomization of treatment

5.2.2.1. Randomization of supplies

The randomization will be performed at GSK Biologicals, Rixensart, using MATEX, a program developed for use in SAS® (Cary, NC, USA) by GSK Biologicals.

To allow GSK Biologicals to take advantage of greater rates of recruitment than anticipated at individual centers in this multicenter study, and to thus reduce the overall study recruitment period, an over-randomization of supplies will be prepared.

The vaccine doses will be distributed to the study centers while respecting the randomization block size.

5.2.2.2. Treatment allocation to the subject

The treatment allocation at the investigator site will be performed using an online central randomization system (SBIR). The treatment numbers will be allocated by kit. The randomization algorithm will use a minimisation procedure accounting for center.

When SBIR is not available, please refer to the SBIR user guide or the SPM for specific instructions.

After having checked the eligibility of the subject and obtaining the ICF, the site staff in charge of the vaccination will access SBIR. Upon providing the subject identification number, the randomization system will use the minimisation algorithm to determine the

treatment number to be used for the subject. Each treatment number must be recorded in the eCRF on the Vaccine Administration screen.

5.3. Method of blinding

The study will be conducted in an observer-blind manner.

Data will be collected in an observer-blind manner. By observer-blind, it is meant that during the course of the study, the vaccine recipient and those responsible for the evaluation of any study endpoint (e.g. safety, reactogenicity, and efficacy) will all be unaware of which vaccine was administered. To do so, vaccine preparation and administration will be done by authorised medical personnel who will not participate in any of the study clinical evaluation assays.

The laboratory in charge of the laboratory testing will be blinded to the treatment, and codes will be used to link the subject and study (without any link to the treatment attributed to the subject) to each sample.

5.4. General study aspects

Supplementary study conduct information not mandated to be present in this protocol is provided in the accompanying Study Procedures Manual (SPM). The SPM provides the investigator and the site personnel with administrative and detailed technical information that does not impact the safety of the subjects.

5.4.1. Screening

Volunteers must meet all inclusion criteria described in Section 4.3 and will be excluded from participation if they meet any of the exclusion criteria described in Section 4.4. All volunteers will sign an informed consent document to participate in the study and an additional informed consent statement for HIV testing, and for permission (or not) for photographs.

Volunteers will provide a medical history (with special attention to a review of cardiac, renal and neurological systems, previous history of splenectomy, recent antibiotic usage, and prior vaccine reactions), undergo physical examination, routine standard laboratory screening tests including: urinalysis (protein, erythrocytes), complete blood count (white blood cells [WBC], hemoglobin and platelets), serum chemistry profiles (creatinine, alanine aminotransferase [ALT] and aspartate aminotransferase [AST]) and additional laboratory screening tests (HIV, hepatitis B [HBV] and hepatitis C [HCV]).

All screening and follow-up diagnostic laboratory testing will be performed at Quest or LabCorp laboratories.

Subjects found to be seropositive for HIV or HCV, and/or found to be positive for HBsAg will be counselled by the investigator and referred to a health care provider for further evaluation. Positive results for HIV, HBV and HCV will be reported to public health agencies.

Urine beta-human chorionic gonadotrophin [β-HCG] test will be obtained from all female subjects of childbearing potential at screening, before each vaccination, as well as prior to malaria challenge to exclude pregnancy. Pregnancy tests will be performed at the investigator's site.

5.4.2. Risks to the subjects and precautions to minimize risk

5.4.2.1. Risks associated with vaccination and mitigation of risks

Ad35.CS.01 vaccine

As with many vaccines, the Ad35.CS.01 vaccine has shown mild to moderate injection site pain, swelling, or discomfort. There may also be some bruising or discoloring and rarely infection might occur. Other common side effects are fever, headaches, chills, nausea, vomiting, body aches, numbness or muscles weakness and tiredness. These reactions are usually greatest within the first 24-72 hours after vaccination and last 1-2 days. There is a rare possibility of an allergic reaction to the vaccine. As with any vaccine, unexpected serious adverse events, including allergic reactions to the vaccine components, may occur, although this has not been reported in prior studies with Ad35.CS.01.

In a previous study of consecutive administration of 3 doses of Ad35.CS.01 to various dose levels, some vaccinated individuals reported paresthesia and weakness in the vaccinated arm (refer to Section 1.1.2 for more details). The occurrence of new neurological symptoms after immunization will be monitored in this study in a standard way, using a standard neurological examination form assessing central and peripheral nervous systems, motor and sensory functions upon D0, D6 and D28 post first immunization (Appendix B).

Additional information

Results from an anti-HIV vaccine trial using an adenovirus type 5 (Ad5) vaccine expressing HIV antigens (ST***E***P trial) tested in 3,000 people was interrupted early because rather than being protective an increased incidence of HIV infections was seen in vaccinated individuals as compared to placebo. This difference was seen in individuals who were seropositive for Ad5 prior to study start. No pathophysiological explanation is available; one possibility was that the vaccine facilitated HIV infection in exposed individuals; another possibility is that people who got the vaccine were at more risk for HIV infection than the people who got the placebo; and a third possibility is that this is a 'chance' statistical finding. Although the malaria vaccine in this trial is based on a different adenovirus (Ad35), because of this unexplained finding in the STEP study, subjects will be counseled about exposure risks to HIV and how to avoid them.
(Amended 09 June 2011)

RTS,S/AS01$_B$ vaccine

The most frequent adverse reactions observed in previous clinical trials using the RTS,S antigen and AS01$_B$ adjuvant system include pain, swelling, erythema, and tenderness at the site of injection, and systemic symptoms such as low-grade fever and short-term flu-

like symptoms: fatigue, myalgia, headache, malaise. As with any vaccine, unexpected serious adverse events, including allergic reactions to the vaccine components, may occur, although this has not been reported in prior studies with RTS,S or the adjuvant.

Sometimes people who get vaccines with the adjuvant system used in this study report illnesses called autoimmune diseases as side effects. These are serious diseases. They result from an attack of the immune system on the body's own tissues. Examples of autoimmune diseases, or their symptoms, include nervous system disorders, inflammation of the blood vessels, impaired blood clotting, skin rashes, arthritis, thyroid gland disease, muscle pains or weakness, liver disease and kidney disease. It is possible that vaccines with this adjuvant system caused these side effects, but we do not yet know - because these same diseases can also occur in people who get other vaccines, or no vaccines at all.

As outlined above, the volunteers will be monitored closely during their participation in this study. The study vaccines have been prepared according to Good Manufacturing Practices (GMP). The vaccines will be administered under the supervision of an investigator and nurses equipped with drugs and equipment to immediately treat anaphylaxis.

While the study is observer-blinded, the investigator, or a person designated by the investigator, may request unblinding of a subject's treatment code in order to provide specific vaccine information for the study team or for health care providers caring for the volunteer subject(s). Refer to Section 8.8 for the procedure of emergency unblinding.

5.4.2.2. Risks associated with malaria challenge and mitigation of risks

Risks associated with malaria challenge include local inflammatory reactions to mosquito bites as well as the development of malaria infection. Additional risks include side effects from chloroquine (nausea, vomiting, diarrhea, headaches, pruritus) and the unlikely possibility of complications of malaria, which are seen during naturally acquired malaria when diagnosis and treatment are delayed and high levels of parasitemia develop. Under the carefully controlled conditions of this study, the chance of serious illness or death from malaria infection is very small. In challenge studies involving nearly 900 subjects over the past 17 years at the Walter Reed Army Medical Center (WRAIR), there has never been a case of mortality or excess morbidity requiring inpatient hospitalization. Transient abnormalities, such as fever, headache, mild anemia, leukopenia, splenomegaly, hepatic tenderness and fatigue, are expected consequences of malaria. In uncontrolled circumstances, malaria infections can lead to kidney, liver or brain damage, and death. Other possible risks may include a systemic allergic reaction to mosquito bites and the chance that the mosquitoes may transmit another infectious agent. No previous volunteers challenged using this procedure in the proposed study centers have developed a complication of malaria or have been unable to take the prescribed course of oral anti-malarials.

Two cardiovascular events have been described at another site, Radboud University Nijmegen Medical Center (RUNMC). In 2002, a 39 year old male was diagnosed with an inferoposterior myocardial infarction (MI) day 9 post-challenge [Verhage, 2005]. He was initially diagnosed with malaria via a blood smear on Day 8 but PCRs performed between days 5-9 (n = 10) were consistently negative and the blood smear was inferred to be a

false positive. The event was felt to be coincidental with the volunteer's participation in the trial.

The second event occurred in a 20 year old female who had received three doses at monthly intervals of an experimental malaria vaccine (recombinant LSA-3 formulated in alum) with severe headache noted after dose 3 [Nieman, 2009]. She underwent sporozoite challenge with the NF54 strain of parasite on Day 140 post-vaccination dose 1 (~day 75 post-dose 3). The volunteer became parasitemic on Day 11 after challenge (44 parasites/µl) and was treated with artemether-lumefantrine (Riamet®; not currently FDA-approved for use in the U.S.), and was cleared by day 14 when treatment was terminated. On day 16 she awoke with sub-sternal, non-radiating chest pain and was medically evaluated with pain dissipating within 30 minutes coincident to sublingual nitroglycerin administration. Electrocardiographic tracings revealed < 1mm ST elevation inferiorly. Cardio Phosphokinase levels were normal but the troponin level was elevated at 10.0 ng/mL. An echo revealed minimal inferior hypokinesis and an MRI was negative for cardiovascular disease or tissue damage.

A review of safety and clinical outcome of 47 volunteers that underwent experimental challenge with NF54 strain at NMRC, revealed that one subject experienced pleuritic chest pain lasting 2 days (onset of symptoms occurred one day before onset of parasitemia), and another subject reported shortness of breath lasting 1 day (onset 2 days after parasitemia and initiation of chloroquine). These 2 events were reported as unsolicited symptoms, and no further clinical laboratory information was obtained [Epstein, 2007]. Troponin levels analyzed in 38 volunteers with experimental malaria challenge at the University of Maryland, consistently remained negative during infection and at follow-up (personnel communication, Lyke).

Chloroquine phosphate and, if required, other appropriate FDA-approved drugs, such as quinine, doxycycline and **M**alarone® currently available in the U.S., will be used in standard doses to treat volunteers who become infected with malaria. These drugs will be available both at the study sites, as well as at the hotel used to house volunteers during the period of intense assessment for malaria following the sporozoite challenge. Such treatment minimizes the risk of developing a complicated malaria infection. **(Amended 09 June 2011)**

5.4.3. Malaria Challenge

Volunteers from both study groups completing their immunization course will be challenged with sporozoite-infected mosquitoes to determine whether the expected protective response has developed. Such challenges are mandated by the absence of any laboratory tests that will unequivocally predict protection. The challenge is scheduled to occur approximately 21 days (3 weeks) after the last vaccination visit (Study Day 77) and will be performed in a staggered fashion. The first 56 eligible subjects (28 subjects per study group) will be enrolled and vaccinated (Cohort A). Among the 56 vaccinated individuals a random selection of subjects eligible for challenge will be done according to the local SOP to ensure that a maximum of 46 vaccinated individuals (balanced by group) will progress to the challenge phase. The remaining subjects will not be challenged but will be followed for safety and immunogenicity up to 6 months after the last vaccination.

Unimmunized infectivity control volunteers will also be challenged to verify the adequacy of the challenge. The use of 4 to 6 unimmunized, malaria-naive controls per challenge day is adequate since the procedure described has generally successfully infected such volunteers [Gordon, 1995]. The challenge of vaccinated subjects from Cohort A will be done at the WRAIR Insectary.

After safety and efficacy assessment for Cohort A, subjects from Cohort B and C (approximately 56 subjects per cohort) will be vaccinated. Of these subjects, a maximum of 46 vaccinated study participants per cohort will progress to the challenge phase. The challenge of Cohorts B and C will be conducted in parallel at WRAIR Insectary and the University of Maryland, CVD, respectively. Malaria-infected mosquitoes for challenge at the University of Maryland will be transported by WRAIR investigators to the insectaries at the University of Maryland for challenge of human subjects.

The *A. stephensi* female mosquitoes infected with *P. falciparum* parasites of the NF54/3D7 strain used for challenge will be reared at WRAIR in their insectary facilities. Volunteers recruited and vaccinated at the University of Maryland will undergo challenge at the insectary suites located within the Center for Vaccine Development. These mosquitoes require carefully regulated environmental conditions such as temperature and humidity. At a designated time, WRAIR entomologic staff will accompany the mosquitoes, placed in secure transfer vessels, from WRAIR to the University of Maryland (a distance of approximately 30 miles from 503 Robert Grant Avenue in Silver Spring, MD). Upon transfer to the University of Maryland, CVD the containers of mosquitoes will be placed in the CVD insectary utilizing incubators that will maintain appropriate environmental conditions. The security of the insectary is critical and, as such, every effort is made to restrict access to the insectary and provide for a secure location. The University of Maryland has a protected suite of 920 sq ft. devoted to an insectary, containment area, laboratory, and microscopy rooms, as well as incubation facilities for proper maintenance of temperature and humidity. A two-door barrier system along with negative pressure blowers (Dyneform Model ICL-48 (Windsor, CT)) prevent the migration of infected mosquitoes from the high containment challenge room to the waiting antechamber. An additional barrier door prevents mosquito migration into the clean molecular laboratory space. The successful transfer of mosquitoes to and from WRAIR to the CVD has occurred on several previous occasions.

Volunteers undergoing malaria challenge will receive an emergency notification card that outlines their participation in the study with details on the exposure to malaria, as well as the appropriate investigator contact telephone numbers. Details of this information may vary, depending on the venue selected for the hotel/inpatient phase of the study.

5.4.3.1. Contraindications to malaria challenge

The only absolute contraindication to mosquito challenge is pregnancy. If this occurs after immunization but before undergoing challenge, the participant must not be challenged and they will be followed for the duration of the study or pregnancy whichever is longer.

If a volunteer has a minor illness but does not have a fever during the pre-challenge assessment, they may be challenged at the discretion of the clinical investigator. If the

volunteer is moderately to severely ill with a fever, they will not be challenged and they will be followed for the duration of the study.

5.4.3.2. Parasite and mosquito strains

The 3D7 clone of *P. falciparum* is a human malaria isolate that has never been passed through monkeys, is well adapted to culture, is a good producer of gametocytes that can infect mosquitoes, is susceptible to currently available, approved, anti-malarial compounds, and has previously been used in the WRAIR challenge model to successfully infect human volunteers under a Biologic Master File (BMF) submitted to the FDA. Master seed lots of these parasites have been developed and stored at WRAIR. All blood products used for malaria and mosquito culturing will be commercially tested for HIV, hepatitis B, C, and syphilis. The mosquitoes used will be laboratory-born and reared *Anopheles stephensi.* This species is relatively easy to maintain in the laboratory for up to 21 days after infection with malaria, feed readily on humans, and is able to transmit malaria to volunteers [Chulay, 1986].

5.4.3.3. Infection of Human Volunteers

Mosquitoes infected approximately 2-3 weeks earlier that are likely to contain sporozoites in their salivary glands will be allowed to feed on the volunteers. For each volunteer, five mosquitoes will be allowed to feed over five minutes, after which they will be dissected to confirm how many were infected, and the salivary glands scored. If required, additional mosquitoes will be allowed to feed until a total of five infected mosquitoes with a minimum 2+ salivary gland score have fed [Wirtz, 1987]. The mosquito feedings will be performed in the secure insectary of the Department of Entomology at WRAIR for two study cohorts and at the University of Maryland, CVD for the third study cohort. Volunteers will be observed for 30 minutes following completion of the sporozoite challenge in order to assess them for any evidence of acute allergic reactions related to mosquito exposure. To date, no severe allergic reactions related to mosquito bites have been documented in the context of the WRAIR malaria challenge model. Routinely, transient local allergic reactions (itching, rash) typical of mosquito bites occur at sites of the bites.

5.4.3.4. Determining Parasitemia

Post-challenge, parasitemia will be determined by microscopy of Giemsa-stained thick blood films (smear) and PCR. Microscopy will be performed on thick smears using a standard, validated SOP. Slides will initially be screened by trained microscopy staff. In the case that one or more parasites are detected, the screening microscopist will obtain confirmation of the diagnosis from an expert microscopist if the screening microscopist is not certified. A total blood volume of approximately 0.5 µl will be read for each individual. In the case of a symptomatic (as determined by the Principal Investigator) or febrile (oral, axillary or tympanic temperature ≥ 38.0°C or 100.4°F) volunteer, the quantity of blood examined will be doubled to 1.0 µl (± 0.1 µl). Positive parasitemia will be defined as minimum of 2 unambiguous malaria parasites noted on the thick smear.

All blood films (positive and negative) will be archived at the study centers for later re-examination and confirmation, if required.

5.4.3.5. Management of Infected Human Volunteers

The pre-patent period for *P. falciparum* in man normally averages 9-12 days. In previous studies, the pre-patent period in controls varied from 7-18 days. The shortest reported pre-patent period in man is 5 days, and the longest is 25 days [Ballou, 1987]. An immunized individual who does not have complete protection may have a prolongation of the parasite pre-patent period.

Volunteers will return at the study clinic on Day 1 after their challenge for evaluation by a study investigator. Beginning on Day 5 after their challenge, volunteers will be seen and evaluated daily by a study investigator and blood for PCR and smears will be drawn to check for presence of parasites as described in Section 5.4.3.4. If fever or symptoms develop at any time, blood films will be obtained more frequently (every 6 to 12 hours), and a study investigator will evaluate the volunteer. A confirmed positive result will be relayed immediately to the on-call investigator/study personnel by the microscopists. The infection will be treated early (i.e., as soon as parasites can be identified on thick smear) according to the treatment regimen outlined in Section 5.4.3.6. In many cases, malaria infection has often been identified and treated based upon positive blood smear results prior to the development of clinical symptoms of malaria.

Beginning on Day 9 post-challenge (Study Day 86), a group of hotel rooms in the local area of WRAIR or, in the case of the University of Maryland, an inpatient facility, The Shin Nippon Biomedical Laboratories (SNBL), will be reserved for malaria-challenged volunteers. The volunteers will be required to spend their evenings there to allow for more rapid assessment of any potential symptoms of experimental malaria during the hours that the study centers are closed. There will be an investigator "on-call" and available for volunteer assessment on each day. During the post-challenge phase, it is possible that the investigator might be off-site but still be accessible via phone or pager. There will also be qualified study personnel on site 24 hours per day during the hotel/inpatient facility phase of the study.

During the hotel/inpatient facility phase, all challenged volunteers will be assessed on a daily basis in an identical manner. An evaluation will be done each morning (headache, muscle aches, etc.) and blood will be drawn for PCR and smear. All challenged volunteers will be required to check in with clinical staff by telephone call or in-person each evening during the hotel/inpatient facility stay until they are positive for malaria. They will be asked the same health status questions that were asked during the morning evaluation (headache, muscle aches, etc.). At any time required, the on-duty investigator will arrange for the timely production of blood smears, along with their examination and interpretation, in order to treat rapidly those volunteers in whom therapy for malaria is indicated. Once a positive smear is identified, daily blood films will continue to be obtained until three consecutive films are negative (separated by greater than 12 hours). A complete blood count and serum chemistry tests will be done when parasites are initially found in the blood.

The maximum hotel/inpatient facility stay for malaria-challenged volunteers should be approximately 10 days (Day 9-18 post-challenge). A volunteer who develops malaria, is treated, and has 3 consecutive negative malaria smears (separated by greater than 12 hours), will not need to remain in the hotel/inpatient facility. The Investigators will be

responsible for accounting for any volunteers who do not arrive in the hotel/inpatient facility during the challenge phase. If required, the Investigators will physically locate and treat any malaria-infected volunteer who is unable to maintain the follow-up dictated by this study.

If infection does not develop within 18 days, the volunteer will be released from staying nightly at the hotel/inpatient facility. Volunteers who do not develop malaria will be required to come to the clinical center for evaluation and blood drawing for PCR and smears every two days up to 28 days post-challenge (Study Day 105). Additional blood volume will be drawn on Study Day 105 for immunological, haematological and biochemical safety analysis. A volunteer who develops malaria and has 3 consecutive (separated by greater than 12 hours) negative smears following initial treatment may be excused from ***the remaining hotel/inpatient facility visits and*** the late post-challenge clinic visits at Study Day ***97, 99, 101 and 103 (***20, 22, 24 ***and*** 26 ***days post-challenge)*** but will be required to come to the clinic center at Study Day 105 (28 days post-challenge). Telephone contact will be made if the volunteer does not keep a scheduled follow-up appointment. **(Amended 09 June 2011)**

5.4.3.6. Malaria Treatment

During the evaluation of protective efficacy, as soon as a malaria infection is documented in a volunteer, he/she will be treated with standard doses of oral chloroquine (a total of 1500 mg chloroquine base: 600 mg base initially, followed by 300 mg base given approximately 6, 24, and 48 hours later) under direct observation. This regimen has been 100% effective in previous WRAIR malaria vaccine studies using the same malaria strain as will be used in this challenge model. Such early treatment minimizes the risk of developing a complicated malaria infection.

The malaria strain used for challenge (*P. falciparum* strain NF54/clone 3D7) is sensitive to several currently available, licensed, anti-malarial drugs that are safe, effective and have a low incidence of side effects. The investigators will have available approved antipyretics, such as acetaminophen and ibuprofen, for subjects experiencing fever and myalgias. In addition, other approved medications will be available to the investigators, which may include, but are not limited to: acetaminophen with codeine (Tylenol #3®), trimethobenzamide (Tigan®), and loperamide (Imodium®) to treat other signs/symptoms as necessary. Investigators will always assure that subjects do not have underlying allergies to any of these medications prior to their use. An alternative antipyretic will be provided if the subject is allergic to a prescribed drug.

It is anticipated that treatment of malaria will be curative, since relapses do not occur after adequate treatment of *P. falciparum* infections. No previous volunteer infected and treated by WRAIR has had a malaria relapse. ***S***ubjects will be advised to contact the study physician, or to advise their personal physician of their participation in this malaria study, if fever, headache, or other symptoms possibly related to malaria develop at any time within one year after completion of the study. In the unlikely event that malaria recurs, the subject will be retreated with atovaquone/proguanil (Malarone®) standard oral dosage of 1 gram/400 mg (4 adult tablets per day for 3 consecutive days), chloroquine as outlined above, or with a combination of quinine (650 mg three times a day for three

days) and doxycycline (100 mg twice daily for 7 days), or Coartem (20 mg artmether and 120 mg lumefantrine) twice daily for 3 days. **(Amended 09 June 2011)**

No human viruses are known to be transmitted by colonized *Anopheles* mosquitoes. Blood purchased for malaria feedings has been commercially tested using FDA-approved test methods for antibodies to HIV, hepatitis C, and syphilis, as well as for the presence of hepatitis B surface antigen; all blood has tested negatively for these tests. No documented cases of HIV or viral hepatitis transmission from mosquitoes to humans have occurred. The risk of accidentally transmitting malaria to a person in the community will be negligible because:

- The infected mosquitoes will be raised in the secure insectary at WRAIR.
- All malaria challenges occur in a secure insectary.
- With the exception of transfer of the infected mosquito lot from WRAIR to the University of Maryland, CVD, the infected mosquitoes never leave the secured insectary area at any time.
- Malaria infections in volunteers will be treated promptly before gametocytes can develop (generally 10 days after the development of patent malaria), thus the risk of transmission to local mosquitoes is reduced.

5.4.4. Safety monitoring plan

Vaccination and challenge will be performed in a staggered fashion.

The study will include three cohorts (Cohort A, B and C) of approximately equal size which will be enrolled, vaccinated then challenged separately. Each cohort which enters the vaccination phase will include approximately 56 subjects. Among the 56 vaccinated individuals a random selection of subjects eligible for challenge will be done according to the local SOP to ensure that a maximum of 46 vaccinated individuals (balanced by group) will progress to the challenge phase. The remaining subjects will not be challenged but will be followed for safety and immunogenicity up to 6 months after the last vaccination.

Vaccination and challenge will be performed first in Cohort A. Safety and efficacy data post-challenge from this initial cohort and 4 to 6 infectivity controls will be reviewed prior to the start of vaccination in Cohorts B and C, based on pre-defined group holding criteria for safety review (see Section 5.4.4.1) and pre-defined criteria for efficacy futility analysis (see Section 5.4.4.2). Vaccination and challenge in Cohorts B and C is likely to be performed in parallel at two different sites (see Section 4.2 for more details).

In addition, the PI will have the responsibility to withdraw from further vaccination individual study participants who present at least one of the pre-defined stopping rules for individual subjects (see Section 5.4.4.1).

In each center and in each cohort, vaccination with dose 1 and dose 2 will happen over two days. At the end of day 1 after vaccination dose 1 and dose 2, before start of day 2, the local safety monitor will review the immediate post-vaccination surveillance data before authorizing progression to the next day of vaccination.

5.4.4.1. Safety review

The safety monitoring for this study will be performed by a Safety Monitoring Committee (SMC). The SMC will consist of five experienced individuals not otherwise participating on the study team, selected and approved by the study sponsor and PI. Locally, for each study center a local medical safety monitor independent from the study team (not otherwise participating to any other study-related activity) will contribute to the safety assessment according to pre-defined Operating Procedures. The SMC will include one member each from GSK Biologicals, MVI, Crucell and the two local safety monitors (one from WRAIR and one from University of Maryland, CVD). The chairperson will be designated by the sponsor.

The SMC, operating under a charter, will meet in person or through teleconferences on a regular basis. Decisions, based on face to face, teleconference or email exchanges will be documented. Hereafter, the data reviewed by the SMC and applicable predefined stopping rules are described in detail.

5.4.4.1.1. Data reviewed by the SMC on a real-time basis

– All Serious Adverse Events

5.4.4.1.2. Data review by the SMC between vaccine doses (for each Cohort separately only)

Listings will be produced and reviewed once all subjects have been followed up for 7 days post vaccination (day 0 to day 6) after vaccination dose 1 and dose 2, prior to authorizing progression to dose 2 and dose 3, respectively. No stopping rule will be pre-defined when considering progression from dose 1 to dose 2, as only RTS,S/$AS01_B$ will be administered as from dose 2, considering that RTS,S/$AS01_B$ has a well characterized, favourable safety profile, with a pediatric formulation currently in Phase 3 evaluation. As RTS,S/$AS01_B$ has never been administered before in individuals vaccinated with Ad35.CS.01, and considering that past immunization with Ad35.CS.01 may influence the reactogenicity to subsequent RTS,S/$AS01_B$, stopping rules will be pre-defined when considering progression from dose 2 to dose 3.

The following listings will be produced and reviewed by the SMC when considering dose 1 to dose 2 and dose 2 to dose 3 progression:

– All reactogenicity data, presented by dose, all and grade 3, relatedness assessment (all and grade 3), tabulated by group

– All laboratory values and laboratory abnormalities estimated to be related to vaccination, presented according to protocol toxicity grading, tabulated by group

– All unsolicited events presented by individual events (separately, Serious Adverse Events and non serious Adverse Events), including study group, last vaccine received, time to onset, relatedness assessment, severity grade, outcome, decision to withdraw from further investigational product administration

– All unsolicited events (separately, Serious Adverse Events and non serious Adverse Events) presented as cumulative tables by group

- Results of standardized neurological examinations tabulated by group

The following pre-defined stopping rules will apply when considering progression from dose 2 to dose 3:

- Solicited local adverse events:
  - More than 25% of subjects vaccinated with RTS,S/$AS01_B$ after having received Ad35.CS.01 develop a Grade 3 solicited local adverse event beginning within 2 days after vaccination (day of vaccination and one subsequent day) and persisting at Grade 3 for > 2 consecutive days
- Solicited systemic adverse events:
  - more than 25% of subjects vaccinated with RTS,S/$AS01_B$ after having received Ad35.CS.01 develop a Grade 3 solicited systemic adverse event beginning within 2 days after vaccination (day of vaccination and one subsequent day) and persisting at Grade 3 for > 2 consecutive days.

5.4.4.1.3. Cohort A data review by the SMC prior to vaccinations in Cohort B and Cohort C:

An interim unblinded SMC report including all available safety and reactogenicity data, will be produced by an independent statistician according to an agreed pre-defined Report and Analysis Plan, after the Cohort A has completed the one month post challenge visit. The SMC will review this report prior to authorizing vaccinations to proceed in Cohort B and Cohort C. Vaccination of the next cohort of study participants can be put on hold by the SMC if either of the following sets of holding criteria described below is met:

Holding rules

- Solicited local adverse events:
  - More than 25% of Ad35.CS.01 doses are followed by Grade 3 solicited local adverse event beginning within 2 days after Ad35.CS.01 vaccination (day of vaccination and one subsequent day) and persisting at Grade 3 for > 2 consecutive days
  - More than 25% of first doses of RTS,S/$AS01_B$ in subjects having received Ad35.CS.01 are followed by Grade 3 solicited local adverse event beginning within 2 days after vaccination (day of vaccination and one subsequent day) and persisting at Grade 3 for > 2 consecutive days
  - More than 25% of second doses of RTS,S/$AS01_B$ in subjects having received Ad35.CS.01 are followed by Grade 3 solicited local adverse event beginning within 2 days after vaccination (day of vaccination and one subsequent day) and persisting at Grade 3 for > 2 consecutive days
- Solicited systemic adverse events:
  - More than 25% of Ad35.CS doses are followed by Grade 3 solicited systemic adverse event estimated to be related to vaccination beginning within 2 days

after Ad35.CS.01 vaccination (day of vaccination and one subsequent day) and persisting at Grade 3 for > 2 consecutive days

- More than 25% of first doses of RTS,S/AS01$_B$ in subjects having received Ad35.CS.01 are followed by Grade 3 solicited systemic adverse event estimated to be related to vaccination beginning within 2 days after vaccination (day of vaccination and one subsequent day) and persisting at Grade 3 for > 2 consecutive days
- More than 25% of second doses of RTS,S/AS01$_B$ in subjects having received Ad35.CS.01 are followed by Grade 3 solicited systemic adverse event estimated to be related to vaccination beginning within 2 days after vaccination (day of vaccination and one subsequent day) and persisting at Grade 3 for > 2 consecutive days

- Unsolicited adverse events
  - a serious adverse event considered related to vaccination occurs in a study participant from the Ad35.CS.01 – RTS,S/AS01$_B$ – RTS,S/AS01$_B$ group
  - more than 25% of subjects from the Ad35.CS.01 – RTS,S/AS01$_B$ – RTS,S/AS01$_B$ group develop any Grade 3 unsolicited adverse event considered related to vaccination and persisting at Grade 3 for > 2 consecutive days.
- Standard neurological evaluation
  - more than 10% of subjects from the Ad35.CS.01 – RTS,S/AS01$_B$ – RTS,S/AS01$_B$ group develop abnormal neurological symptoms captured by standardized neurological evaluation (Appendix B).
- Laboratory adverse events
  - more than 25% of subjects from the Ad35.CS.01 – RTS,S/AS01$_B$ – RTS,S/AS01$_B$ group develop any Grade 3 laboratory adverse event considered related to vaccination.
  - more than 10% of subjects develop hematuria, not taking into account menstruating females if the hematuria coincides with their regular monthly time for their period or urinary tract infection.

If a holding rule has been met, vaccination of subjects in the subsequent cohort will only start if GSK, Crucell, MVI and investigators agree to it, following discussions with the SMC. All IRBs will be notified if a holding rule is activated or released after SMC review.

Individual stopping rules

In addition to the above stated group holding rules, stopping rules for individual subjects will apply (i.e., indications to withdraw individuals from further vaccinations). These will apply to Cohort A, B and C. The PI will have the responsibility to withdraw from further vaccination individual study participants who present at least one of the following stopping rules for individual subjects:

- Local reactions: upon investigator discretion.

- Systemic solicited adverse events:
  - the subject develops a Grade 3 systemic solicited adverse event beginning within 2 days after vaccination (day of vaccination and one subsequent day) and persisting at Grade 3 for > 2 consecutive days.
- Unsolicited adverse events
  - the subject has any Grade 3 adverse event considered related to vaccination, persisting at Grade 3 for >2 consecutive days.
  - the subject has a serious adverse event considered related to vaccination.
  - the subject has an acute allergic reaction or anaphylactic shock following the administration of vaccine investigational product.
- Laboratory adverse events
  - the subject has any Grade 3 laboratory adverse event considered related to vaccination.
  - the subject, excluding menstruating female, develops hematuria considered related to vaccination.

The PI may, using discretion, ask for the study to be placed on hold and an SMC meeting to be held for any single event or combination of multiple events which, in professional opinion, jeopardize the safety of the subjects or the reliability of the data.

Neurological event monitoring:

The safety evaluation of study participants will include the use of a standard neurological examination assessing central and peripheral nervous systems, motor and sensory functions upon D0, D6 and D28 post first immunization. New onset of neurological diseases will be reported as AE/SAEs accordingly. The standard neurological evaluation form (Appendix B) will be used for documentation of the events with objective central or peripheral neurological deficits, in addition to full medically appropriate evaluation according to local procedures.

5.4.4.2. Efficacy futility review

The planned futility analyses will be conducted by an external statistician who will ensure the maintenance of the blinding of all parties involved in the trial.

Futility analyses will be conducted on efficacy data collected up to 28 days post-challenge (Study Day 105) for the first 46 subjects vaccinated and challenged (Cohort A).

Vaccination of Cohorts B and C will only proceed if the calculated point estimate of increase of vaccine efficacy (VE) in the Ad35.CS.01 – RTS,S/AS01$_B$ group over RTS,S/AS01$_B$ alone is over 0%, providing a 4 % risk of stopping the trial if the true increase in VE is 50%, assuming an attack rate of 50% in the RTS,S/AS01$_B$ group. The probability to stop for futility using these criteria if true increase in vaccine efficacy is 0% is of 40%. The increase in VE of the Ad35.CS.01 group over the RTS,S/AS01$_B$ alone

group is defined as 100*(1-AR Ad35.CS.01 / AR RTS,S) where AR is the attack rate in the 2 groups.

If the above criteria are not met, then vaccination of consecutive cohorts will be performed only if there is agreement between all parties and the study PI.

5.5. Outline of study procedures

There will be in total 42 study visits. All study visits during the screening, vaccination challenge and follow-up epochs are applicable for vaccinated volunteers. For infectivity controls, the screening visit 2 during the screening epoch and the study visits during the vaccination epoch are NOT applicable.

The list of study procedures can be found in Table 7 through Table 10 for vaccinees. For infectivity controls, the list of study procedures can be found in Table 7, Table 9 and Table 10.

Table 7 List of study procedures – Screening epoch (vaccinees and infectivity controls)

Epoch	Screening epoch	
**Study visit**	**1**	**2¶**
**Study day**	**-90 to -3**	**-7 to -1**
Screening, briefing, informed consent	●	
Comprehension assessment	O	
Check inclusion/exclusion criteria	●	●
Assign subject number	●	
Record demographic data	●	
Medical history	●	
Physical examination	●	O
Check NHANES I criteria and EKG	●	
Pregnancy test: urine β-HCG	●	
Reporting of SAEs related to study participation or to a concurrent GSK medication/vaccine or any fatal SAE	●	●
Record any concomitant medication/vaccination	●	●
Urine analysis for safety **	●	
Blood sampling for safety analysis (7 mL)	● §, v	
Blood sampling for additional screening analysis (15 mL)	● *, v	
Blood sampling for humoral immunity (20 mL)		● v
Blood sampling for CMI (60 mL)		● v
Blood sampling for exploratory cytokine/chemokine Luminex (2 mL)		● v
Blood sampling for microarray and deep sequencing analysis (12 mL)		● v
Blood sampling for plasmablasts FACS and B cell ELISPOT (10 mL)		● v
**Total blood volume in mL per visit**	**22**	**104**
Cumulative blood volume – Immunization groups (mL)	22	126
Cumulative blood volume – Infectivity controls (mL)	22	-

● is used to indicate a study procedure that requires documentation in the individual eCRF
○ is used to indicate a study procedure that does not require documentation in the individual eCRF
¶Study visit **NOT** applicable for the infectivity controls
§ Safety labs include hemoglobin, WBC, PLT, ALT, AST, creatinine
* Additional screening analysis include HIV, HCV, HBV
** ***One urine re-sampling is allowed, if necessary, at each time point that a urine sample is taken during the study, at investigators discretion.***
v ***One repeat blood draw to refine or confirm whether a potential participant is eligible or not, or to complete an insufficient blood volume is allowed during the screening, when medically justified, at investigators discretion***
(Amended 09 June 2011)

Table 8 List of study procedures - Vaccination epoch (vaccinees)

Epoch	Vaccination epoch																
**Study visit**	**3**	**4**	**5**	**6**	**7**	**8**	**9**	**10**	**11**	**12**	**13**	**14**	**15**	**16**	**17**	**18**	**19**
**Study day**	**0**	**1**	**2**	**3**	**6**	**14**	**28**	**29**	**30**	**31**	**34**	**42**	**56**	**57**	**58**	**59**	**62**
Physical examination	O	O	O	O	O	O	O	O	O	O	O	O	O	O	O	O	O
Pregnancy test: urine β-HCG	●						●						●				
Randomization	O																
Assign treatment number	●																
Check screening lab results	●																
Check contraindications, warnings and precautions to vaccination	●						●						●				
Pre-vaccination body temperature	●						●						●				
Vaccination	●						●						●				
Distribution of diary cards	O						O						O				
Recording of solicited AEs (Days 0-6)	●	●	●	●	●		●	●	●	●	●		●	●	●	●	●
Return of diary cards					O						O						O
Recording of non-serious AEs	●	●	●	●	●	●	●	●	●	●	●	●	●	●	●	●	●
Reporting of SAEs	●	●	●	●	●	●	●	●	●	●	●	●	●	●	●	●	●
Reporting of pregnancies	●	●	●	●	●	●	●	●	●	●	●	●	●	●	●	●	●
Standard neurological assessment	●				●		●										
Record any concomitant medication/vaccination	●	●	●	●	●	●	●	●	●	●	●	●	●	●	●	●	●
Record any intercurrent medical condition		●	●	●	●	●	●	●	●	●	●	●	●	●	●	●	●
Urine analysis for safety **					●		●										
HLA typing (buccal swab)	●																
Blood sampling for safety analysis (7 mL) z	● §				● §		● §				● §		● §				● §
Blood sampling for humoral immunity (20 mL) z							●						●				
Blood sampling for CMI (60 mL) z						●						●					
Blood sampling for exploratory cytokine/chemokine Luminex (2 mL) z		●	●		●	●	●	●			●		●	●			●
Blood sampling for microarray and deep sequencing analysis (12 mL) z		●	●		●	●	●	●			●		●	●			●
Blood sampling for plasmablasts FACS and B cell ELISPOT (10 mL) z							●				●		●				●
**Total blood volume in mL per visit**	**7**	**14**	**14**		**21**	**74**	**51**	**14**			**31**	**60**	**51**	**14**			**31**
Cumulative blood volume – Immunization groups (mL)	133	147	161		182	256	307	321			352	412	463	477			508

● is used to indicate a study procedure that requires documentation in the individual eCRF

○ is used to indicate a study procedure that does not require documentation in the individual eCRF

§ Safety labs include hemoglobin, WBC, PLT, ALT, AST, creatinine

** ***One urine re-sampling is allowed, if necessary, at each time point that a urine sample is taken during the study, at investigators discretion.***

[z] ***One repeat blood drawn is allowed, if necessary, at each time point that a blood sample is taken during the study, at investigators discretion.***
(Amended 09 June 2011)

Table 9 List of study procedures - Challenge epoch (vaccinees and infectivity controls)

Epoch	Challenge																				
**Challenge phase**	**DoC†**	**Early post-challenge**					**Hotel/inpatient phase[v]**										**Late post-challenge[v]**				
**Study visit**	**20**	**21**	**22**	**23**	**24**	**25**	**26**	**27**	**28**	**29**	**30**	**31**	**32**	**33**	**34**	**35**	**36**	**37**	**38**	**39**	**40**
**Study day**	**77**	**78**	**82**	**83**	**84**	**85**	**86**	**87**	**88**	**89**	**90**	**91**	**92**	**93**	**94**	**95**	**97**	**99**	**101**	**103**	**105**
**Challenge day**	**0**	**1**	**5**	**6**	**7**	**8**	**9**	**10**	**11**	**12**	**13**	**14**	**15**	**16**	**17**	**18**	**20**	**22**	**24**	**26**	**28**
Physical examination	O	O	O	O	O	O	O	O	O	O	O	O	O	O	O	O	O	O	O	O	O
Pregnancy test: urine β-HCG	●																				
Check contraindication to challenge	●																				
Sporozoite challenge	●																				
Emergency notification card delivery	O																				
Recording of non-serious AEs within 30 days post-challenge	●	●	●	●	●	●	●	●	●	●	●	●	●	●	●	●	●	●	●	●	●
Reporting of SAEs	●	●	●	●	●	●	●	●	●	●	●	●	●	●	●	●	●	●	●	●	●
Reporting of pregnancies	●	●	●	●	●	●	●	●	●	●	●	●	●	●	●	●	●	●	●	●	●
Record any concomitant medication/vaccination	●	●	●	●	●	●	●	●	●	●	●	●	●	●	●	●	●	●	●	●	●
Record any intercurrent medical condition	●	●	●	●	●	●	●	●	●	●	●	●	●	●	●	●	●	●	●	●	●
Blood sampling for PCR and smear (2mL)[z]			●	●	●	●	●	●	●	●	●	●	●	●	●	●	● ‡	● ‡	● ‡	● ‡	● ‡
Blood sampling for safety analysis (7 mL)[z]	● §													● # §							● §
Blood sampling for humoral immunity (20 mL)[z]	●																				●
Blood sampling for CMI (60 mL)[z]	●																				●
Blood sampling for exploratory cytokine/chemokine Luminex (2 mL)[z]	●	●	●																		●
Blood sampling for microarray and deep sequencing analysis (12 mL)[z]	●	●	●																		
**Total blood volume in mL per visit**	**101**	**14**	**16**	**2**	**2**	**2**	**2**	**2**	**2**	**2**	**2**	**2**	**2**	**9#**	**2**	**2**	**2**	**2**	**2**	**2**	**91**
Cumulative blood volume – Immunization groups (mL)	609	623	639	641	643	645	647	649	651	653	655	657	659	668	670	672	674	676	678	680	771
Cumulative blood volume – Infectivity controls (mL)	123	137	153	155	157	159	161	163	165	167	169	171	173	182	184	186	188	190	192	194	285
Primary analysis																					●[w]

§ Safety labs include hemoglobin, WBC, PLT, ALT, AST, creatinine
† DoC: Day of challenge
# Day of parasitemia and number of parasitemic individuals is undetermined
‡ For volunteers who develop malaria: blood smears and PCR may be discontinued once the volunteer has 3 consecutive (separated by greater than 12 hours) negative smears following initial treatment.

[v]A volunteer who develops malaria and has 3 consecutive (separated by greater than 12 hours) negative smears following initial treatment may be excused from the remaining hotel/inpatient facility visits and the late post-challenge clinic visits at Study Day ***97, 99, 101 and 103*** (20, 22, 24 ***and*** 26 ***days*** post-challenge).
[w] An interim analysis for futility will be performed on safety and efficacy data collected up to 28 days post-challenge (Study Day 105) for the first 46 subjects vaccinated and challenged (Cohort A). A primary analysis will be performed on all efficacy, immunogenicity and safety data collected up to 28 days post-challenge (Study Day 105) on all cohorts.
[z] One repeat blood drawn is allowed, if necessary, at each time point that a blood sample is taken during the study, at investigators discretion.
● is used to indicate a study procedure that requires documentation in the individual eCRF
○ is used to indicate a study procedure that does not require documentation in the individual eCRF
(Amended 09 June 2011)

Table 10 List of study procedures - Follow-up epoch (vaccinees and infectivity controls)

Epoch	Follow-up epoch	
**Study visit**	**41**	**42**
**Study day**	**140**	**236**
**Challenge day**	**63**	**159**
Physical examination	O	O
Reporting of SAEs	●	●
Reporting of pregnancies	●	●
Record any concomitant medication/vaccination	●	●
Record any intercurrent medical condition	●	●
Blood sampling for safety analysis (7 mL)[z]	● §	● §
Blood sampling for humoral immunity (20 mL)[z]	●	●
Blood sampling for CMI (60 mL) [z]	●	●
**Total blood volume in mL per visit**	**87**	**87**
Cumulative blood volume – Immunization groups (mL)	8***58***	94***5***
Cumulative blood volume – Infectivity controls (mL)	37***2***	4***59***
Study conclusion		●

● is used to indicate a study procedure that requires documentation in the individual eCRF
○ is used to indicate a study procedure that does not require documentation in the individual eCRF
§ Safety labs include hemoglobin, WBC, PLT, ALT, AST, creatinine
[z] ***One repeat blood drawn is allowed, if necessary, at each time point that a blood sample is taken during the study, at investigators discretion.***
(Amended 09 June 2011)

It is the investigator's responsibility to ensure that the intervals between visits are strictly followed. Table 11 and Table 12 present the details for intervals between study visits for vaccinees and infectivity controls, respectively..

Table 11 Intervals between study visits for vaccinees

Interval	Optimal length of interval[1]	Maximum interval allowed[2]
Visit 1 → Visit 3 (1st vac)	0 to 90 days	90 days
Visit 3 → Visit 7	6 days	+/- 1 days
Visit 3 → Visit 8	14 days	+/- 2 days
Visit 3 → Visit 9 (2nd vacc)	28 days	+/- 4 days
Visit 9 → Visit 13	6 days	+/- 1 days
Visit 9 → Visit 14	14 days	+/- 2 days
Visit 9 → Visit 15 (3rd vacc)	28 days	+/- 4 days
Visit 15 → Visit 19	6 days	+/- 1 days
Visit 15 → Visit 20 (challenge)	21 days	+/- 7 days
Visit 20 → Visit 40	28 days	+/- 2 days
Visit 40 → Visit 41	35 days	+/- 7 days
Visit 41 → Visit 42	96 days	+/- 7 days

[1] Whenever possible the investigator should arrange study visits within this interval
[2] Subjects will not be eligible for inclusion in the cohort for ATP analysis of immunogenicity and efficacy if they make the study visit outside this interval

Table 12 Intervals between study visits for infectivity controls

Interval	Optimal length of interval[1]	Maximum interval allowed[2]
Visit 1 → Visit 20 (challenge)	0 to 90 days	90 days
Visit 20 → Visit 40	28 days	+/- 2 days
Visit 40 → Visit 41	35 days	+/- 7 days
Visit 41 → Visit 42	96 days	+/- 7 days

[1] Whenever possible the investigator should arrange study visits within this interval
[2] Subjects will not be eligible for inclusion in the cohort for ATP analysis of immunogenicity and efficacy if they make the study visit outside this interval

5.6. Detailed description of study procedures

5.6.1. Procedures prior to study participation

5.6.1.1. Informed consent and protocol comprehension assessment

Before performing any other study procedure, the signed informed consent for clinical study and for HIV testing needs to be obtained from the subject. Refer to Section 5.1 for the requirements on how to obtain informed consent.

A protocol comprehension assessment using a short multiple-choice quiz needs to be performed prior to enrollment for each subject.

5.6.2. Procedures during the screening epoch

5.6.2.1. Check inclusion and exclusion criteria

Check all applicable inclusion and exclusion criteria as described in Sections 4.3 and 4.4 before enrollment.

5.6.2.2. Assign subject number

Subject number will be assigned at the screening visit 1. **(Amended 09 June 2011)**

5.6.2.3. Check electrocardiogram and NHANES I criteria

Perform an electrocardiogram (EKG) and check NHANES I criteria before enrollment. Refer to Appendix A for details on NHANES I criteria.

5.6.2.4. Collect demographic data

Record demographic data such as age, gender and geographic ancestry in the subject's eCRF.

5.6.2.5. Medical history

Obtain a medical history (with special attention to a review of cardiac and renal systems, previous history of splenectomy, recent antibiotic usage, and prior vaccine reactions) and record any pre-existing conditions or signs and/or symptoms present in a subject prior to the start of the study in the eCRF. Treatment of any abnormality observed during this

examination has to be performed according to local medical practice outside this study or by referral to an appropriate health care provider.

5.6.2.6. Physical examination

Perform a physical examination of the subject, including assessment of body temperature, recording of height and body weight. Collected information needs to be recorded in the eCRF.

5.6.2.7. Urine pregnancy test

Female subjects of childbearing potential are to have a urine pregnancy test at the screening visit. The subject may only be enrolled if the pregnancy test is negative.

5.6.2.8. Blood sampling for safety and immune response assessments

As specified in the List of Study Procedures in Section 5.5 (Table 7), blood samples are e taken from all subjects at specific study visits. Refer to the Module on Biospecimen Management in the SPM for general handling of blood samples. The total volumes of whole blood to be taken at each pre-defined timepoint are given in Table 13.

<u>All subjects</u>

- A volume of at least 7 mL of whole blood should be drawn for analysis of safety parameters at each pre-defined timepoint. In addition a volume of at least 15 mL of whole blood should be drawn for additional screening analysis at screening visit 1.

<u>Subjects in the immunization groups only</u>

- A volume of at least 20 ml of whole blood (to provide approximately 10 ml of serum) should be drawn for analysis of humoral immune response at each pre-defined timepoint. After centrifugation, serum samples should be kept at –20°C or less until testing.
- A volume of 60 ml of whole blood should be drawn for analysis of cell-mediated immune (CMI) response at each pre-defined timepoint. Whole blood samples should be kept at room temperature (20 to 25°C) until they are transferred to the designated laboratory for further processing.
- A volume of at least 2 mL of whole blood should be drawn only for exploratory cytokine/chemokines analysis by Luminex. After centrifugation, ***serum*** samples should be kept at –70°C or less until testing. **(Amended 09 June 2011)**
- A volume of at least 12 mL of whole blood should be drawn for a multivariant analysis (systems biology approach using microarray analysis, deep sequencing). The blood should be stored at room temperature (20 to 25°C) until they are transferred to the designated laboratory for further processing.
- A volume of at least 10 mL of whole blood should be drawn for multiparameter flow cytometry for plasmablasts and B cell ELISPOT. The blood should be stored at room temperature (20 to 25°C) until they are transferred to the designated laboratory for further processing.

5.6.2.9. Urine sampling for safety assessment

Urine samples are taken from all subjects at specific study visits for safety assessment. Full details for urine sampling are provided in the Module on Biospecimen Management in the SPM accompanying this protocol.

5.6.2.10. Check and record concomitant medication/vaccination

Concomitant medication/vaccination must be recorded in the eCRF as described in Section 6.7. Refer also to Section 6.7.1 for details on the medication/vaccination forbidden and/or allowed during the study.

5.6.2.11. Recording of SAEs

Refer to Section 8.3 for procedures for the Investigator to record SAEs that are related to study participation or GSK concurrent medication/vaccination or any fatal SAE and to Section 8.4 for guidelines on how to report these SAEs to GSK Biologicals.

The subjects will be instructed to contact the investigator immediately should they feel unwell.

5.6.3. Procedures during the vaccination epoch (only for vaccinees)

Note that some of the procedures to be performed during the vaccination epoch (such as physical examination, blood sampling for safety and immune response assessments [humoral immune response, CMI, exploratory cytokine/chemokine analysis, systems biology, analysis of plasmablasts and and B cell ELISPOT], urine sampling for safety assessment, recording of concomitant medication/vaccination) are also performed at screening and are described in Section 5.6.2.

Physical examination (see Section 5.6.2.6) will be performed only if the subject indicates during questioning that there might be some underlying pathology(ies) or if deemed necessary by the Investigator or delegate. Information collected on physical examination does not need to be recorded in the eCRF.

5.6.3.1. Check and record intercurrent medical conditions

At each study visit subsequent to the first vaccination visit, it must be verified if the subject has experienced or is experiencing any intercurrent medical condition as described in 6.8. If it is the case, the condition(s) must be recorded in the eCRF.

5.6.3.2. Urine pregnancy test

Female subjects of childbearing potential are to have a urine pregnancy test prior to any study vaccine administration. The study vaccines may only be administered if the pregnancy test is negative.

5.6.3.3. Check contraindications, warnings and precautions to vaccination

Contraindications, warnings and precautions to vaccination are to be checked at the beginning of each vaccination visit. Refer to Section 6.5 and 6.6.

5.6.3.4. Assess pre-vaccination body temperature

The oral, axillary or tympanic body temperature of all subjects needs to be measured prior to any study vaccine administration. The preferred route for recording temperature in this study will be oral. If the subject has fever [fever is defined as temperature ≥ 38.0°C (100.4°F) on oral, axillary or tympanic setting, on the day of vaccination, the vaccination visit will be rescheduled within the interval for this visit (see Table 11).

5.6.3.5. Randomization

At the first vaccination visit, randomization will occur as explained in Section 5.2.

5.6.3.6. Treatment number assignment

At the first vaccination visit, the subject will be assigned a treatment number defining the treatment he/she will be receiving. The treatment number must be recorded in the eCRF at each vaccination visit.

5.6.3.7. Vaccination

- After completing the prerequisite procedures prior to vaccination, one dose of study vaccine will be administered intramuscularly (IM) in the deltoid of the non-dominant arm except for variances like tattoos, rashes, burns or other skin disorders subject to the discretion of the investigator when the dominant arm will be used (refer to Section 6.3 for detailed description of the vaccine administration procedure). If the investigator or delegate determines that the subject's health on the day of vaccination temporarily precludes vaccination, the visit will be rescheduled within the interval for this visit.
- The vaccinees will be observed closely for at least 30 minutes, with appropriate medical treatment readily available in case of anaphylaxis following the administration of vaccine.

5.6.3.8. Buccal swab sampling

At the first vaccination visit, a buccal swab is to be taken from all subjects for evaluation of the association between the human leukocyte antigen (HLA) type and the immune response to the study vaccines and malaria infection. Full details buccal swab sampling are provided in the Module on Biospecimen Management in the SPM accompanying this protocol.

5.6.3.9. Recording of AEs, SAEs and pregnancies

Refer to Section 8.3 for procedures for the Investigator to record AEs, SAEs and pregnancies. Refer to Section 8.4 for guidelines on how to report AEs, SAEs and pregnancies to GSK Biologicals, MVI and Crucell.

The subjects will be instructed to contact the investigator immediately should they feel unwell.

At each vaccination visit, diary cards and digital thermometer will be provided to the subjects to record body (oral) temperature and any solicited local/general AEs (i.e. on the day of vaccination and during the next 6 days) occurring after vaccination.

Completion of diary cards will be done with the investigator on days 1, 2, 3 and 6 after each vaccination. **(Amended 09 June 2011)**

The subjects will be instructed to return the completed diary card to the investigator 6 days after the vaccination visit. Any unreturned diary cards will be sought from the subjects through telephone call(s) or any other convenient procedure. The investigator will transcribe the collected information into the eCRF in English.

5.6.3.10. Standard neurological assessment

At the first vaccination visit (Day 0) and at Days 6 and 28 after the first vaccination visit, a standard neurological examination assessing central and peripheral nervous systems, motor and sensory functions will be performed using the neurological examination form. Refer to Appendix B for the neurological examination form.

5.6.4. Procedures during challenge epoch

Note that some of the procedures to be performed during the challenge epoch (such as physical examination, blood sampling for safety and immune response assessments [humoral immune response, CMI, exploratory cytokine/chemokine analysis and systems biology], recording of concomitant medication/vaccination and intercurrent medical conditions, recording of AEs, SAEs and pregnancies) are also performed during the other visits of the screening and vaccination epochs and are described in Sections 5.6.2 and 5.6.3.

Physical examination (see Section 5.6.2.6) will be performed only if the subject indicates during questioning that there might be some underlying pathology(ies) or if deemed necessary by the Investigator or delegate.

Information collected on physical examination does not need to be recorded in the eCRF.

5.6.4.1. Distribution of emergency notification card

Upon entry into the challenge phase of the study, all volunteers will be issued an emergency notification card containing the volunteer’s name, details on the exposure to malaria and a 24-hour emergency telephone contact numbers for study investigators.

5.6.4.2. Urine pregnancy test

Female subjects of childbearing potential are to have a urine pregnancy test prior to challenge. The challenge may only be performed if the pregnancy test is negative.

5.6.4.3. Blood sampling for PCR and smear

As specified in the List of Study Procedures in Section 5.5 (Table 9), blood samples are taken from all subjects at specific study visits. Blood samples for smears and PCR may be discontinued once the volunteer has 3 consecutive (separated by greater than 12 hours) negative smears following initial treatment. Refer to the Module on Biospecimen Management in the SPM for general handling of blood samples.

A volume of at least 2 mL of whole blood should be drawn from each subject for polymerase chain reaction (PCR) and malaria smear. Samples collected at WRAIR will be processed by the microscopy team per SSP. After finalization of slide results, the remaining blood will be frozen at -20°C until PCR assays are performed.

Samples collected at University of Maryland, CVD will be put into sterile EDTA (purple top) tubes. Blood smears (x 2) will be performed immediately, along with PCR (within 18 hours).

5.6.4.4. Check contraindications to challenge

Contraindications to challenge are to be checked at the beginning of the challenge visit (Study Day 77) as described in Section 5.4.3.1.

5.6.4.5. Challenge

After completing the prerequisite procedures prior to challenge, the subjects will be challenged with malaria as outlined in Section 5.4.3.3.

Each volunteer will be observed closely for at least 30 minutes following the challenge, after which blood pressure, pulse and oral temperature measurements will be taken. Any severe reactions will be reviewed by the PI.

After challenge, the subjects will be instructed to contact the investigator immediately should they manifest any signs or symptoms they feel unwell.

If a volunteer is found to be parasitemic, antimalarial treatment with oral chloroquine (see section 5.4.3.6) will be initiated immediately under direct observation of the physician.

5.6.4.6. Conclusion of the vaccination and challenge epochs

At the Clinic Visit 40, the investigator will complete the “End of challenge epoch” screen in the eCRF.

A primary analysis will be performed on all data collected up to Clinic Visit 40 (Study Day 105).

5.6.5. Procedures during follow-up epoch

Note that some of the procedures to be performed during the follow-up epoch (such as physical examination, blood sampling for safety and immune response assessments [humoral immune response and CMI], recording of concomitant medication/vaccination

and intercurrent medical conditions, recording of any SAEs and pregnancies) are also performed during the screening, vaccination and challenge epochs and are described in Sections 5.6.2, 5.6.3 and 5.6.4.

Physical examination (see Section 5.6.2.6) will be performed only if the subject indicates during questioning that there might be some underlying pathology(ies) or if deemed necessary by the Investigator or delegate. Information collected on physical examination does not need to be recorded in the eCRF.

5.6.5.1. Study conclusion

An analysis will be performed on additional safety data collected during the 6 months post-dose 3. At the end of the follow-up epoch, the investigator will complete the Study Conclusion screen in the eCRF.

5.7. Biological sample handling and analysis

Please refer to the SPM for details of biospecimen management (handling, storage and shipment).

Samples will not be labelled with information that directly identifies the subject but will be coded with the identification number for the subject (subject number).

Collected samples may be used in other assays, if the volunteer has provided consent, for test improvement or test development of analytical methods related to the study vaccines and its constituents or the disease under study to achieve a more reliable measurement of the vaccine response if all parties agree. Under these circumstances, additional testing on the samples may be performed outside the scope of this protocol if this is approved by the respective IRBs.

Volunteers will be required to provide consent at the time of trial screening in order for their blood to be used in future assays.

Information on further investigations and their rationale can be obtained from GSK Biologicals.

Any sample testing will be done in line with the consent of the individual subject.

Consent for HLA typing will be obtained at the time of trial screening and included in the Informed Consent Document. Any additional human genetic testing will require specific consent from the individual subjects and the ethics committee approval. Volunteers will also be required to provide consent for human immunodeficiency virus (HIV) testing at the time of trial screening.

If additional testing is performed, the marker priority ranking given in Section 5.7.4 may be changed.

Collected samples will be stored for up to 15 years (counting from when the last subject performed the last study visit), unless local rules, regulations or guidelines require

different timeframes or different procedures, which will then be in line with the subject consent. These extra requirements need to be communicated formally to and discussed and agreed with GSK Biologicals and partners.

5.7.1. Use of specified study materials

When materials are provided by GSK Biologicals, it is MANDATORY that all clinical samples (including serum samples) be collected and stored exclusively using those materials in the appropriate manner. The use of other materials could result in the exclusion of the subject from the ATP analysis (See Section 10.5 for the definition of study cohorts to be evaluated). The investigator must ensure that his/her personnel and the laboratory(ies) under his/her supervision comply with this requirement. However, when GSK Biologicals does not provide material for collecting and storing clinical samples, then appropriate materials from the investigator's site are to be used. Refer to the Module on Clinical Trial Supplies in the SPM.

5.7.2. Biological samples

Details of the quantity of biological sample to be taken at each timepoint during the study are provided in Table 13.

Table 13 Biological samples

Sample type	Quantity	Unit	Timepoint	Nr. subjects*
Blood	22	ml	Screening Visit 1 (Day -90 to -3)	168 (+12 to 18§)
	104	ml	Screening Visit 2 (Day -7 to -1)	168
	7	ml	Visit 3 (D0)	168
	14	ml	Visit 4 (D1), Visit 5 (D2), Visit 10 (D29), Visit 16 (D57), Visit 21 (D78)	168
	21	ml	Visit 7 (D6)	168
	74	ml	Visit 8 (D14)	168
	51	ml	Visit 9 (D28), Visit 15 (D56)	168
	31	ml	Visit 13 (D34), Visit 19 (D62)	168
	60	ml	Visit 14 (D42)	168
	101	ml	Visit 20 (D77)	168 (+12 to 18§)
	16	ml	Visit 22 (D82)	168 (+12 to 18§)
	2	ml	Visit 23 (D83), Visit 24 (D84), Visit 25 (D85), Visit 26 (D86), Visit 27 (D87), Visit 28 (D88), Visit 29 (D89), Visit 30 (D90), Visit 31 (D91), Visit 32 (D92)	168 (+12 to 18§)
	9	ml	Day of parasitemia (undetermined)	Undetermined
	2	ml	Visit 34 (D94), Visit 35 (D95), Visit 36 (D97), Visit 37 (D99), Visit 38 (D101), Visit 39 (D103)	168 (+12 to 18§)¶
	91	ml	Visit 40 (D105)	168 (+12 to 18§)
	87	ml	Visit 41 (D140)	168 (+12 to 18§)
	87	ml	Visit 42 (D236)	168 (+12 to 18§)
Urine (safety assessment)	-	-	Screening Visit 1 (D-90 to -3), Visit 6 (D6), Visit 9 (D28)	168
Urine (pregnancy test)**	-	-	Screening Visit 1 (D-90 to -3), Visit 3 (D0), Visit 9 (D28), Visit 15 (D56) and Visit 20 (D77)	Undetermined**
Buccal swab	-	-	Visit 3 (D0)	168

*Estimated number of subjects
§Four to six infectivity controls will be enrolled per challenge day for each of the three cohorts.
¶Blood collection may be discontinued in a volunteer who developed malaria, once he/she has 3 consecutive (separated by greater than 12 hours) negative smears following initial treatment.
**only from female subjects of childbearing potential
(Amended 09 June 2011)

5.7.3. Laboratory assays

Please refer to Appendix C for a detailed description of the assays performed in the study.

Analyses for this protocol include screening tests (antibody to HIV, HBV, HCV), safety testing (hemoglobin, WBC, PLT, creatinine, ALT and AST), serology (ELISA), CMI assays (ELISPOT and ICS), exploratory cytokine and chemokines analysis using Luminex, gene expression profiling (such as microarray and deep sequencing) and flow cytometry for plasmablast. HLA typing will be conducted from buccal swabs.

All assays will be performed using standardized and validated procedures at laboratories described in Table 14 to Table 19.

Table 14 Humoral Immunity (Antibody determination)

System	Component	Method	Kit / Manufacturer	Unit	Cut-off	Laboratory
Serum	*Plasmodium falciparum*.Circumsporozoite Protein.R32LR Ab.IgG	ELISA	In-house	EU/mL	0.5	GSK Biologicals†
Serum	Hepatitis B Virus.Surface Ab	ELISA	In-house	mIU/mL	10 *	GSK Biologicals†
Serum	Adenovirus serotype 35 Ad35 Abs	Neut. assay	Neut. assay	IC90	16	Crucell
Serum	*Plasmodium falciparum*.Circumsporozoite Protein. full-length & specific peptides other than repeat Ab IgG**	ELISA**	In-house	End-point titer	200	WRAIR
Serum	Anti-CS Isotype/Affinity**	ELISA**, QCM**	In-house	-	-	WRAIR
Serum	Anti-CS passive transfer**	Transgenic Parasites**	In-house	-	-	WRAIR

* seroprotective level
**exploratory
† or validated laboratory designated by GSK Biologicals
ELISA = Enzyme-linked Immunosorbent Assay
Neut. = Neutralizing
QCM = Quartz Crystal Microbalance technology Attana Cell 100 instrument

Table 15 Cell-Mediated Immunity (CMI)

System	Component	Challenge	Method	Unit	Laboratory
PBMC	CD4/CD8 CD40L(+)+Interleukin-2(+)+ Tumor Necrosis Factor alpha(+) +Interferon gamma(+)$	HBs and CS protein or derived peptides	ICS*	Number of cytokine(s) positive cells per 10^6 CD4/CD8 T cells	GSK Biologicals†
PBMC	Interferon gamma producing cells	CS derived peptides, spanning entire CS, including N-terminal	ELISPOT	Number of spot forming cells per 10^6 PBMC	Crucell
PBMC	Interferon gamma producing cells**	Ad35	ELISPOT**	Number of spot forming cells per 10^6 PBMC	Crucell
PBMC***	Plasmablasts**	-	FACS**	-	Emory University
PBMC	Antibody producing cells	HBs, CS protein or derived peptides and Ad35	ELISPOT	Number of spot forming cells per 10^6 PBMC	Emory University
Serum	Cytokines and chemokines**	-	30 plex cytokine/chemokine-Invitrogen (Luminex)**	MFV (mean fluorescent value)	WRAIR

† or validated laboratory designated by GSK Biologicals.
$ other exploratory markers of CMI may be assessed.
*or equivalent
**exploratory
***Fresh PBMC
ICS = Intracellular cytokine staining. PBMC = Peripheral blood mononuclear cells
PBMC = Peripheral blood mononuclear cells
(Amended 09 June 2011)

Table 16 Gene expression profiling as part of a systems biology approach

System	Component	Method	Kit / Manufacturer	Unit	Laboratory
PBMC	mRNA	Microarray*	Affymetrix GeneChip Human Genome U133 Plus 2.0 Array	Gene expression	Emory University
PBMC	mRNA	Deep sequencing*	Illumina Genome Analyzer *II* array	Gene expression	***Seattle Biomed***

*exploratory
PBMC = Peripheral blood mononuclear cells
(Amended 09 June 2011)

Table 17 Molecular Biology (PCR tests)

System	Component	Method	Unit	Laboratory
Blood	*P. falciparum* 18S	Real-time PCR	Ct value	WRAIR/UMB-CVD

Table 18 Haematology, Serum Chemistry, Urine tests

System	Component	Method	Scale	Laboratory
Urine	β-HCG*	N/A	Quantitative	Investigator's site
Whole blood	Hemoglobin<br>Leukocytes (White Blood Cells)<br>Platelets	N/A	Quantitative	Quest or LabCorp
Serum	Alanine Aminotransferase<br>Aspartate Aminotransferase<br>Creatinine	N/A	Quantitative	Quest or LabCorp

N/A: not applicable
* Urinary pregnancy test

Table 19 Urine analysis for safety

System	Component	Scale	Method	Unit	Laboratory
Urine	Protein<br>Erythrocytes (Red Blood Cells)	Quantitative	Test strip	NR	Quest or LabCorp

NR: not required

Collected samples will be used for purposes related to the quality assurance of data generated within the scope of this protocol, such as for maintenance of assays described in this protocol and comparison between analytical methods and/or laboratories.

The GSK Biologicals' clinical laboratories have established a Quality System supported by procedures. The activities of GSK Biologicals' clinical laboratories are audited regularly for quality assessment by an internal (sponsor-dependent) but laboratory-independent Quality Department.

5.7.4. Biological samples evaluation

5.7.4.1. Blood testing plan

Table 20 Summary of blood sampling time points and laboratory assays

Blood sampling timepoint		Component	No. subjects*	Priority ranking
Type of contact and timepoint	Sampling timepoint			
Visit 1 (D–90 to -3)	Pre(D-90 to -3)	**Safety analysis**		
		Hemoglobin, WBC, PLT, ALT, AST, creatinine	168(+12/18‡)	1
		**Additional screening parameters**		
		HIV, HCV, HBV	168(+12/18‡)	1
Visit 2 (D–7 to -1)	Pre(D-7 to -1)	**Investigational analysis**		
		Anti-CS (RT**) antibodies	168	1
		Anti-HBs antibodies	168	2
		Anti-Ad35 neutral. antibodies	168	3
		Anti-CS (FL** & specific peptides other then RT) antibodies	168	4
		Isotype and affinity of CS-specific antibodies	168	5
		CS-specific T-cells (ICS)	168	6
		HBs-specific T-cells (ICS)	168	7
		CS-specific T-cells (ELISPOT)	168	8
		Ad35-specific T-cells (ELISPOT)	168	9
		Exploratory cytokine/chemokines (Luminex)	168	10
		Microarray and deep sequencing	168	11
		Plasmablasts FACS & B cells ELISPOT	168	12
Visit 3 (D0)	Pre(D0)	**Safety analysis**		
		Hemoglobin, WBC, PLT, ALT, AST, creatinine	168	1
Visit 4 (D1)	PI(D1)	**Investigational analysis**		
		Exploratory cytokine/chemokines (Luminex)	168	1
		Microarray and deep sequencing	168	2
Visit 5 (D2)	PI(D2)	**Investigational analysis**		
		Exploratory cytokine/chemokines (Luminex)	168	1
		Microarray and deep sequencing	168	2
Visit 7 (D6)	PI(D6)	**Safety analysis**		
		Hemoglobin, WBC, PLT, ALT, AST, creatinine	168	1
		**Investigational analysis**		
		Exploratory cytokine/chemokines (Luminex)	168	1
		Microarray and deep sequencing	168	2
Visit 8 (D14)	PI(D14)	**Investigational analysis**		
		CS-specific T-cells (ICS)	168	1
		CS-specific T-cells (ELISPOT)	168	2
		N-terminal CS-specific T-cells (ELISPOT)	168	3
		Ad35-specific T-cells (ELISPOT)	168	4
		Exploratory cytokine (Luminex)	168	5
		Microarray and deep sequencing	168	6

Blood sampling timepoint				
**Type of contact and timepoint**	**Sampling timepoint**	**Component**	**No. subjects***	**Priority ranking**
Visit 9 (D28)	PI(D28)	**Safety analysis**		
		Hemoglobin, WBC, PLT, ALT, AST, creatinine	168	1
		**Investigational analysis**		
		Anti-CS (RT**) antibodies	168	1
		Anti-HBs antibodies	168	2
		Anti-Ad35 neutral. antibodies	168	3
		Anti-CS (FL** and specific peptides other then RT) antibodies	168	4
		Isotype and affinity of CS-specific antibodies	168	5
		Exploratory cytokine/chemokines (Luminex)	168	6
		Microarray and deep sequencing	168	7
		Plasmablasts FACS & B cells ELISPOT	168	8
Visit 10 (D29)	PII(D29)	**Investigational analysis**		
		Exploratory cytokine/chemokines (Luminex)	168	1
		Microarray and deep sequencing	168	2
Visit 13 (D34)	PII(D34)	**Safety analysis**		
		Hemoglobin, WBC, PLT, ALT, AST, creatinine	168	1
		**Investigational analysis**		
		Exploratory cytokine/chemokines (Luminex)	168	1
		Microarray and deep sequencing	168	2
		Plasmablasts FACS & B cells ELISPOT	168	3
Visit 14 (D42)	PII(D42)	**Investigational analysis**		
		CS-specific T-cells (ICS)	168	1
		HBs-specific T-cells (ICS)	168	2
		CS-specific T-cells (ELISPOT)	168	3
		Ad35-specific T-cells (ELISPOT)	168	4
Visit 15 (D56)	PII(D56)	**Safety analysis**		
		Hemoglobin, WBC, PLT, ALT, AST, creatinine	168	1
		**Investigational analysis**		
		Anti-CS (RT**) antibodies	168	1
		Anti-HBs antibodies	168	2
		Anti-Ad35 neutral. antibodies	168	3
		Anti-CS (FL** & specific peptides other then RT) antibodies	168	4
		Isotype and affinity of CS-specific antibodies	168	5
		Exploratory cytokine (Luminex)	168	6
		Microarray and deep sequencing	168	7
		Plasmablasts FACS & B cells ELISPOT	168	8
Visit 16 (D57)	***PIII(D57)***	***Safety analysis***		
		Exploratory cytokine (Luminex)	***168***	***1***
		Microarray and deep sequencing	***168***	***2***

Blood sampling timepoint: Type of contact and timepoint	Blood sampling timepoint: Sampling timepoint	Component	No. subjects*	Priority ranking
Visit 19 (D62)	PIII(D62)	**Safety analysis**		
		Hemoglobin, WBC, PLT, ALT, AST, creatinine	168	1
		**Investigational analysis**		
		Exploratory cytokine (Luminex)	168	1
		Microarray and deep sequencing	168	2
		Plasmablasts FACS & B cells ELISPOT	168	3
Visit 20 (D77)	PIII(D77)	**Safety analysis**		
		Hemoglobin, WBC, PLT, ALT, AST, creatinine	168(+12/18‡)	1
		**Investigational analysis**		
		Anti-CS (RT**) antibodies	168(+12/18[3])	1
		Anti-HBs antibodies	168(+12/18‡)	2
		Anti-Ad35 neutral. antibodies	168(+12/18‡)	3
		Anti-CS (FL**& specific peptides other then RT) antibodies	168(+12/18‡)	4
		Isotype and affinity of CS-specific antibodies	168(+12/18‡)	5
		CS-specific T-cells (ICS)	168(+12/18‡)	6
		HBs-specific T-cells (ICS)	168(+12/18‡)	7
		CS-specific T-cells (ELISPOT)	168(+12/18‡)	8
		Ad35-specific T-cells (ELISPOT)	168(+12/18‡)	9
		Exploratory cytokine/chemokines (Luminex)	168(+12/18‡)	10
		Microarray and deep sequencing	168(+12/18‡)	11
Visit 21 (D78)	PIII(D78)	**Investigational analysis**		
		Exploratory cytokine/chemokines (Luminex)	168(+12/18‡)	1
		Microarray and deep sequencing	168(+12/18‡)	2
Visit 22 (D82)	PIII(D82)	Smear and PCR	168(+12/18‡)	1
		**Investigational analysis**		
		Exploratory cytokine/chemokines (Luminex)	168(+12/18‡)	1
		Microarray and deep sequencing	168(+12/18‡)	2
Visit 23 (D83)<br>Visit 24 (D84)<br>Visit 25 (D85)<br>Visit 26 (D86)<br>Visit 27 (D87)<br>Visit 28 (D88)<br>Visit 29 (D89)<br>Visit 30 (D90)<br>Visit 31 (D91)<br>Visit 32 (D92)	PIII(D83)<br>PIII(D84)<br>PIII(D85)<br>PIII(D86)<br>PIII(D87)<br>PIII(D88)<br>PIII(D89)<br>PIII(D90)<br>PIII(D91)<br>PIII(D92)	Smear and PCR	168(+12/18‡)	1
Day of parasitemia	PIII(DoP)	Smear and PCR	168(+12/18‡)	1
		**Safety analysis**		
		Hemoglobin, WBC, PLT, ALT, AST, creatinine	§	1

Blood sampling timepoint		Component	No. subjects*	Priority ranking
Type of contact and timepoint	Sampling timepoint			
Visit 34 (D94)<br>Visit 35 (D95)<br>Visit 36 (D97)<br>Visit 37 (D99)<br>Visit 38 (D101)<br>Visit 39 (D103)	PIII(D94)<br>PIII(D95)<br>PIII(D97)<br>PIII(D99)<br>PIII(D101)<br>PIII(D103)	Smear and PCR	168(+12/18‡)¶	1

Visit 40 (D105)	PIII(D105)	Smear and PCR	168(+12/18‡)	1
		**Safety analysis**		
		Hemoglobin, WBC, PLT, ALT, AST, creatinine	168(+12/18‡)	1
		**Investigational analysis**		
		Anti-CS (RT**) antibodies	168(+12/18‡)	1
		Anti-HBs antibodies	168(+12/18‡)	2
		Anti-Ad35 neutral. antibodies	168(+12/18‡)	3
		Anti-CS (FL**& specific peptides other then RT) antibodies	168(+12/18‡)	4
		Isotype and affinity of CS-specific antibodies	168(+12/18‡)	5
		CS-specific T-cells (ICS)	168(+12/18‡)	6
		HBs-specific T-cells (ICS)	168(+12/18‡)	7
		CS-specific T-cells (ELISPOT)	168(+12/18‡)	8
		Ad35-specific T-cells (ELISPOT)	168(+12/18‡)	9
		Exploratory cytokine/chemokines (Luminex)	168(+12/18‡)	10

Visit 41 (D140)	PIII(D140)	**Safety analysis**		
		Hemoglobin, WBC, PLT, ALT, AST, creatinine	168(+12/18‡)	1
		**Investigational analysis**		
		Anti-CS (RT**) antibodies	168(+12/18‡)	1
		Anti-HBs antibodies	168(+12/18‡)	2
		Anti-Ad35 neutral. antibodies	168(+12/18‡)	3
		Anti-CS (FL**& specific peptides other then RT) antibodies	168(+12/18‡)	4
		Isotype and affinity of CS-specific antibodies	168(+12/18‡)	5
		CS-specific T-cells (ICS)	168(+12/18‡)	6
		HBs-specific T-cells (ICS)	168(+12/18‡)	7
		CS-specific T-cells (ELISPOT)	168(+12/18‡)	8
		Ad35-specific T-cells (ELISPOT)	168(+12/18‡)	9

Blood sampling timepoint		Component	No. subjects*	Priority ranking
Type of contact and timepoint	Sampling timepoint			
Visit 42 (D236)	PIII(D236)	**Safety analysis**		
		Hemoglobin, WBC, PLT, ALT, AST, creatinine	168(+12/18‡)	1
		**Investigational analysis**		
		Anti-CS (RT**) antibodies	168(+12/18‡)	
		Anti-HBs antibodies	168(+12/18‡)	
		Anti-Ad35 neutral. antibodies	168(+12/18‡)	
		Anti-CS (FL**& specific peptides other then RT) antibodies	168(+12/18‡)	
		Isotype and affinity of CS-specific antibodies	168(+12/18‡)	
		CS-specific T-cells (ICS)	168(+12/18‡)	
		HBs-specific T-cells (ICS)	168(+12/18‡)	
		CS-specific T-cells (ELISPOT)	168(+12/18‡)	
		Ad35-specific T-cells (ELISPOT)	168(+12/18‡)	

ICS: Intracellular Cytokine Staining. DoP: Day of parasitemia. D = Day
*Estimated number of subjects
§Day of parasitemia and number of parasitemic individuals is undetermined
‡ Four to six infectivity controls will be enrolled per challenge day for each of the three cohorts.
**RT: repeat/FL: full length
¶For volunteers who develop malaria: blood smears and PCR may be discontinued in a volunteer who developed malaria, once he/she has 3 consecutive (separated by greater than 12 hours) negative smears following initial treatment.
(Amended 09 June 2011)

In case of insufficient blood sample volume to perform assays for all antibodies, the samples will be analysed according to priority ranking provided in Table 20.

Other assays to investigate vaccine safety and/or vaccine induced anti-malaria, hepatitis B and/or adenovirus immune response may be performed on stored serum or cell samples remaining after protocol-specific laboratory testing is completed.

5.7.5. Immunological correlates of protection

No immunological correlate of protection against malaria has been demonstrated so far. The association between high humoral and T-cell anti-CS responses and protection against infection in the sporozoite challenge model has been shown but no protective threshold is identified. In this study, the relationship between immune response generated against malaria antigens and protection will be investigated.

For the hepatitis B antigen, the conventional correlate of protection is anti-HBs antibody titers above 10 mIU/mL [Hadler, 1986].

6. STUDY VACCINES AND ADMINISTRATION

6.1. Description of study vaccines

The candidate RTS,S/$AS01_B$ vaccine to be used has been developed and manufactured by GSK Biologicals.

The candidate Ad35.CS.01 vaccine to be used has been manufactured by Crucell Holland BV (GMP facility), the Netherlands. The final Ad35.CS.01 vaccine has been formulated and aseptically filled at SAFC Pharma, in the United States according to applicable cGMP.

The Quality Control Standards and Requirements for each candidate vaccine are described in separate Quality Assurance documents (e.g. release protocols, certificate of analysis) and the required approvals have been obtained.

The vaccines are labelled and packed according to applicable regulatory requirements.

The formulation of the study vaccines is presented in Table 21**.**

Table 21 Study vaccines

Treatment name	Vaccine/ product name	Formulation	Presentation	***Injectable* volume**	Number of doses
Ad35.CS.01	Ad35.CS.01	2x10^{11} Ad35.CS.01 vp/mL, Tris, NaCl, $MgCl_2$, PolySorbate-80, Sucrose, EDTA, Histidine, Ethanol	Clear to slightly opalescent and colourless solution with no visible particles in type I glass vial	0.5 ml***[1, 2]***	1
	Diluent	***Tris, MgCl2, NaCl, Sucrose, PolySorbate-80, Ethanol***	***Clear liquid solution in type I glass vial***		***1***
RTS,S/$AS01_B$	RTS,S	RTS,S antigen: 50 µg RTS,S with sucrose as cryoprotectant	RTS,S antigen: Lyophilized pellet in single dose vial to be reconstituted with $AS01_B$ adjuvant in liquid form	0.5 ml***[3]***	2/3
	$AS01_B$	$AS01_B$ adjuvant: 50 microgrammes of MPL and 50 microgrammes of Stimulon QS21 (a triterpene glycoside purified from the bark of Quillaja saponaria) with liposomes	$AS01_B$ adjuvant: liquid solution in monodose glass vial		

[1] Volume after dilution
[2] One dose of 0.5 ml after dilution will contain 5x10^{10} Ad35.CS.01 vp.
[3] Volume after reconstitution
Note: Due to ***potential visible*** differences between the reconstituted RTS,S/$AS01_B$ and the Ad35.CS.01 vaccine, dedicated unblinded staff in each of the investigational sites will be accountable for the reconstitution and administration ***of the vaccine*** to a subject. **(Amended 09 June 2011)**

6.2. Storage and handling of study vaccines

All study vaccines to be administered to the subjects must be stored in a safe and locked place with no access by unauthorised personnel.

6.2.1. RTS,S/AS01$_B$ (0.5 mL dose)

The RTS,S/AS01$_B$ vaccine must be stored at the defined temperature range (i.e. +2 to +8°C/36°F to 46°F). Please refer to the Module on Clinical Trial Supplies in the SPM for more details on storage of the study vaccines. The storage temperature of the vaccine will be monitored daily with temperature monitoring device(s) (at a minimum calibrated) and will be recorded as specified in the SPM.

The storage conditions will be assessed during pre-study activities under the responsibility of the sponsor study contact.

Any temperature deviation outside the range 0 to 8°C/32 to 46°F must be reported to the sponsor as soon as detected. Following an exposure to such a temperature deviation, vaccines will not be used until approval has been given by the Sponsor.

In case of temperature deviation between 0 and 2°C/32 to 36°F the impacted study vaccines can still be administered, but the site must take adequate actions to go back to the defined range +2 to +8°C/36 to 46°F and avoid re-occurrence of such a temperature deviation.

Refer to the Module on Clinical Trial Supplies in the SPM for details and instructions on the on the Temperature deviation process, packaging and accountability of the RTS,S/AS01$_B$ vaccines.

6.2.2. Ad35.CS.01 (0.5 mL dose)

The Ad35.CS.01 vaccine must be stored at the defined temperature range (i.e. ≤-65°C/≤-85°F). ***The diluent must be stored at 2-8°C/36 to 46°F.*** Please refer to the Module on Clinical Trial Supplies in the SPM for more details on storage of the study vaccines. The storage temperature of the vaccine will be monitored daily with temperature monitoring device(s) (at a minimum calibrated) and will be recorded as specified in the SPM.

The storage conditions will be assessed during pre-study activities under the responsibility of the sponsor study contact.

Any temperature deviation >-65°C/-85°F ***for the Ad35.CS.01 vaccine or outside the range 2 to 8°C/36 to 46°F for the diluent*** must be reported to ***the sponsor*** as soon as detected. ***Once the sponsor becomes aware of a temperature deviation, he must inform Crucell IMMEDIATELY.*** Following an exposure to such a temperature deviation, ***the Ad35.CS.01*** vaccine ***and the diluent*** will not be used until approval has been given by Crucell ***via the sponsor***.

Refer to the Module on Clinical Trial Supplies in the SPM for details and instructions on the Temperature deviation process, packaging and accountability of the Ad35.CS.01 vaccine ***and diluent***.

(Amended 09 June 2011)

6.3. Dosage and administration of study vaccines

Table 22 Dosage and administration

Type of contact and timepoint	Dose	Treatment Group	Vaccine	Route[1]	Site[2]	Side[3]
visit 3 (Day 0)	1	ARR	Ad35.CS.01	IM	D	N-D
	1	RRR	RTS,S/AS01$_B$	IM	D	N-D
visit 9 (Day 28)	2	ARR , RRR	RTS,S/AS01$_B$	IM	D	N-D
visit 15 (Day 56)	3	ARR , RRR	RTS,S/AS01$_B$	IM	D	N-D

[1]Intramuscular (IM)
[2]Deltoid (D)
[3]Non-dominant (N-D) except for variances like tattoos, rashes, burns or other skin disorders subject to the discretion of the investigator.

6.3.1. Injection technique

Intramuscular injections are administered in the deltoid of the non-dominant arm except for variances like tattoos, rashes, burns or other skin disorders subject to the discretion of the investigator when the dominant arm will be used for injection. **The buttock should not be used for administration of vaccines** because of the potential risk of injury to the sciatic nerve and the risk of decreased immunogenicity because of inadvertent subcutaneous injection or injection into deep fat tissue.

For all intramuscular injections, the needle should be long enough to reach the muscle mass and prevent vaccine from seeping into subcutaneous tissue, but not so long as to involve underlying nerves, blood vessels or bone. Vaccinators should be familiar with the anatomy of the area into which they are injecting vaccine. Although it is recommended to follow the guidelines provided below, an individual decision on needle size and site of injection must be made for each person on the basis of age, and the size of the muscle.

Intramuscular injections should be administered with caution to subjects with thrombocytopenia or a bleeding disorder since bleeding may occur following an intramuscular administration to these subjects. Firm pressure should be applied to the injection site (without rubbing) for at least two minutes.

6.3.2. Injection instructions

6.3.2.1. RTS,S/AS01$_B$ (0.5 mL dose)

RTS,S/AS01$_B$ will be supplied such that the reconstituted vaccine volume will provide a 0.5 mL dose.

Disinfect top of vaccine vial (pellet) and adjuvant vial with alcohol swabs and let dry. Withdraw the content of the adjuvant vial in a syringe and inject adjuvant into the vial of

lyophilized antigen. The pellet is then dissolved by gently shaking the vial. Wait for 1 minute to ensure complete dissolution of vial content before withdrawing the content of the vial (0.5 mL). The needle should be changed before injection.

One dose (0.5 mL) of the reconstituted vaccine should be administered by slow IM injection into the deltoid muscle of the non-dominant arm except for variances like tattoos, rashes, burns or other skin disorders subject to the discretion of the investigator. Vaccine should be injected within 4 hours of reconstitution (storage at +2°C to +8°C/36°F to 46°F).

6.3.2.2. Ad35.CS.01 (0.5 mL dose)

The Ad35.CS.01 vaccine will be supplied ***as a*** single use, ***3 mL*** clear ***type I*** glass vial containing ***0.7 mL vaccine (0.5 mL extractable). The diluent to be used to dilute the Ad35.CS.01 vaccine to the intended dose for the clinical study is supplied as a single use, 3 mL clear type I glass vial containing 1.4 mL solution (1.0 mL extractable).***

The Ad35.CS.01 vaccine storage condition is ≤-65°C/-85°F. The Ad35.CS.01 vaccine will be thawed at room temperature (15-25°C) prior to dose preparation. When thawed, the liquid material appears as a clear to slightly opalescent solution.

Each dose (per study protocol) of the vaccine (0.5 mL) will be administered with a sterile, disposable syringe and needle by IM injection into the deltoid muscle of the non-dominant arm except for variances like tattoos, rashes, burns or other skin disorders subject to the discretion of the investigator, using a fresh needle.

Detailed instructions for the preparation, dilution and dispensation of the assigned dose of the investigational product will be provided separately in the Pharmacy Manual.

(Amended 07 June 2001)

6.4. Replacement of unusable vaccine doses

Additional vaccine doses will be provided to replace those that are unusable (see the Module on Clinical Trial Supplies in the SPM for details).

In addition to the vaccine doses provided for the planned number of subjects (including over-randomization when applicable), at least 10% additional doses will be supplied to replace those that are unusable.

The investigator will use the SBIR to obtain the replacement vial number. The replacement numbers will be allocated by dose. The system will ensure, in a blinded manner, that the replacement vial matches the formulation the subject was assigned to by randomisation.

6.5. Contraindications to vaccination

6.5.1. Absolute contraindications to vaccination

The following events constitute absolute contraindications to administration of study vaccines. If any of these events occur during the study, the subject must not receive the vaccine dose but should continue other study procedures at the discretion of the investigator (see Section 9.2).

- Anaphylaxis following the administration of vaccines.
- Pregnancy (see Section 8.2.1).
- Any confirmed or suspected immunosuppressive or immunodeficient condition, including human immunodeficiency virus (HIV) infection.

6.5.2. Indications for deferral of vaccination

The following events constitute contraindications to administration of study vaccines at that point in time; if any of these events occur at the time scheduled for vaccination, the subject may be vaccinated at a later date, within the time window specified in the protocol (see Table 11), or the subject may be withdrawn at the discretion of the investigator (see Section 9.2).

- Acute disease and/or fever at the time of vaccination.
  - Acute disease is defined as the presence of a moderate or severe illness with or without fever. Subjects with a minor illness (such as mild diarrhoea, mild upper respiratory infection) without fever can be administered all vaccines.
  - Fever is defined as temperature ≥ 38.0°C (100.4°F) on oral, axillary or tympanic setting. The preferred route for recording temperature in this study will be oral.

6.6. Warnings and precautions

As with all injectable vaccines, appropriate medical treatment and supervision should always be readily available in case of a rare anaphylactic reaction following the administration of the study vaccines.

Intramuscular vaccines should be administered with caution to subjects with thrombocytopenia or a bleeding disorder since bleeding may occur following an intramuscular administration to these subjects.

The study vaccines should under no circumstances be administered intravascularly or intradermally.

6.7. Concomitant medication/vaccination

At each study visit/contact, the investigator should question the subject about any medication taken and vaccination received by the subject.

All concomitant medication/vaccination, with the exception of vitamins and/or dietary supplements, are to be recorded in the eCRF. This also applies to concomitant medication administered prophylactically in anticipation of reaction to the vaccination and any medication intended to treat an AE.

A prophylactic medication is a medication administered in the absence of ANY symptom and in anticipation of a reaction to the vaccination (e.g. an anti-pyretic is considered to be prophylactic when it is given in the absence of fever and any other symptom, to prevent fever from occurring [fever is defined as temperature ≥ 38.0°C (100.4°F) on oral, axillary or tympanic setting]).

Similarly, concomitant medication administered for the treatment of a SAE, at any time, must be recorded on the SAE screens in the eCRF. Refer to Section 8.1.2 for the definition of a SAE.

6.7.1. Medications/products that may lead to the elimination of a subject from ATP analyses

The following criteria should be checked at each visit subsequent to the first vaccination visit. If any become applicable during the study, it will not require withdrawal of the subject from the study but may determine a subject's evaluability in the according-to-protocol (ATP) analysis. See Section 10.5 for definition of study cohorts to be evaluated.

- Use of any investigational or non-registered product (drug or vaccine) other than the study vaccines during the study period.
- Chronic administration (defined as more than 14 days) of immunosuppressants or other immune-modifying drugs during the study period. For corticosteroids, this will mean prednisone <20 mg/kg/day, or equivalent. Inhaled and topical steroids are allowed.
- Administration of a vaccine not foreseen by the study protocol during the period starting from 7 days before each vaccination and ending 7 days after each vaccination.
- Administration of immunoglobulins and/or any blood products during the study period.
- Drug and alcohol abuse.

A detailed, comprehensive list of reasons for elimination from ATP analyses will be established at the time of data cleaning.

6.7.2. Time window for recording concomitant medication/vaccination in the eCRF

All concomitant medications, with the exception of vitamins and/or dietary supplements, administered at ANY time during the period starting with the ***first screening visit*** and ending 6 months after last dose of study vaccine must be recorded in the eCRF. **(Amended 09 June 2011)**

Any vaccine not foreseen in the study protocol administered in the period beginning 30 days preceding the first dose of study vaccine and ending 6 months after last dose of study vaccine must be recorded in the eCRF.

6.8. Intercurrent medical conditions that may lead to elimination from an ATP cohort

Subjects may be eliminated from the ATP cohort for immunogenicity if, during the study, they incur a condition that has the capability of altering their immune response or are confirmed to have an immunodeficiency condition.

7. HEALTH ECONOMICS

Not applicable.

8. ADVERSE EVENTS AND SERIOUS ADVERSE EVENTS

The investigator or site staff is/are responsible during the study for the detection and documentation of events meeting the criteria and definition of an adverse event (AE) or serious adverse event (SAE) as provided in this protocol.

Each subject will be instructed to contact the investigator immediately should the subject feel unwell.

8.1. Safety definitions

8.1.1. Definition of an adverse event

An AE is any untoward medical occurrence in a clinical investigation subject, temporally associated with the use of a medicinal product, whether or not considered related to the medicinal product.

An AE can therefore be any unfavourable and unintended sign (including an abnormal laboratory finding), symptom, or disease (new or exacerbated) temporally associated with the use of a medicinal product.

Examples of an AE include:

- Significant or unexpected worsening or exacerbation of the condition/indication under study.
- Exacerbation of a chronic or intermittent pre-existing condition including either an increase in frequency and/or intensity of the condition.
- New conditions detected or diagnosed after investigational product administration even though they may have been present prior to the start of the study.
- Signs, symptoms, or the clinical sequelae of a suspected interaction.

- Signs, symptoms, or the clinical sequelae of a suspected overdose of either investigational product or a concurrent medication (overdose per se should not be reported as an AE/SAE).
- Signs, symptoms temporally associated with vaccine administration.
- Significant failure of expected pharmacological or biological action.

Examples of an AE DO NOT include:

- Medical or surgical procedures (e.g. endoscopy, appendectomy); the condition that leads to the procedure is an AE.
- Situations where an untoward medical occurrence did not occur (e.g. social and/or convenience admission to a hospital, admission for routine examination).
- Anticipated day-to-day fluctuations of pre-existing disease(s) or condition(s) present or detected at the start of the study that do not worsen.

AEs may include pre- or post-treatment events that occur as a result of protocol-mandated procedures (i.e. invasive procedures, modification of subject's previous therapeutic regimen).

Note: AEs to be recorded as endpoints (solicited AEs) are described in Section 8.1.3. All other AEs will be recorded as UNSOLICITED AEs.

Example of events to be recorded in the medical history section of the eCRF:

- Pre-existing conditions or signs and/or symptoms present in a subject prior to the start of the study (i.e. prior to the first study vaccination).

8.1.2. Definition of a serious adverse event

A serious adverse event (SAE) is any untoward medical occurrence that:

a. Results in death.

b. Is life-threatening.

   Note: The term 'life-threatening' in the definition of 'serious' refers to an event in which the subject was at risk of death at the time of the event. It does not refer to an event, which hypothetically might have caused death, had it been more severe.

c. Requires hospitalization or prolongation of existing hospitalization.

   Note: In general, hospitalization signifies that the subject has been admitted at the hospital or emergency ward for observation and/or treatment that would not have been appropriate in the physician's office or out-patient setting. Complications that occur during hospitalization are also considered AEs. If a complication prolongs hospitalization or fulfils any other serious criteria, the event will also be considered serious. When in doubt as to whether 'hospitalization' occurred or was necessary, the AE should be considered serious.

Hospitalization for elective treatment of a pre-existing condition (known/diagnosed prior to informed consent signature) that did not worsen from baseline is NOT considered an AE.

d. Results in disability/incapacity, or

Note: The term disability means a substantial disruption of a person's ability to conduct normal life functions. This definition is not intended to include experiences of relatively minor medical significance such as uncomplicated headache, nausea, vomiting, diarrhoea, influenza like illness, and accidental trauma (e.g. sprained ankle) which may interfere or prevent everyday life functions but do not constitute a substantial disruption.

e. Is a congenital anomaly/birth defect in the offspring of a study subject.

Medical or scientific judgement should be exercised in deciding whether reporting is appropriate in other situations, such as important medical events that may not be immediately life-threatening or result in death or hospitalization but may jeopardise the subject or may require medical or surgical intervention to prevent one of the other outcomes listed in the above definition. These should also be considered serious. Examples of such events are invasive or malignant cancers, intensive treatment in an emergency room or at home for allergic bronchospasm, blood dyscrasias or convulsions that do not result in hospitalization.

8.1.3. Solicited adverse events

The following local (injection-site) adverse events will be solicited:

Table 23 Solicited local adverse events

Pain at injection site
Redness at injection site
Swelling at injection site

The following general adverse events will be solicited:

Table 24 Solicited general adverse events

Fatigue
Fever
Gastrointestinal symptoms †
Headache
Chills

†Gastrointestinal symptoms include nausea, vomiting, diarrhoea and/or abdominal pain.

Note: Temperature will be recorded once per day. Should additional temperature measurements be performed, the highest temperature will be recorded in the eCRF.

8.1.4. Clinical laboratory parameters and other abnormal assessments qualifying as adverse events or serious adverse events

Abnormal laboratory findings (e.g. clinical chemistry, haematology, urinalysis) or other abnormal assessments that are judged by the investigator to be clinically significant will be recorded as AEs or SAEs if they meet the definition of an AE, as defined in Section 8.1.1 or of a SAE, as defined in Section 8.1.2. Clinically significant abnormal laboratory findings or other abnormal assessments that are detected during the study or are present at baseline and significantly worsen following the start of the study will be reported as AEs or SAEs.

The investigator will exercise his or her medical and scientific judgement in deciding whether an abnormal laboratory finding or other abnormal assessment is clinically significant.

8.1.5. AEs of specific interest

8.1.5.1. Potential immune-mediated diseases

Potential immune-mediated diseases (pIMDs) are a subset of AEs that include autoimmune diseases and other inflammatory and/or neurologic disorders of interest which may or may not have an autoimmune aetiology. AEs that need to be recorded and reported as pIMDs include those listed in the table below.

However, the investigator will exercise his/her medical and scientific judgement in deciding whether other immune-mediated diseases have an autoimmune origin (i.e. pathophysiology involving systemic or organ-specific pathogenic autoantibodies) and should also be recorded as a pIMD.

Table 25 List of potential immune-mediated diseases

Neuroinflammatory disorders	Musculoskeletal disorders	Skin disorders
• Cranial nerve disorders, including paralyses/paresis (e.g. Bell's palsy), and neuritis (e.g. optic neuritis)<br>• Multiple sclerosis (including variants)<br>• Transverse myelitis<br>• Guillain-Barré syndrome, (including Miller Fisher syndrome and other variants)<br>• Other demyelinating diseases (including acute disseminated encephalomyelitis)<br>• Myasthenia gravis (including Lambert-Eaton myasthenic syndrome)<br>• Non-infectious encephalitis/ encephalomyelitis<br>• Neuritis (including peripheral neuropathies)	• Systemic lupus erythematosus<br>• Scleroderma (including, CREST syndrome and morphoea)<br>• Systemic sclerosis<br>• Dermatomyositis<br>• Polymyositis<br>• Antisynthetase syndrome<br>• Rheumatoid arthritis,<br>• Juvenile chronic arthritis, (including Still's disease)<br>• Polymyalgia rheumatica<br>• Reactive arthritis<br>• Psoriatic arthropathy<br>• Ankylosing spondylitis<br>• Relapsing polychondritis<br>• Mixed connective tissue disorder	• Psoriasis<br>• Vitiligo<br>• Raynaud's phenomenon<br>• Erythema nodosum<br>• Autoimmune bullous skin diseases (including pemphigus, pemphigoid and dermatitis herpetiformis)<br>• Cutaneous lupus erythematosus<br>• Alopecia areata<br>• Lichen planus<br>• Sweet's syndrome
**Liver disorders**	**Gastrointestinal disorders**	**Metabolic diseases**
• Autoimmune hepatitis<br>• Primary biliary cirrhosis<br>• Primary sclerosing cholangitis<br>• Autoimmune cholangitis.	• Crohn's disease<br>• Ulcerative colitis<br>• Ulcerative proctitis<br>• Celiac disease	• Autoimmune thyroiditis (including Hashimoto thyroiditis)<br>• Grave's or Basedow's disease<br>• Diabetes mellitus type I<br>• Addison's disease

Vasculitides	Others
• Large vessels vasculitis including: giant cell arteritis such as Takayasu's arteritis and temporal arteritis.<br>• Medium sized and/or small vessels vasculitis including: polyarteritis nodosa, Kawasaki's disease, microscopic polyangiitis, Wegener's granulomatosis, Churg–Strauss syndrome, thromboangiitis obliterans (Buerger's disease), necrotizing vasculitis, allergic granulomatous angiitis, Henoch-Schonlein purpura, anti-neutrophil cytoplasmic antibody positive vasculitis, Behcet's syndrome, leukocytoclastic vasculitis.<br>• Vasculitides secondary to other immune mediated diseases such as lupus vasculitis and rheumatoid vasculitis.	• Autoimmune hemolytic anemia<br>• Autoimmune thrombocytopenias<br>• Antiphospholipid syndrome<br>• Pernicious anemia<br>• Autoimmune glomerulonephritis (including IgA nephropathy, glomerulonephritis rapidly progressive, membranous glomerulonephritis, membranoproliferative glomerulonephritis, and mesangioproliferative glomerulonephritis)<br>• Uveitis<br>• Autoimmune myocarditis/cardiomyopathy<br>• Sarcoidosis<br>• Stevens-johnson syndrome<br>• Sjögren's syndrome<br>• Idiopathic pulmonary fibrosis<br>• Goodpasture syndrome

When there is enough evidence to make any of the above diagnoses, the AE must be reported as a pIMD. Symptoms, signs or conditions which might (or might not) represent the above diagnoses, should be recorded and reported as AEs but not as pIMDs until the final or definitive diagnosis has been determined, and alternative diagnoses have been eliminated or shown to be less likely.

In order to facilitate the documentation of pIMDs in the eCRF, a pIMD standard questionnaire and a list of preferred terms (PTs) and PT codes corresponding to the above diagnoses will be available to investigators at study start.

8.1.5.2. New onset of neurological diseases

New onset of neurological diseases will be reported as AEs/SAEs in the eCRF or SAE Report screens, as applicable.

8.2. Events or outcomes not qualifying as adverse events or serious adverse events

8.2.1. Pregnancy

Any female subjects that are pregnant or lactating at the time of vaccination must not receive additional doses of study vaccines and must not be challenged with parasitized mosquitoes but may continue other study procedures at the discretion of the investigator.

While pregnancy itself is not considered an AE or SAE, any pregnancy complication or elective termination of a pregnancy for medical reasons will be recorded as an AE or a SAE, as described in Section 8.1.1 and 8.1.2, and will be followed as described in Section 8.5.

A spontaneous abortion is always considered to be a SAE and will be reported as described in Section 8.4. Furthermore, any SAE occurring as a result of a post-study pregnancy AND considered by the investigator to be reasonably related in time to the receipt of the investigational product will be reported to GSK Biologicals and MVI as described in Section 8.4. While the investigator is not obligated to actively seek this information from former study participants, he/she may learn of a pregnancy through spontaneous reporting.

8.3. Detecting and recording adverse events, serious adverse events and pregnancies

8.3.1. Time period for detecting and recording adverse events, serious adverse events and pregnancies

All AEs starting at the first receipt of study vaccine up to 30 days following sporozoite challenge must be recorded into the Adverse Event screen in the subject's eCRF, irrespective of intensity or whether or not they are considered vaccination-related.

The standard time period for collecting and recording SAEs will begin at the first receipt of study vaccines and will end 180 days (approximately 6 months) following administration of the last dose of study vaccines for each subject. See Section 8.4 for instructions on reporting and recording SAEs.

In addition to the above-mentioned reporting requirements and in order to fulfill international reporting obligations, SAEs that are related to study participation (e.g. protocol-mandated procedures, invasive tests, a change from existing therapy) or are

related to a concurrent GSK medication/vaccine or any fatal SAE will be collected and recorded from the time the subject consents to participate in the study until he/she is discharged.

The standard time period for collecting and recording pregnancies will begin at the first receipt of study vaccines and will end 180 days (approximately 6 months) following administration of the last dose of study vaccine. See section 8.4 for instructions on reporting of pregnancies.

The standard time period for collecting and recording of pIMDs will begin at the first receipt of study vaccines will end 180 days (approximately 6 months) following administration of the last dose of study vaccines. See section 8.4 for instructions on reporting of pIMDs.

An overview of the protocol-required reporting periods for adverse events, serious adverse events and pregnancies is given in Table 26.

Table 26 Reporting periods for adverse events, serious adverse events and pregnancies

Study activity	Pre-V1	V1	6 days post-V1	27 days post-V1	V2	6 days post V2	27 days post-V2	V3	6 days post V3	Challenge	29 days post-V3	29 days post-challenge	Study Conclusion
**Study day**	**-90 to -1**	**0**	**6**	**27**	**28**	**34**	**55**	**56**	**62**	**77**	**85**	**107**	**236**
Reporting of solicited local and general AEs													
Reporting of unsolicited AEs													
Reporting of SAEs													
Reporting of SAEs related to study participation or GSK concurrent medication/vaccination and any fatal SAE													
Reporting of pregnancies													
Reporting of pIMDs													

Pre-V: pre-vaccination; V: vaccination; Post-V: post-vaccination

A post-study AE/SAE is defined as any event that occurs outside of the AE/SAE reporting period defined in Table 26. Investigators are not obligated to actively seek AEs or SAEs in former study participants. However, if the investigator learns of any SAE at any time after a subject has been discharged from the study, and he/she considers the event reasonably related to the investigational product, the investigator will promptly notify the Study Contact for Reporting SAEs.

8.3.2. Evaluation of adverse events and serious adverse events

8.3.2.1. Active questioning to detect adverse events and serious adverse events

As a consistent method of soliciting AEs, the subject should be asked a non-leading question such as:

'*Have you felt different in any way since receiving the vaccine or since the previous visit?*'

When an AE/SAE occurs, it is the responsibility of the investigator to review all documentation (e.g. hospital progress notes, laboratory, and diagnostics reports) relative to the event. The investigator will then record all relevant information regarding an AE/SAE on the eCRF or SAE Report screens as applicable. It is not acceptable for the investigator to send photocopies of the subject's medical records to GSK Biologicals instead of the appropriate completed AE/SAE screens in the eCRF. However, there may be instances when copies of medical records for certain cases are requested by GSK Biologicals, MVI and Crucell. In this instance, all subject identifiers will be blinded on the copies of the medical records prior to submission to GSK Biologicals, MVI and Crucell.

The investigator will attempt to establish a diagnosis pertaining to the event based on signs, symptoms, and/or other clinical information. In such cases, the diagnosis should be documented as the AE/SAE and not the individual signs/symptoms.

8.3.2.2. Assessment of adverse events

8.3.2.2.1. Assessment of intensity

Intensity of the following AEs will be assessed as described:

Table 27 Intensity scales for solicited symptoms

Adverse Event	Intensity grade	Parameter
Pain at injection site	0	None
	1	Mild: Any pain neither interfering with nor preventing normal every day activities.
	2	Moderate: Painful when limb is moved and interferes with every day activities.
	3	Severe: Significant pain at rest. Prevents normal every day activities.
Redness at injection site		Record greatest surface diameter in mm
Swelling at injection site		Record greatest surface diameter in mm
Fever*		Record temperature in °C/°F
Headache	0	Normal
	1	Mild: Headache that is easily tolerated
	2	Moderate: Headache that interferes with normal activity
	3	Severe: Headache that prevents normal activity
Fatigue	0	Normal
	1	Mild: Fatigue that is easily tolerated
	2	Moderate: Fatigue that interferes with normal activity
	3	Severe: Fatigue that prevents normal activity
Gastrointestinal symptoms (nausea, vomiting, diarrhoea and/or abdominal pain)	0	Gastrointestinal symptoms normal
	1	Mild: Gastrointestinal symptoms that are easily tolerated
	2	Moderate: Gastrointestinal symptoms that interfere with normal activity
	3	Severe: Gastrointestinal symptoms that prevent normal activity
Chills	0	Normal (no chills)
	1	Feel cold without obvious cause
	2	Shivering (controllable, <15 sec)
	3	Rigors (uncontrollable shivering >15 sec)

*Fever is defined as: axillary/oral/tympanic temperature ≥38.0°C (100.4°F). The preferred route for recording temperature in this study will be oral.

The maximum intensity of local injection site redness/swelling will be scored at GSK Biologicals as follows:

0	:	0 mm
1	:	> 0 - ≤ 50 mm
2	:	> 50 - ≤ 100 mm
3	:	> 100 mm

The maximum intensity of fever (oral measurement) will be scored at GSK Biologicals as follows:

0	:	< 38.0°C or 100.4°F
1	:	≥38.0 - < 38.5°C or 100.4 - 101.2°F
2	:	≥38.5 - < 39.0°C or 101.2 - 102.1°F
3	:	≥ 39.0°C or 102.1°F

The investigator will assess the maximum intensity that occurred over the duration of the event for all other AEs, i.e. unsolicited symptoms, including SAEs reported during the study. The assessment will be based on the investigator's clinical judgement.

The intensity of each AE and SAE recorded in the eCRF or SAE Report screens, as applicable, should be assigned to one of the following categories:

1 (mild)	=	An AE which is easily tolerated by the subject, causing minimal discomfort and not interfering with everyday activities.
2 (moderate)	=	An AE which is sufficiently discomforting to interfere with normal everyday activities.
3 (severe)	=	An AE which prevents normal, everyday activities<br><br>(In adults/adolescents, such an AE would, for example, prevent attendance at work/school and would necessitate the administration of corrective therapy.)

An AE that is assessed as Grade 3 (severe) should not be confused with a SAE. Grade 3 is a category utilised for rating the intensity of an event; and both AEs and SAEs can be assessed as Grade 3. An event is defined as 'serious' when it meets one of the pre-defined outcomes as described in Section 8.1.2.

Acceptable/normal ranges and toxicity grading for laboratory safety parameters are provided in Table 28.

Table 28 Normal ranges and toxicity grading scales for blood testing

Adverse event	Intensity grade	Intensity*
Hemoglobin (males)	Normal range	12.5-17.0 gm/dL
	1	<12.5 but ≥11.0 gm/dL
	2	<11.0 but ≥10.0 gm/dL
	3	<10.0 gm/dL
Hemoglobin (females)	Normal range	11.5-15.0 gm/dL
	1	<11.5 but ≥10.5 gm/dL
	2	<10.5 but ≥9.5 gm/dL
	3	<9.5 gm/dL
Increase in Leukocytes (WBC)	Normal range	3,200 - 10,799 cells/mm^3
	1	10,800 - 15,000 cells/mm^3
	2	15,001 - 20,000 cells/mm^3
	3	>20,001 cells/mm^3
Decrease in Leukocytes (WBC)	Normal range	3,200 - 10,800 cells/mm^3
	1	2,500 - 3,199 cells/mm^3
	2	1,500 - 2,499 cells/mm^3
	3	< 1,500 cells/mm^3
Decrease in Platelets	Normal	140,000 - 400,000 cells/mm^3
	1	125,000 - 139,000 cells/mm^3
	2	100,000 - 124,000 cells/mm^3
	3	< 100,000 cells/mm^3
ALT	Normal range	Below ULN (60 U/L for males; 40 U/L for females
	1	1.1 - 2.5 x ULN
	2	2.6 – 5 x ULN
	3	> 5 x ULN
AST	Normal range	Below ULN (40 U/L for males; 35 U/L for females
		1.1 - 2.5 x ULN
		2.6 – 5 x ULN
		> 5 x ULN
Creatinine (Males)	Normal range	0.5 - 1.39 mg/dL
	1	1.4 - 1.79 mg/dL
	2	1.8 - 2.0 mg/dL
	3	> 2.0 mg/dL
Creatinine (Females)	Normal range	0.5 - 1.29 mg/dL
	1	1.3 – 1.69 mg/dL
	2	1.7 – 1.9 mg/dL
	3	>1.9 mg/dL

ULN: upper limit of normal range

*Grading scale adapted from Toxicity Grading scale for healthy adult and adolescent volunteers enrolled in preventive vaccine clinical trials September, 2007

8.3.2.2.2. Assessment of causality

The investigator is obligated to assess the relationship between investigational product and the occurrence of each AE/SAE. The investigator will use clinical judgement to determine the relationship. Alternative plausible causes, based on natural history of the underlying diseases, concomitant therapy, other risk factors and the temporal relationship of the event to the investigational product will be considered and investigated. The investigator will also consult the Investigator Brochure, in the determination of his/her assessment.

There may be situations when a SAE has occurred and the investigator has minimal information to include in the initial report to GSK Biologicals, MVI and Crucell. However, it is very important that the investigator always makes an assessment of causality for every event prior to submission of the SAE to GSK Biologicals, MVI and Crucell. The investigator may change his/her opinion of causality in light of follow-up information, amending the SAE information accordingly. The causality assessment is one of the criteria used when determining regulatory reporting requirements.

In case of concomitant administration of multiple vaccines, it may not be possible to determine the causal relationship of general AEs to the individual vaccines administered. The investigator should, therefore, assess whether the AE could be causally related to vaccination rather than to the individual vaccines.

All solicited local (injection site) reactions will be considered causally related to vaccination. Causality of all other AEs should be assessed by the investigator using the following question:

Is there a reasonable possibility that the AE may have been caused by the investigational product?

NO	:	The AE is not causally related to administration of the study vaccine(s). There are other, more likely causes and administration of the study vaccine(s) is not suspected to have contributed to the AE.
YES	:	There is a reasonable possibility that the vaccine(s) contributed to the AE.

Non-serious and serious AEs will be evaluated as two distinct events. If an event meets criteria to be determined 'serious' (see Section 8.1.2 for definition of serious adverse event), additional examinations/tests will be performed by the investigator in order to determine ALL possible contributing factors applicable to each SAE.

Possible contributing factors include:

- Medical history.
- Other medication.
- Protocol required procedure.
- Other procedure not required by the protocol.
- Lack of efficacy of the vaccines, if applicable.
- Erroneous administration.
- Other cause (specify).

8.3.2.3. Assessment of outcomes

Outcome of any non-serious AE occurring within 30 days post-vaccination or post-challenge (i.e. unsolicited AE) or any SAE reported during the entire study will be assessed as:

- Recovered/resolved.
- Not recovered/not resolved.
- Recovering/resolving.
- Recovered with sequelae/resolved with sequelae.
- Fatal (SAEs only).

8.4. Reporting of serious adverse events, pregnancies and other events

8.4.1. Prompt reporting of serious adverse events, pregnancies and other events to GSK Biologicals, MVI and Crucell

SAEs that occur the time period defined in Section 8.3 will be reported promptly to GSK Biologicals, MVI and Crucell as described in Table 29 once the investigator determines that the event meets the protocol definition of an SAE.

Pregnancies that occur the time period defined in Section 8.3 will be reported promptly to GSK Biologicals, MVI and Crucell as described in Table 29 once the investigator becomes aware of a pregnancy.

pIMDs that occur the time period defined in Section 8.3 will be reported promptly to GSK Biologicals, MVI and Crucell within the timeframes described in Table 29, once the investigator becomes aware of the pIMD.

Table 29 Time frames for submitting SAEs, pregnancy and other events reports to GSK Biologicals, MVI and Crucell

Type of Event	Initial Reports		Follow-up of Relevant Information on a Previous Report	
	Time Frame	Documents	Time Frame	Documents
All SAEs	24 hours*	SAE screen	24 hours*	SAE screen
Pregnancy	24 hours*	Pregnancy Report Form	24 hours*	Pregnancy Report Form
**pIMDs**	24 hours**	SAE screen	24 hours*	SAE screen

* Time frame allowed after receipt or awareness of the information.
**Timeframe allowed after the diagnosis is established and known to the investigator

8.4.2. Contact information for reporting serious adverse events and other events to GSK Biologicals

In case the electronic reporting system is temporarily unavailable, a back up system is in place. Please refer to Section 8.4.3 for a detailed description.

Study Contact for Reporting SAEs
Please see the Sponsor Information Sheet for contact details.
Back-up Study Contact for Reporting SAEs
GSK Biologicals Clinical Safety & Pharmacovigilance<br>Fax: +32 2 656 51 16 or +32 2 656 80 09<br>24/24 hour and 7/7 day availability

8.4.3. Completion and transmission of SAEs reports to GSK Biologicals, MVI and Crucell

Once an investigator becomes aware that a SAE has occurred in a study subject, the investigator will complete and submit the information in the SAE screens in eCRF within 24 hours. The SAE screens in eCRF will always be completed as thoroughly as possible with all available details of the event and will be submitted by the investigator. If the investigator does not have all information regarding an SAE, he/she will not wait to receive additional information before notifying GSK Biologicals of the event and completing the SAE screens in eCRF. The SAE screens in eCRF should be updated when additional relevant information is received WITHIN 24 HOURS.

Every SAE event will be reported to the funding agency, MVI and to Crucell within the same time frame.

The investigator will always provide an assessment of causality at the time of the initial report.

8.4.3.1. Back-up system in case the electronic SAE reporting system does not work

If the electronic SAE reporting system does not work, the investigator (or designate) must complete, then date and sign a SAE Report Form and fax it to the GSK Biologicals Clinical Safety and Pharmacovigilance department within 24 hours.

This back-up system should only be used if the electronic SAE reporting system is not working and NOT if the system is slow. As soon as the electronic SAE reporting system is working again, the investigator (or designate) must complete the SAE screens in the eCRF within 24 hours. The final valid information for regulatory reporting will be the information reported through the electronic SAE reporting system.

8.4.3.2. Updating of SAE information after freezing of the subject's eCRF

When additional information is received on a SAE after freezing of the subject's eCRF, new or updated information should be recorded on a SAE Report Form, with all changes signed and dated by the investigator. The updated form should be faxed to the GSK Biologicals Clinical Safety and Pharmacovigilance department or to the Study Contact for Reporting SAEs (refer to the Sponsor Information Sheet) WITHIN 24 HOURS of receipt of the follow-up information.

8.4.4. Completion and transmission of pregnancy reports to GSK Biologicals

Once the investigator becomes aware that a subject is pregnant, the investigator (or designate) must complete a Pregnancy Report Form and fax it to the Study Contact for Reporting SAEs (refer to the Sponsor Information Sheet) WITHIN 24 HOURS.

The Pregnancy Report Form will always be completed as thoroughly as possible with all available details and then dated and signed by the investigator (or designate). Even if the investigator does not have all information regarding the pregnancy, the form should still be completed and forwarded to GSK within 24 hours. Once additional relevant information is received, the form will be updated and forwarded to GSK WITHIN 24 HOURS.

In absence/dysfunction of facsimile equipment, the Study Contact for Reporting SAEs should be notified by telephone within 24 hours. As soon as the facsimile equipment is working again, the investigator (or designate) must fax the Pregnancy Report Form to the Study Contact for Reporting SAEs (refer to the Sponsor Information Sheet) within 24 hours.

8.4.5. Reporting of pIMDs to GSK Biologicals

Once onset of a new pIMD or exacerbation of a pre-existing pIMD is diagnosed (serious or non-serious) in a study subject, the investigator (or designate) must complete the information in the SAE screens of the eCRF WITHIN 24 HOURS after the he/she becomes aware of the diagnosis. A field on the SAE screen allows to specify that the event is a pIMD and whether it is serious or non serious. The SAE screens will always be completed as thoroughly as possible with all available details of the event, in accordance with the pIMD standard questionnaire provided. Even if the investigator does not have all information regarding a pIMD, the SAE screens should still be completed within 24 hours. Once additional relevant information is received, the SAE screens in the eCRF should be updated WITHIN 24 HOURS.

The investigator will always provide an assessment of causality at the time of the initial report.

Refer to Sections 8.4.3.1 and 8.4.3.2 for back-up system and updating of SAE information after freezing of the subject's eCRF.

8.4.6. Regulatory reporting requirements for serious adverse events

The investigator will promptly report all SAEs to GSK Biologicals in accordance with the procedures detailed in Section 8.4.1. GSK Biologicals has a legal responsibility to promptly notify, as appropriate, both the local regulatory authority and other regulatory agencies about the safety of a product under clinical investigation. Prompt notification of SAEs by the investigator to the Study Contact for Reporting SAEs is essential so that legal obligations and ethical responsibilities towards the safety of other subjects are met.

Investigator safety reports are prepared according to the current GSK policy and are forwarded to investigators as necessary. An investigator safety report is prepared for a SAE(s) that is both attributable to the investigational product and unexpected. The purpose of the report is to fulfill specific regulatory and Good Clinical Practice (GCP) requirements, regarding the product under investigation.

8.5. Follow-up of adverse events, serious adverse events, and pregnancies

8.5.1. Follow-up of adverse events and serious adverse events

After the initial AE/SAE report, the investigator is required to proactively follow each subject and provide further information on the subject's condition to GSK Biologicals.

All SAEs and pIMDs documented at a previous visit and designated as not recovered/not resolved or recovering/resolving will be reviewed at subsequent visits until the end of the study.

All AEs documented at a previous visit and designated as not recovered/not resolved or recovering/resolving will be reviewed at subsequent visits until 30 days after the last vaccination.

Investigators will follow-up subjects:

- With SAEs or subjects withdrawn from the study as a result of an AE, until the event has resolved, subsided, stabilised, disappeared, or until the event is otherwise explained, or the subject is lost to follow-up.
- Or, in the case of other non-serious AEs, until they complete the study or they are lost to follow-up.

Clinically significant laboratory abnormalities will be followed up until they have returned to normal, or a satisfactory explanation has been provided. Additional information (including but not limited to laboratory results) relative to the subsequent course of such abnormalities noted for any subject must be made available to the Site Monitor.

GSK Biologicals may request that the investigator performs or arranges for the conduct of additional clinical examinations/tests and/or evaluations to elucidate as fully as possible the nature and/or causality of the AE or SAE. The investigator is obliged to assist. If a subject dies during participation in the study or during a recognised follow-up period, GSK Biologicals will be provided with a copy of any available post-mortem findings, including histopathology.

8.5.2. Follow-up of pregnancies

Pregnant subjects will be followed to determine the outcome of the pregnancy. At the end of the pregnancy, whether full-term or premature, information on the status of the mother and child will be forwarded to GSK Biologicals. Generally, the follow-up period doesn't need to be longer than six to eight weeks after the estimated delivery date.

8.6. Treatment of adverse events

Treatment of any AE is at the sole discretion of the investigator and according to current good medical practice. Any medication administered for the treatment of an AE should be recorded in the subject's eCRF. Refer to Section 6.7.

8.7. Unblinding

GSK Biologicals' policy (which incorporates ICH E2A guidance, EU Clinical Trial Directive and US Federal Regulations) is to unblind the report of any SAE which is unexpected and attributable/suspected to be attributable to the investigational product, prior to regulatory reporting. The GSK Biologicals' Central Safety Physician is responsible for unblinding the treatment assignment in accordance with the specified timeframes for expedited reporting of SAEs (refer to Section 8.4.1).

8.8. Emergency unblinding

Unblinding of a subject's individual randomisation code should be requested only in case of a medical emergency, or in the event of a serious medical condition, when knowledge of the investigational study vaccines is essential for the clinical management or welfare of the subject.

The investigator, or any other physician managing the subject, should contact GSK Biologicals' Central Safety Physician by telephone to discuss the need for emergency unblinding. Alternatively, the investigator may communicate with the Study Contact for Emergency Code Break, who will then discuss the need for emergency unblinding with the GSK Biologicals' Central Safety Physician. After the initial phone contact, the investigator (or designate) must complete, then date and sign an Emergency Unblinding Request Form and send it to GSK Biologicals. The preferred method to forward the form to GSK Biologicals will be communicated by the Central Safety Physician during the phone contact.

The GSK Biologicals' Central Safety Physician has access to the subject's individual randomisation code and can authorise emergency unblinding upon request.

Contact information for Emergency Unblinding Request<br>24/24 hour and 7/7 day availability
**GSK Biologicals' Central Safety Physician:** +1 877 441 0017 (GSK Biologicals Central Safety Physician on-call)<br>**Back-up phone contact:** + 1 877 441 0015<br>**Study Contact for Emergency Code Break:**<br>See Sponsor Information Sheet

8.9. Subject card

Study subjects must be provided with the address and telephone number of the main contact for information about the clinical study.

The investigator (or designate) must therefore provide a "subject card" to each subject. In an emergency situation this card serves to inform the responsible attending physician that the subject is in a clinical study and that relevant information may be obtained by contacting the investigator.

Subjects must be instructed to keep subject cards in their possession at all times.

9. SUBJECT COMPLETION AND WITHDRAWAL

9.1. Subject completion

A subject who returns for the concluding visit/is available for the concluding contact foreseen in the protocol is considered to have completed the study.

9.2. Subject withdrawal

A subject may end his or her participation in the study at any time, but unless they choose to completely withdraw consent for study participation they will be followed up for safety until study conclusion.

Withdrawals will not be replaced.

9.2.1. Subject withdrawal from the study

From an analysis perspective, a 'withdrawal' from the study refers to any subject who did not come back for the concluding visit/was not available for the concluding contact foreseen in the protocol.

All data collected until the date of withdrawal/last contact of the subject will be used for the analysis.

A subject is considered a 'withdrawal' from the study when no study procedure has occurred, no follow-up has been performed and no further information has been collected for this subject from the date of withdrawal/last contact.

Investigators will make an attempt to contact those subjects who do not return for scheduled visits or follow-up.

Information relative to the withdrawal will be documented in the eCRF. The investigator will document whether the decision to withdraw a subject from the study was made by the subject himself/herself or by the investigator, as well as which of the following possible reasons was responsible for withdrawal:

- Serious adverse event.
- Non-serious adverse event.
- Protocol violation (specify).
- Consent withdrawal, not due to an adverse event.
- Moved from the study area.
- Lost to follow-up.
- Other (specify).

9.2.2. Subject withdrawal from investigational vaccine

A ‘withdrawal’ from the investigational vaccine refers to any subject who does not receive the complete treatment, i.e. when no further planned dose is administered from the date of withdrawal. A subject withdrawn from the investigational vaccine may not necessarily be withdrawn from the study as further study procedures or follow-up may be performed (safety or immunogenicity) if planned in the study protocol.

Information relative to premature discontinuation of the investigational vaccine will be documented on the Vaccine Administration screen of the eCRF. The investigator will document whether the decision to discontinue further vaccination/treatment was made by the subject himself/herself, or by the investigator, as well as which of the following possible reasons was responsible for withdrawal:

- Serious adverse event.
- Non-serious adverse event.
- Other (specify).

A subject may also be withdrawn from the investigational product as per described stopping criteria in Section 5.4.4.1.

10. DATA EVALUATION: CRITERIA FOR EVALUATION OF OBJECTIVES

10.1. Primary endpoint

Primary efficacy endpoint

- Occurrence of *P. falciparum* parasitemia, defined by a positive blood slide, following sporozoite challenge.

Primary safety endpoints

- Occurrence of each solicited adverse event within 7-day follow-up period (day of vaccination and 6 subsequent days) after each vaccination.
- Occurrence of unsolicited adverse events within 30 days (day of vaccination and 29 subsequent days) after each vaccination, according to the Medical Dictionary for Regulatory Activities (MedDRA) classification.
- Occurrence of unsolicited adverse events within 30 days (day of challenge and 29 subsequent days) after challenge, according to the Medical Dictionary for Regulatory Activities (MedDRA) classification.
- Occurrence of serious adverse events (SAEs) within 30 days (day of vaccination and 29 subsequent days) after each vaccination, according to the Medical Dictionary for Regulatory Activities (MedDRA) classification.
- Occurrence of SAEs during the whole study period (from dose 1 up to study conclusion), according to the Medical Dictionary for Regulatory Activities (MedDRA) classification.

10.2. Secondary endpoints

Secondary efficacy endpoint

- Time to *P. falciparum* parasitemia, defined by a positive blood slide, following sporozoite challenge.

Secondary immunogenicity endpoints

- Anti-CS (RT) and anti-HBs antibody titres at specified time points.
- Anti-Ad35 neutralizing antibody titers at specified time points.
- Frequency of CS (total CS or RT)-specific, HBs-specific T cells at specified time points.

10.3. Exploratory endpoints

Exploratory efficacy endpoints

- Occurrence of *P. falciparum* parasitemia, defined by a positive PCR, following sporozoite challenge.
- Time to *P. falciparum* parasitemia, defined by a positive PCR, following sporozoite challenge.

Exploratory immunogenicity endpoints

- Frequency of Ad35-specific T cells at specified time points.
- Antibody titres against full-length CS and CS specific peptides other than repeat.
- Isotype, affinity and functionality of CS-specific antibodies.
- Multivariant analysis (systems biology approach) using high-throughput technologies such as gene expression profiling, multiplex analysis of cytokines and chemokines (Luminex), and multiparameter flow cytometry for plasmablast analysis and B cell ELISPOT.

10.4. Estimated sample size

For the final analysis, the target enrollment for this study will be 168 volunteers to ensure 69 subjects per study groups will undergo sporozoite challenge, given an approximate 20% estimated drop-out rate, based on past experience.

For the primary endpoint, the study will have 82% power to demonstrate an increase of 50% efficacy (2-sided Fisher exact test, alpha=5%) of the ARR group over the RRR group, assuming a 50% attack rate in the RRR group. The increase in VE of the ARR group over the RRR alone group is defined as 100*(1-AR Ad35.CS / AR RTS,S) where AR is the attack rate in the 2 groups.

The trial will have 80% power (2-sided Fisher exact test, alpha=5%) to detect the following fold increase in AE rates of the ARR group over the RRR group (right column), depending on the rate of AEs in the RRR group (left column) (see Table 30).

Table 30 Percentages with 80 percent power to detect event rate fold increase in the ARR group

SAEs rates in RRR group	80% power to detect event rate fold increase in ARR group
1%	12
5%	4

ARR group = first dose with Ad35.CS.01, second and third doses with RTS,S/$AS01_B$
RRR group = three doses of RTS,S/$AS01_B$

10.5. Study cohorts to be evaluated

10.5.1. Total vaccinated cohort

The Total vaccinated cohort for analysis of safety will include all subjects with at least one vaccine administration documented. The Total vaccinated cohort analysis will be performed per treatment actually administered.

10.5.2. According-to-protocol (ATP) cohort for analysis of efficacy

The ATP cohort for analysis of efficacy will include all subjects included in the Total vaccinated cohort who received all vaccinations according to protocol procedures within the protocol specified intervals, underwent challenge and for whom data concerning efficacy endpoint measures are available.

10.5.3. According-to-protocol (ATP) cohort for analysis of immunogenicity

The ATP cohort for analysis of immunogenicity will include all evaluable subjects from the Total vaccinated cohort:

- Who meet all eligibility criteria.
- Who comply with the procedures and intervals defined in the protocol.
- Who are within the maximum interval allowed as defined in the protocol. Refer to Table 11 for the interval between study visits that will be considered for inclusion in the ATP cohort for analysis of immunogenicity.
- Who did not receive a product leading to exclusion from an ATP analysis as listed Section 6.7.1.
- Who did not present with a medical condition leading to exclusion from an ATP analysis as for Section 6.8.
- For whom data concerning immunogenicity endpoint measures are available. This will include subjects for whom assay results are available for antibodies against at least one study vaccine antigen component after vaccination.

10.6. Derived and transformed data

Immunogenicity

- The cut-off value is defined by the laboratory before the analysis and is described in Section 5.7.3.
- A seronegative subject is a subject whose titer is below the cut-off value.
  - Anti-CS repeat antibody titers < 0.5 EU/mL.
  - Anti-CS full-length and peptides antibody titers < 200
  - Anti-Ad35 antibody titers < 16 IC90.

- A seropositive subject is a subject whose titer is greater than or equal to the cut-off value for the following antigens:
  - Anti-CS repeat antibody titers ≥ 0.5 EU/mL.
  - Anti-CS full-length and peptides antibody titers ≥ 200
  - Anti-Ad35 antibody titers > 16 IC90.
- A hepatitis B virus seroprotected subject is defined as a subject whose antibody titer is greater than or equal to the cut-off value for the following antigen:
  - Anti-HBs antibody titers ≥ 10 mIU/mL.
- The Geometric Mean Titers (GMTs) calculations are performed by taking the anti-log of the mean of the log titer transformations. Antibody titers below the cut-off of the assay will be given an arbitrary value of half the cut-off for the purpose of GMT calculation.
- Handling of missing data:

  For a given subject and a given immunogenicity measurement, missing or non-evaluable measurements will not be replaced. Therefore, an analysis will exclude subjects with missing or non-evaluable measurements.

Reactogenicity and Safety

- Handling of missing data:
  - Subjects who missed reporting symptoms (solicited/unsolicited or concomitant medications) will be treated as subjects without symptoms (solicited/unsolicited or concomitant medications, respectively). In case of significant non-compliance of study procedures for reporting symptoms, the analysis plan will be reassessed to ensure more accurate reporting of study data by further analysis.
  - For the analysis of solicited symptoms, missing or non-evaluable measurements will not be replaced. Therefore the analysis of the solicited symptoms based on the Total vaccinated cohort will include only subjects/doses with documented safety data (i.e. symptom screen/sheet completed).

10.7. Conduct of analyses

Any deviation(s) or change(s) from the original statistical plan outlined in this protocol will be described and justified in the final study report.

10.7.1. Sequence of analyses

The analyses will be performed stepwise:

- An interim analysis for futility will be performed on safety and efficacy data collected up to 28 days post-challenge (Study Day 105) for 46 subjects from Cohort A. Refer to Sections 5.4.4.1 and 5.4.4.2 for more details on the futility analysis.
- A primary analysis will be performed on all efficacy, immunogenicity and safety data collected up to 28 days post-challenge (Study Day 105) on all cohorts.

- A final analysis will be conducted at 6 months post-dose 3, including extended safety and immunogenicity follow-up. The final study report will be generated after final analysis.

10.7.2. Statistical considerations for interim analyses

An interim analysis for futility is planned on 46 subjects from cohort A. Analysis will be performed by a statistician external to the GSK-MVI-Crucell clinical teams and summary results will be communicated without unblinding individual trial subjects. In the event the trial is stopped for futility, the databases will be cleaned and locked and a clinical study report will be written. In the event the trial continues no clinical study report will be generated. However, in any case the database and analyses will be appropriately stored. As this analysis only looks at stopping for futility, no corrections of the overall alpha level are required.

10.8. Statistical methods

Analyses will be performed by cohort (A, B, C) and pooled across cohort where applicable and will be further specified in the statistical analysis plan.

10.8.1. Analysis of demographics/baseline characteristics

- Demographic characteristics (age in years, gender and geographic ancestry) of each study cohort will be tabulated per group.
- The distribution of subjects enrolled among the study centers will be tabulated.

10.8.2. Analysis of efficacy

Efficacy will be assessed on the ATP cohort for efficacy by comparison of malaria incidence and time to onset of parasitemia after sporozoite challenge. Vaccine efficacy is defined as 100*(1-Relative Risk). Fisher's Exact test will be used for the comparison of malaria incidence between the two vaccinated groups. Kaplan-Meier analysis will be performed on time to onset of parasitemia, with testing between the two treatment groups using the log-rank statistic. Relative risk of infection and 95% confidence intervals (95% CI) will be calculated. All statistical tests will be two-tailed at 5% significance level.

10.8.3. Analysis of immunogenicity

Immunogenicity analysis will be performed on the ATP cohort for analysis of immunogenicity.

Descriptive statistics and between group p-values will be calculated for all immunological read-outs.

Anti-CS, anti-Ad35 and anti-HBs antibody responses

For each study group, at each time point that a blood sample result is available:

- Seropositivity rates for CS, Ad35 (with exact 95% CI) will be calculated by group.

- Seroprotection rates for HBs (with exact 95% CI) will be calculated by group.
- GMTs with 95% CI will be tabulated for antibodies for each antigen by group.
- The distribution of antibody titers for each antigen will be displayed using tables and/or reverse cumulative distribution curves.

Cell mediated immune response (CMI) analysis

Descriptive analysis (mean, standard deviation, median, minimum and maximum) will be performed for the CMI endpoints. Graphical presentations of immune responses will be generated (Box-plots).

For intra-cellular staining (ICS) assays, the data will be presented as the frequency of CD4+ or CD8+ T cells per million PBMC expressing a given cytokine.

For ELISPOT assay, the data will be presented as the number of spots per million PBMC.

Immune response and vaccine efficacy

The relationship between immune responses and vaccine efficacy will be explored and further detailed in the statistical analysis plan.

10.8.4. Analysis of safety

Safety analysis will be performed on the Total vaccinated cohort.

The percentage of subjects with at least one local AE (solicited and unsolicited), with at least one general AE (solicited and unsolicited) and with any AE during the solicited follow-up period will be tabulated with exact 95% CI after each vaccine dose and overall. The percentage of doses followed by at least one local AE (solicited and unsolicited), by at least one general AE (solicited and unsolicited) and by any AE will be tabulated over the entire vaccination course, with exact 95% CI.

The percentage of subjects reporting each individual solicited local and general AE during the 7-day (Day 0-Day 6) solicited follow-up period will be tabulated with exact 95% CI. The percentage of doses followed by each individual solicited local and general AE will be tabulated over the entire vaccination course, with exact 95% CI.

The same tabulation will be performed for grade 3 AEs and for AEs with relationship to vaccination. The verbatim reports of unsolicited symptoms will be reviewed by a physician and the signs and symptoms will be coded according to the MedDRA Dictionary for Adverse Reaction Terminology. The percentage of subjects with at least one report of unsolicited AE classified by the Medical Dictionary for Regulatory Activities (MedDRA) and reported up to 30 days (Day 0-Day 29) after vaccination will be tabulated with exact 95% CI. The same tabulation will be performed for grade 3 unsolicited AEs and for unsolicited AEs with a relationship to vaccination.

The number and percentage of subjects who took concomitant antipyretic/medication at least once during the 7-day (Day 0-Day 6) solicited follow-up period will be tabulated for each group, after each vaccine dose and overall, with exact 95% CI. The number and

percentage of doses for which the subjects took concomitant antipyretic/medication at least once during the 7-day (Day 0-Day 6) solicited follow-up period will be tabulated for each group, over the entire vaccination course, with exact 95% CI.

SAEs, AEs of specific interest and withdrawal due to AE(s)/SAE(s) will be described in detail.

Hematological (hemoglobin, WBC and platelets) and biochemical (creatinine, ALT and AST) laboratory values will be presented according to toxicity grading scales and tabulated by group.

Urine laboratory values will be tabulated by group.

11. ADMINISTRATIVE MATTERS

To comply with ICH GCP administrative obligations relating to data collection, monitoring, archiving data, audits, confidentiality and publications must be fulfilled.

11.1. Remote Data Entry instructions

Remote Data Entry (RDE), a validated computer application, will be used as the method for data collection.

In all cases, subject initials will not be collected nor transmitted to GSK. Subject data necessary for analysis and reporting will be entered/transmitted into a validated database or data system. Clinical data management will be performed in accordance with applicable GSK standards and data cleaning procedures.

While completed eCRFs are reviewed by a GSK Biologicals' Site Monitor at the study site, omissions or inconsistencies detected by subsequent eCRF review may necessitate clarification or correction of omissions or inconsistencies with documentation and approval by the investigator or appropriately qualified designee. In all cases, the investigator remains accountable for the study data.

The investigator and the partners will be provided with a CD-ROM of the final version of the data generated at the investigational site once the database is archived and the study report is complete and approved by all parties.

11.2. Monitoring by GSK Biologicals

Monitoring visits by a GSK Site Monitor are for the purpose of confirming that GSK Biologicals' sponsored studies are being conducted in accordance with the ethical principles that have their origins in the Declaration of Helsinki and that are consistent with Good Clinical practice (GCP) and the applicable regulatory requirement(s) (verifying continuing compliance with the protocol, amendment(s), reviewing the investigational product accountability records, verifying that the site staff and facilities continue to be adequate to conduct the study).

The investigator must ensure provision of reasonable time, space and qualified personnel for monitoring visits.

Direct access to all study-site related and source data is mandatory for the purpose of monitoring review. The monitor will perform a RDE review and a Source Document Verification (SDV). By SDV we understand verifying RDE entries by comparing them with the source data that will be made available by the investigator for this purpose.

The Source Documentation Agreement Form describes the source data for the different data in the RDE. This document should be completed and signed by the site monitor and investigator and should be filed in the monitor's and investigator's study file. Any data item for which the RDE will serve as the source must be identified, agreed and documented in the source documentation agreement form.

For RDE, the monitor will mark completed and approved screens at each visit.

In accordance with applicable regulations, GCP, and GSK procedures, GSK monitors will contact the site prior to the start of the study to review with the site staff the protocol, study requirements, and their responsibilities to satisfy regulatory, ethical, and GSK requirements. When reviewing data collection procedures, the discussion will also include identification, agreement and documentation of data items for which the eCRF entries will serve as the source document.

GSK will monitor the study to verify that, amongst others, the:

- Data are authentic, accurate, and complete.
- Safety and rights of subjects are being protected.
- Study is conducted in accordance with the currently approved protocol and any amendments, any other study agreements, GCP and all applicable regulatory requirements.

The investigator and the head of the medical institution (where applicable) agrees to allow the monitor direct access to all relevant documents.

Upon completion or premature discontinuation of the study, the monitor will conduct site closure activities with the investigator or site staff, as appropriate, in accordance with applicable regulations, GCP, and GSK procedures.

11.3. Archiving of data at study sites

Following closure of the study, the investigator must maintain all site study records in a safe and secure location. The records must be maintained to allow easy and timely retrieval, when needed (e.g. audit or inspection), and, whenever feasible, to allow any subsequent review of data in conjunction with assessment of the facility, supporting systems, and staff. Where permitted by applicable laws/regulations or institutional policy, some or all of these records can be maintained in a validated format other than hard copy (e.g. microfiche, scanned, electronic for studies with an eCRF); however, caution needs to be exercised before such action is taken. The investigator must assure that all

reproductions are legible and are a true and accurate copy of the original and meet accessibility and retrieval standards, including re-generating a hard copy, if required. Furthermore, the investigator must ensure there is an acceptable back-up of these reproductions and that an acceptable quality control process exists for making these reproductions.

GSK will inform the investigator/institution of the time period for retaining these records to comply with all applicable regulatory requirements. However, the investigator/institution should seek the written approval of the sponsor before proceeding with the disposal of these records. The minimum retention time will meet the strictest standard applicable to that site for the study, as dictated by ICH GCP, any institutional requirements or applicable laws or regulations, or GSK standards/procedures; otherwise, the minimum retention period will default to 15 years.

The investigator/institution must notify GSK of any changes in the archival arrangements, including, but not limited to, the following: archival at an off-site facility, transfer of ownership of the records in the event the investigator leaves the site.

11.4. Audits

To ensure compliance with GCP and all applicable regulatory requirements, GSK may conduct a quality assurance audit. Regulatory agencies may also conduct a regulatory inspection of this study. Such audits/inspections can occur at any time during or after completion of the study. If an audit or inspection occurs, the investigator and institution agree to allow the auditor/inspector direct access to all relevant documents and to allocate his/her time and the time of his/her staff to the auditor/inspector to discuss findings and any relevant issues.

11.5. Posting of information on Clinicaltrials.gov

Study information from this protocol will be posted on clinicaltrials.gov before enrolment of subjects begins.

11.6. Ownership, confidentiality and publication

11.6.1. Ownership

All information provided by GSK is and remains the sole property of GSK. The ownership of all data and information generated by the site as part of the study (other than a subject's medical records) is governed by the Clinical Trial Agreement (CTA) between the site, GSK, MVI and Crucell.

Ownership of all rights, title, and interests in any inventions, know-how or other intellectual or industrial property rights which are conceived or reduced to practice by site staff during the course of or as a result of the study is governed by the CTA.

11.6.2. Confidentiality

Documented evidence that a potential investigator is aware and agrees to the confidential nature of the information related to the study must be obtained by means of a confidentiality agreement.

All information provided by GSK and all data and information generated by the site as part of the study (other than a subject's medical records) will be kept confidential by the investigator and other site staff. This information and data will not be used by the investigator or other site personnel for any purpose other than conducting the study. These restrictions do not apply to: (i) information which becomes publicly available through no fault of the investigator or site staff; (ii) information which it is necessary to disclose in confidence to an IEC or IRB solely for the evaluation of the study; (iii) information which it is necessary to disclose in order to provide appropriate medical care to a study subject; or (iv) study results which may be published as described in the next paragraph.

If the relevant CTA is inconsistent with this statement, that CTA's confidentiality provisions shall apply rather than the above statement.

11.6.3. Publication

Prior to submitting for publication, presentation, use for instructional purposes, or otherwise disclosing the study results generated by the site (collectively, a 'Publication'), the investigator shall provide GSK, MVI and Crucell with a copy of the proposed Publication and allow GSK, MVI and Crucell a period to review the proposed Publication (at least 60 working days prior to the Publication's submission). Proposed Publications shall not include either GSK's, MVI's or Crucell's confidential information.

At GSK's, MVI's or Crucell's request, the submission or other disclosure of a proposed Publication will be delayed for up to 90 days to allow those parties to seek patent or similar protection of any inventions, know-how or other intellectual or industrial property rights disclosed in the proposed Publication.

The publication provisions in the relevant CTA will supersede this statement.

11.6.4. Provision of study results to investigators, posting to the clinical trials registers and publication

Where required by applicable regulatory requirements, an investigator signatory will be identified for the approval of the clinical study report. The investigator will be provided reasonable access to statistical tables, figures, and relevant reports and will have the opportunity to review the complete study results at a GSK site or other mutually-agreeable location.

GSK will also provide the investigator with the full summary of the study results. The investigator is encouraged to share the summary results with the study subjects, as appropriate.

The results summary will be posted to the GSK Clinical Study Register at the time of the first regulatory approval or within 12 months of any decision to terminate development. In addition, a manuscript will be submitted to a peer-reviewed journal for publication within 12 months of the first approval or within 12 months of any decision to terminate development. When manuscript publication in a peer-reviewed journal is not feasible, further study information will be posted to the GSK Clinical Study Register to supplement the results summary.

12. COUNTRY SPECIFIC REQUIREMENTS

Not applicable.

13. REFERENCES

Ballou WR, Sherwood JA, Neva FA, et al. Safety and efficacy of a recombinant DNA *Plasmodium falciparum* sporozoite vaccine. *Lancet* 1987;8545:1277-1281.

Catanzaro AT, Koup RA, Roederer M et al. Phase 1 safety and immunogenicity evaluation of a multiclade HIV-1 candidate vaccine delivered by a replicationdefective recombinant adenovirus vector. *J Infect Dis* 2006;194:1638-1649.

Chulay JD, Schneider I, Cosgriff TM et al.Malaria transmitted to humans by mosquitoes infected from cultured Plasmodium falciparum. *Am J Trop Med Hyg* 1986;35: 66-68.

Epstein JE, Rao S, Williams F et al. Safety and clinical outcome of experimental challenge of human volunteers with Plasmodium falciparum-infected mosquitoes: an update. *J Infect Dis* 2007; 196(1):145-154

Gordon DM, McGovern TW, Krzych U, et al. Safety, immunogenicity, and efficacy of a recombinantly produced Plasmodium falciparum circumsporozoite protein / HBsAg subunit vaccine. *J Infect Dis* 1995;171:1576-85.

Graham BS. Update on VRC Clinical Trials of Multi-Clade DNA and rAd5 Vaccines. Proceedings of the Keystone Conference on HIV. *Vaccines*; 2005 Apr 9-15; Banff, Alberta, USA [Abstract 041].

Hadler SC, Francis DP, Maynard JE, et al. Long-term immunogenicity and efficacy of Hepatitis B vaccine in homosexual men. *N Eng J Med* 1986; 315(4): 209-214.

Harro C, Sun X, Stek JE et al. Safety and immunogenicity of the Merck adenovirus serotype 5 (MRKAd5) and MRKAd6 human immunodeficiency virus type 1 trigene vaccines alone and in combination in healthy adults. *Clin Vaccine Immunol* 2009;16(9):1285-1292.

Hill AV, Reyes-Sandoval A, O'Hara G et al. Prime-boost vectored malaria vaccines: Progress and prospects. *Hum Vaccin* 2010;6(1):78-83.

Hu SL, Abrams K, Barber GN et al. Protection of macaques against SIV infection by subunit vaccines of SIV envelope glycoprotein gp160. *Science* 1992;255(5043):456-459.

Kester KE, Cummings JF, Ofori-Anyinam O et al. Randomized, double-blind, Phase 2a trial of falciparum malaria vaccines RTS,S/AS01$_B$ and RTS,S/AS02$_A$ in malaria-naïve

adults; safety, efficacy and immunologic associates of protection. *J Infect Dis* 2009;200(3):337-346.

Kostense S, Koudstaal W, Sprangers M et al. Adenovirus types 5 and 35 seroprevalence in AIDS risk groups supports type 35 as a vaccine vector. *Aids* 2004;18:1213-1216.

Lu S. Heterologous prime-boost vaccination. *Curr Opin Immunol* 2009;21(3):346-351.

Lubeck MD, Natuk R, Myagkikh M et al. Long-term protection of chimpanzees against high-dose HIV-1 challenge induced by immunization. *Nat Med* 1997;3(6):651-658.

Nieman AE, de Mast Q, Roestenberg M et al. Cardiac complication after experimental human malaria infection: a case report. *Malar J* 2009; 3(8):277.

Querec TD, Akondy RS, Lee EK et al. Systems biology approach predicts immunogenicity of the yellow fever vaccine in humans. *Nat Immunol.* 2009; 10(1):116-25.

Radosević K, Rodriguez A, Lemckert A et al. Heterologous prime-boost vaccinations for poverty-related diseases: advantages and future prospects. *Expert Rev Vaccines* 2009;8(5):577-52.

Rodríguez A, Goudsmit J, Companjen A et al. Impact of recombinant adenovirus serotype 35 priming versus boosting of a *Plasmodium falciparum* protein: characterization of T- and B-cell responses to liver-stage antigen 1. *Infect Immun* 2008;76(4):1709-1718.

Sprangers MC, Lakhai W, Koudstaal W et al. Quantifying adenovirus-neutralizing antibodies by luciferase transgene detection: addressing preexisting immunity to vaccine and gene therapy vectors. *J Clin Micr* 2003; (41) 11:5046-5052.

Shott JP, McGrath SM, Pau MG et al. Adenovirus 5 and 35 vectors expressing *Plasmodium falciparum* circumsporozoite surface protein elicit potent antigen-specific cellular IFN-gamma and antibody responses in mice. *Vaccine* 2008;26(23):2818-2823.

Stewart VA, McGrath SM, Dubois PM et al. Priming with an adenovirus 35-circumsporozoite protein (CS) vaccine followed by RTS,S/$AS01_B$ boosting significantly improves immunogenicity to *Plasmodium falciparum* CS compared to that with either malaria vaccine alone. *Infect Immun* 2007;75(5):2283-2290.

Verhage DF, Telgt DS, Bousema JT et al. Clinical outcome of experimental human malaria induced by Plasmodium falciparum-infected mosquitoes. *Neth J Med* 2005; 63(2):52-58.

Vogels R, Zuijdgeest D, van Rijnsoever R et al. Replication-deficient human adenovirus type 35 vectors for gene transfer and vaccination: efficient human cell infection and bypass of preexisting adenovirus immunity. *J Virol* 2003;77:8263-8271.

Wirtz RA, Ballou WR, Schneider I, et al. *Plasmodium falciparum*: Immunogenicity of circumsporozoite protein constructs produced in *Escherichia coli*. *Exp Parasitol* 1987;63:166-72.

Appendix A NHANES I cardiovascular risk criteria

Using the Graziano, et. al. article, volunteers will be screened for cardiac risk factors and be given a screening electrocardiogram. The information will be recorded on a source document with the following noted:

Study ID # ______________

Risk factors

Weight___ kg	Blood pressure ________
Height_____	Smoker Y / N
Calculated BMI (kg/m^2)_____	Diabetes Y / N

Using Table A (males) or Table B (females), 5-year cardiovascular risk:

□ Low

□ Moderate

□ High

Electrocardiogram (EKG)

12-lead EKG taken? Y / N

If not,
reason____

Electrocardiogram interpreted by

____

Electrocardiogram interpretation
____

Only volunteers with NHANES I low risk criteria as well as non-significant EKG, as determined by expert consultant cardiologist, are accepted in the study.

Table A (for Males)

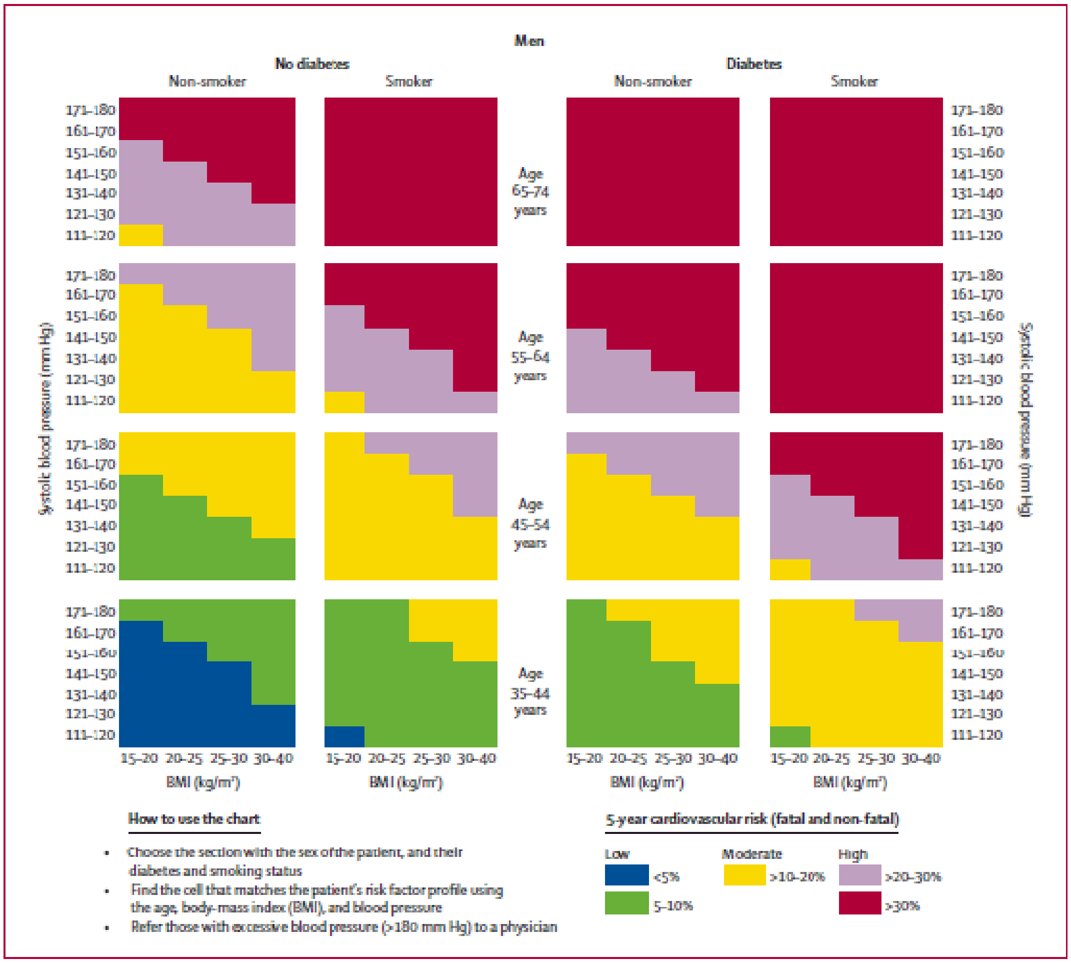

Table B (for Females)

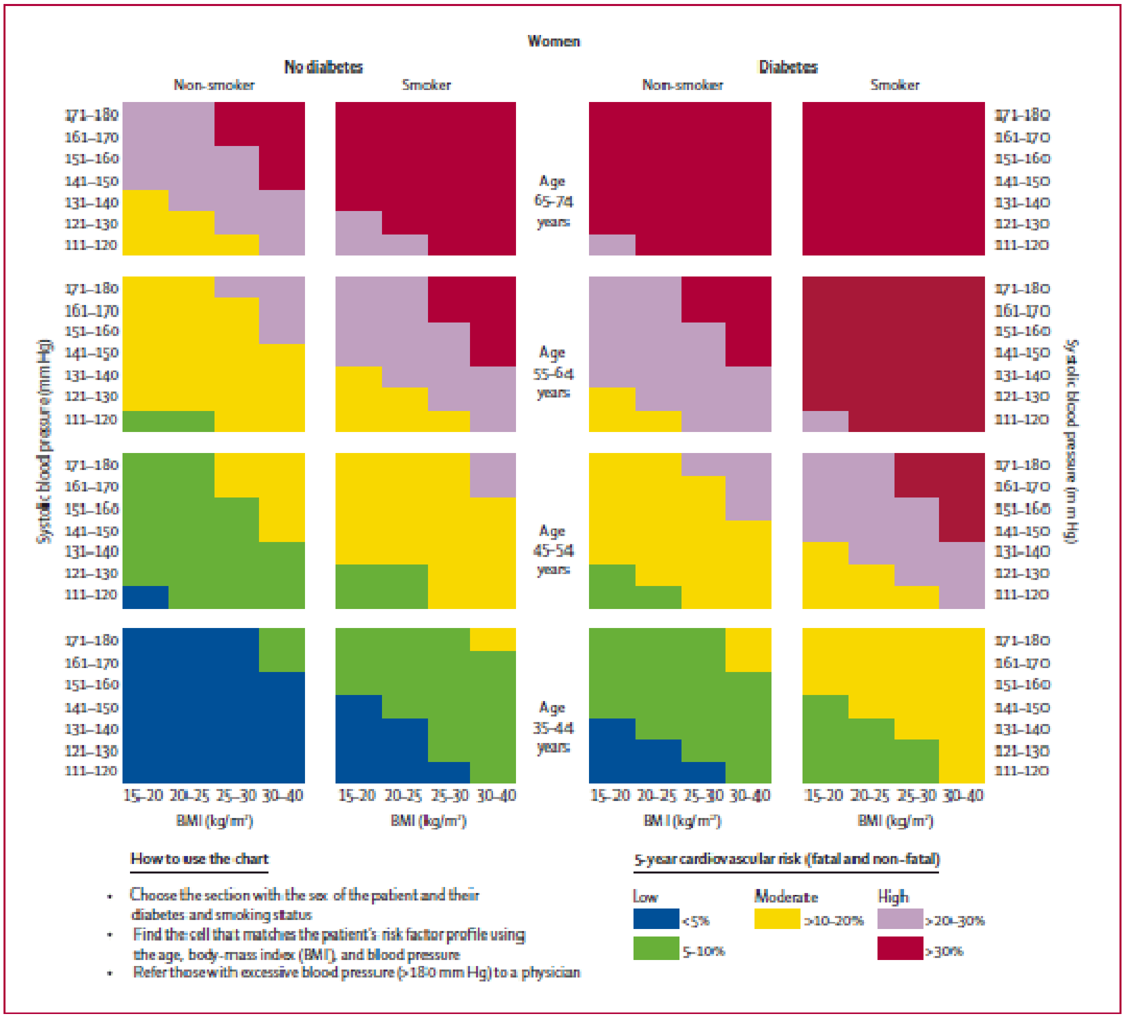

Appendix B Neurologic Examination Form

Cranial nerves

Eye movements Normal/Abnormal

Tongue movements Normal/Abnormal

Facial expression Normal/Abnormal

Muscle strength grading (Normal: muscle can move in full range of motion, against full resistance. Abnormal: 0 Complete paralysis, no visible movement ; 1 Trace strength, some visible muscle contraction, with no movement of joint ; 2 poor strength, muscle can move the joint but not against gravity ; 3 Fair strength, muscle can move against gravity, but not against any examiner resistance ; 4 Good strength, muscle can move against moderate resistance, but not examiner full resistance).

Deltoid (non-dominant arm* abduction at shoulder) Normal/Abnormal. If Abnormal: 0/1/2/3/4

Biceps (non-dominant arm* flexion at elbow) Normal/Abnormal. If Abnormal: 0/1/2/3/4

Triceps (non-dominant arm* extension at elbows) Normal/Abnormal. If Abnormal: 0/1/2/3/4

Quadriceps (extension of the non-dominant* knee) Normal/Abnormal. If Abnormal: 0/1/2/3/4

Hamstrings (flexion of the non-dominant* knee) Normal/Abnormal. If Abnormal: 0/1/2/3/4

***Note: *If the subject was vaccinated on the dominant side, then this assessment should be done on the dominant side and the information should be recorded on the eCRF accordingly.* (Amended 09 June 2011)**

Deep tendon reflexes grading (normal will be fromminimal to slightly hyperactive ; abnormal will be no response and hyperactive with clonus/tetany. Biceps (non-dominant* elbow flexion) Normal/Abnormal. If abnormal : No response/Hyperactive with clonus, tetany

Patellar (non-dominant* knee extension) Normal/Abnormal. If abnormal : No response/Hyperactive with clonus, tetany

***Note: *If the subject was vaccinated on the dominant side, then this assessment should be done on the dominant side and the information should be recorded on the eCRF accordingly.* (Amended 09 June 2011)**

Sensitivity dermatome assessment: focus on the non-dominant arm

C5 Upper-outer arm (deltoid/biceps region, avoiding the immediate vaccination surrounding region) Normal/Abnormal. If Abnormal: hypoesthesia/hyperesthesiaanesthesia/paresthesia

C6 Thumb side of lower arm, palm side of thumb and index finger Normal/Abnormal. If Abnormal hypoesthesia/hyperesthesia /anesthesia/paresthesia

C7 Middle finger (both sides) and dorsal side of hands and fingers Normal/Abnormal. If Abnormal: hypoesthesia/hyperesthesiaanesthesia/paresthesia

C8 palm side of ring and pinky fingers Normal/Abnormal. If Abnormal: hypoesthesia/hyperesthesia /anesthesia/paresthesia

T1 Medial aspect of lower and upper arms Normal/Abnormal. If Abnormal: hypoesthesia/hyperesthesia /anesthesia/paresthesia

Cerebellar assessment:

Finger to nose Normal/Abnormal

Walk assessment:

Normal/Abnormal

Appendix C Laboratory assays

Any serum, plasma or blood cells not immediately used in immunological assays will be stored at -20°C or less for serum and in liquid nitrogen storage for blood cells, and would only be used to assess the immune response to vaccination or to malaria antigens, or to assess any potential toxicity of the vaccine. ***Serum for cytokine/chemokines analysis must be stored at -80°C or less.*** **(Amended 09 June 2011)**

All assays will be performed in a blinded manner without regard to the investigational group assignment of the volunteer subjects.

Hematological, biochemical and urine assays

Hematogical testing (WBC, haemoglobin, and platelets), biochemical testing (ALT, AST and creatinine) and urinalysis (protein, blood) will be measured at regular intervals throughout the study period as listed in Table 6.

Hematological, biochemical and urine testing will be performed at Quest or LabCorp laboratories using laboratory SOPs.

Humoral immunity assays

Blood for analysis of humoral immune response will be obtained from each volunteer at timepoints indicated in Table 6. After centrifugation, serum samples should be kept at – 20°C or less until shipment.

Antibody levels against CS (repeat region)

Antibody levels against *P. falciparum* CS-repeat region will be measured at GSK Biologicals by standard ELISA methodology using plate adsorbed recombinant polypeptide R32LR antigen. Anti-CS antibody levels will be determined relative to a standard reference antibody as a control according to SOPs from the laboratory. Results will be reported in EU/ml.

Antibody levels against HBsAg

Antibodies against hepatitis B surface antigen (HBsAg) will be measured at GSK Biologicals using an in-house validated ELISA assay. Results are expressed in mIU/ml and the cut-off for seroprotection is 10 mIU/ml.

Antibody levels against Ad35

Neutralizing antibody titers against adenovirus type 35 will be determined using the validated neutralizing antibody assay at the GLP laboratory at Crucell [Sprangers, 2003]. This functional assay determines the anti-Ad35 neutralizing antibody levels that inhibit infection of reporter cells. Results will be reported in 90% inhibiting concentration (IC90).

Antibody levels against CS (full-length and peptides other than repeat)

Standardized ELISA procedures established in the Clinical Immunology Laboratory to measure antibodies full-length recombinant CS protein will be performed using commercially available secondary antibody IgG1 reagents. Full-length recombinant PfCS will be GMP protein containing the full-length N- and C-terminal domains plus 19 NANP and 3 NVDP amino acid repeats from the 3D7 strain of *P. falciparum*.

Humoral assays to determine isotype, affinity, and epitope specificity

Human isotypes IgG1, IgG2, IgG3, and IgG4 against PfCS repeat peptide (40-mer) and full-length recombinant CS protein will be performed using commercially available secondary antibody isotype reagents. Affinity and avidity measurements will be conducted using standardized procedures using the Attana 200 instrument for analysis of molecular interactions from whole sera. The Attana 200 instrument is a dual channel, label-free, temperature controlled, continuous-flow system for automated determination of avidity and affinity of antibody to CS repeat peptides and to full-length recombinant CS protein. This instrument uses Quartz Crystal Microbalance (QCM) technology that allows for simultaneous kinetic on and off-rate characterization from crude serum samples. Epitope mapping of sera samples from subjects will be first tested in modified ELISA using overlapping biotinylated peptides captured onto streptavidin-coated microtiter plates spanning the entire CS sequence with further characterization of epitopes analyzed for avidity. Statistical correlations will be made to assess correlation with protection and delay to infection using a combination of affinity/avidity with mapped epitopes.

Cell-Mediated Immunity (CMI)

The role of cell-mediated immunity in the protective response to malaria is poorly understood. In addition to assessment of humoral immune responses, assays to analyze the cellular immune responses will also be performed. The cell-mediated immune response will be assessed on thawed PBMCs by Intracellular Cytokine Staining (ICS) at GSK Biologicals or a validated laboratory designated by GSK Biologicals and by ELISPOT at Crucell laboratory.

Detection of CS or HBs specific T-cells, ICS (Intracellular Cytokine Staining) technique

Intracellular staining for cytokines (ICS) is a method providing information on the frequency of CD4+ and CD8+ T cells responding to the antigen and secreting molecules involved in immunity such as IFN-γ, IL-2, TNF-a and CD40-L. The assay will be performed on thawed PBMCs. Briefly, PBMCs of the subjects are stimulated for 2 hours by the vaccine antigen (HBs and CS) and/or derived peptides. Then, intracellular block (Brefeldin A) is added to inhibit cytokine secretion for a subsequent overnight stimulation. The cells are then harvested and stained for cell surface markers (eg. CD4 and CD8), to identify specific sub-populations of T cells. Subsequently, the cells are fixed and then treated with a detergent such as saponin to permeabilize the cell membrane. The cells are then incubated with fluorescently labelled, cytokine-specific or activation marker-specific antibody that forms complexes with the cytokine in the

cytoplasm. The cytokine producing cells can then be detected by cytofluorometry. Results are expressed as a frequency of antigen-specific $CD4^+$ or $CD8^+$ T cells identified as expressing cytokine/marker upon the *in vitro* stimulation.

Detection of CS or Ad35-specific T-cells, ELISPOT technique:

The Human T-cell ELISPOT enumerates the number of IFN-γ producing T cells. This assay has been validated at the GLP laboratory at Crucell. A 15-mer overlapping peptide pool spanning the CS protein of high purity and extensive quality control has been designed to elicit cytokine production of CS specific T cells. Briefly, PBMC are thawed and rested overnight. The next day, cells are counted, plated in ELISPOT plates and stimulated overnight with CS peptide pool (2 µg/ml per peptide) or controls. After incubation, secreted IFN-γ is detected with alkaline phosphate conjugated detection antibody and stained with BCIP/NBT. Plates are air-dried and spots are counted with and AID ELISPOT counter. Responses are reported as spotforming units/10^6 cells.

Cytokine and chemokine measurement by Luminex Assay

Assessment of cytokines and chemokines in ***serum*** by particle based cytokine assay (Luminex®) at time points listed in Table 6. **(Amended 09 June 2011)**

Luminex® assays as standardized in commercially available kits (i.e. Invitrogen, Inc.) will be used to assess cytokine and chemokines in sera from subjects at predefined time points as indicated in the protocol. **(Amended 09 June 2011)** The assay will include those from a 30-plex kit for the simultaneous measurement of human IL-1β, IL-1RA, IL-2, IL-2R, IL-4, IL-5, IL-6, IL-7, IL-8, IL-10, IL-12 (p40⁄p70), IL-13, IL-15, IL-17, TNF-α, IFN-α, IFN-γ, GM-CSF, MIP-1α, MIP-1β, IP- More10, MIG, Eotaxin, RANTES, MCP-1, VEGF, G-CSF, EGF, FGF-basic, and HGF. Depending upon the results from the microarray transcriptional analysis, additional cytokines may be tested and obtained from commercially available sources. Flexibility is one of the many advantages of the Luminex® xMAP® multiplex platform. Luminex® assays are easy to set up and run, using a procedure that is very similar to an ELISA. We will use the maximum number of beads in each Luminex® assay, so that data are collected from at least 100 events per bead region per target. Counting at least 100 events, compared to the 50 recommended by some manufacturers, leads to lower % CVs and more precise assays. Luminex® assays are subject to rigorous quality control and validation prior to release.

Flow cytometry analysis for plasmablasts (FACS) and B cell ELISPOT

Flow cytometry will be used to evaluate the frequency and phenotype of circulating antibody-secreting cells, specifically $CD19^+$ $CD38^+$ $CD27^+$ plasmablasts at time points listed in Table 6. Plasmablasts, released from germinal centers appear transiently in the blood for several days post immunization before they home to the bone marrow. This cell type is considered critical for humoral memory and the persistence of antibodies. Since they do not survive the freeze/thaw process, this particular FACS staining will have to be performed on fresh cells. Further, to enumerate CS-specific cells that actively secrete antibodies, B cell ELISPOT analysis will be performed.

Gene expression profiling

Microarray Analyses

Microarray analysis will be performed using a 3-step strategy that will use the vaccinees from cohort A to define signatures that correlate with the antigen-specific immune responses, and protective efficacy, and then test the predictive capacity of such signatures using the vaccinees in cohorts B and C.

Step 1: To identify gene signatures that correlate with immunogenicity and protection in a "Training set", using vaccinees in "cohort A" (23 vaccinees per arm).

- Microarray analyses of PBMCs of 23 vaccinees per arm
- 23 vaccinees/arm x 2 arms x 1*4* time points (see Table 6) = 6*44* samples **(Amended 09 June 2011)**

In step 1, we will perform an intensive analysis of the kinetics of gene expression in all 46 vaccinees from cohort A to define the time point(s) at which the gene expression profiles best correlate with the ensuing antigen-specific antibody and T cell responses and protective immunity against malaria challenge. Gene expression analysis will be performed using the Affymetrix GeneChip Human Genome U133 Plus 2.0 Array. The analysis of gene expression data will be performed as described by Querec et al. [Querec, 2009].

Upon completion of cohort 1, there can be 2 possible outcomes:

1. Discontinuation of the study, for the reasons defined in the protocol. In this event, the primary end point of the systems biological analysis would have been to define signatures that correlate with antigen-specific immune responses and efficacy. A secondary end point would be to use a classification model such as the DAMIP model [Querec, 2009], a general-purpose optimization-based predictive modeling framework and computational engine, which is a very powerful supervised-learning classification approach in predicting various biomedical and biobehavioral phenomena. We will train the DAMIP model using cohort 1 to obtain an unbiased estimate of correct classification (between high and low immune responders, or between vaccinees who acquired the disease versus those who did not), within cohort 1.
2. Continuation of the study with analysis of cohorts B and C. This will allow us to test the predictive capacity of the signatures defined in cohort A, in an independent set of vaccinees in cohorts B and C. Therefore, in the following step 2, we will evaluate gene expression profiles on the time points identified as being most informative as per our analysis in step 1, with a view to determining whether such signatures can predict the antigen-specific antibody and T cell responses and efficacy, in the independent set of vaccinees in cohorts B and C. Therefore, we will first train the DAMIP model using trial 1 to obtain an unbiased estimate of correct classification, and then use cohort B as a blind test to predictive capacity of the signature.

Step 2: To test the predictive capacity of the signatures identified in cohort A using a "Test set 1" in cohort B

- Microarray analyses of PBMCs of 23 vaccinees per arm
- 23 vaccinees/arm x 2 arms x 4 time points = 184 samples for “Test set 1” (cohort B). We will only study the time points that were identified as being most informative in our analysis of cohort A.
- Total number of samples for microarrays in Test 1 (cohort B) = 184

If the signature defined in cohort 1 is capable of predicting the immunogenicity and efficacy in cohort B with high accuracy (e.g. >90%, as was the case with the yellow fever study [Querec, 2009], then we will further validate this signature using cohort C, as described below. If however, the predictive capacity is less than optimal, then we will treat cohorts A and B as a single dataset, by pooling the datasets from both cohorts, with a view to increasing the sample size and achieving greater statistical robustness in the analysis. Then we will test the predictive capacity of this signature (from the pooled cohort A and B analysis), using cohort C as a test set, as defined below.

Step 3: To test the predictive capacity of the signatures identified in cohort A (and cohort B) using a “Test set 2,” in cohort C

- Microarray analyses of PBMCs of 23 vaccinees per arm
- 23 vaccinees/arm x 2 arms x 4 time points = 184 samples for “Test set 2” (cohort C)
- Total number of samples for microarrays in Test 2 (cohort C) = 184

Total number of samples for microarrays = 690 (cohort A) + 184 (cohort B) + 184 (cohort C) = 1058

Deep sequencing

Deep sequencing (mRNA-Seq) is a technique using next generation sequencing technology to sequence and quantify mRNA samples. It is more sensitive then microarray analysis allowing to measure very small changes in gene expression. It is to do with analysis of transcription, not genetic testing. The Illumina Genome AnalyzerII uses clonal array formation and reversible terminator technology to generate 20-30 million short sequence reads per sample lane. These short sequences are aligned with the genome and counted to provide the numerical frequency of sequences in the library population. This technique reveals much more information about the transcriptome than the hybridization-based technologies. In addition to identifying transcripts and determining relative expression levels, sequencing the entire mRNA population uncovers novel transcripts and isoforms, alternative splice sites, information regarding gene promoters and rare transcripts. The experiments will be instructed by the results obtained from the microarray studies.

Determination of parasitemia

Blood samples for smear and PCR will be taken during the challenge phase at the time points listed in Table 6. Detection and quantification of *P. falciparum* parasitemia will be made at the investigator’s sites according to laboratory SOPs.

Appendix D Amendments and administrative changes to the protocol

GlaxoSmithKline Biologicals<br>Clinical Research & Development<br>**Protocol Amendment 1**	
**eTrack study number and Abbreviated Title**	114460 (Malaria-068)
**IND number**	***BB-IND-14701***
**Amendment number:**	Amendment 1
**Amendment date:**	09 June 2011
**Co-ordinating author:**	Liliana Manciu, Scientific Writer

Rationale/background for changes:

In order to reduce the risk of high reactogenicity reactions and in line with a FDA recommendation, the Ad35.CS.01 dose for injection was lowered from $1x10^{11}$ vp to $5x10^{10}$ vp in Table 21.

In addition, the following minor changes have been included:

- The IND number has been added on the cover Title page, Sponsor and Investigator Signatory pages.
- Blood sampling for smear and PCR was deleted at Visit 41 in Synopsis Table 4, Table 6. Consequently, the volume of blood to be collected at Visits 41 and 42 has been corrected throughout the protocol (i.e. Synopsis Table 4, Tables 6, 10 and 13).
- The reference to Table 27 has been corrected to Table 28 in Section 4.4. In addition, a note has been added to clarify that NHANES I criteria will be applied for all subjects including subjects aged 18-35 years old.
- The trade mark for Malarone has been added in Section 5.4.2.2.
- Information concerning timings post-challenge for a volunteer who develops malaria and has 3 consecutive negative smears following initial treatment has been corrected in Section 5.4.3.5 and Table 9.
- Information on weekly evaluations after malaria treatment has been deleted in Section 5.4.3.6 as these weekly visits are usually not conducted as part of the standard sporozoite challenge model.

- One re-bleeding during screening, as an acceptable way to refine or confirm whether a potential participant is eligible or not, or to complete an insufficient blood volume is allowed, when medically justified, at investigators discretion. Examples include a clotted or hemolysed sample, or transient borderline abnormal blood values considered non-clinically significant. This information has been added in Table 7.
- One re-bleeding at each time point that a blood sample is taken during the study, after the screening epoch, is also allowed, if necessary, at investigators discretion, to cover for technical issues (insufficient blood volume, clotted sample, hemosysis…). This information has been added in Tables 8, 9 and 10.
- One urine re-sampling is allowed, if necessary, at each time point that a urine sample is taken during the study, at investigators discretion, to cover for technical issues and guarantee a clean urine catch. This information has been added in Tables 7 and 8.
- Information on the follow-up period for non-serious AEs has been deleted in Table 8.
- The order in which study procedures related to the assignment of study subject number and the check of inclusion/exclusion criteria are presented has been switched in Table 7 and Section 5.6.2 to better reflect what will actually be done.
- Information on cytokine/chemokines analysis has been corrected throughout the protocol (i.e. Section 5.6.2.8, Table 15 and Appendix C) as this analysis will be performed on serum instead of plasma.
- Information concerning the temperature storage for the serum to be used for cytokine/chemokines analysis has been modified from -20°C to -80°C in Section 5.6.2.8 and Appendix C.
- Section 5.6.3.9 has been modified with the purpose to standardize reactogenicity data collection on the diary cards.
- The laboratory performing deep sequencing has been updated in Table 16 from ISB to Seattle Biomed.
- Table 20 has been modified to add Visit 16 (D57) timepoint which was erroneously missing.
- Information on the diluent to be used to dilute Ad35.CS.01 to the intended dose for the study ($5x10^{10}$ vp instead of $1x10^{11}$ vp per injected dose) has been added in Table 21 and Sections 6.2.2 and 6.3.2.2.
- Temperature deviation for the Ad35.CS.01 vaccine has to be reported to the sponsor. Once the sponsor becomes aware of a temperature deviation, he must inform Crucell IMMEDIATELY. This information has been corrected in Section 6.2.2.
- The time window for recording concomitant medication has been corrected in Section 6.7.6.2 to extend it to the screening period (as specified in Table 7).

- Information concerning the number of timepoints for microarray assays has been corrected in Appendix C from 15 to 14 timepoints (as specified in Table 6).
- Minor corrections such as clarifications and typos have been made Appendix B, Section 5.4.2.1.

Amended text has been included in *bold italics* in the following sections. Deleted text is shown with ~~strikethrough~~.

Title page, Sponsor Signatory Page, Investigator Signatory Page

**Investigational New Drug (IND) number**	***BB-IND-14701***

Synopsis Table 4 and Table 6: Sampling timepoints and volume of blood collected

**Epoch**	**Challenge**																					**Follow-up**	
	**DoC***	**Early Post-challenge**					**Hotel/inpatient Phase**										**Late Post-challenge**					**Final Visits**	
**Study day**	**77**	**78**	**82**	**83**	**84**	**85**	**86**	**87**	**88**	**89**	**90**	**91**	**92**	**93**	**94**	**95**	**97**	**99**	**101**	**103**	**105**	**140**	**236**
**Challenge day**	**0**	**1**	**5**	**6**	**7**	**8**	**9**	**10**	**11**	**12**	**13**	**14**	**15**	**16**	**17**	**18**	**20**	**22**	**24**	**26**	**28**	**63**	**159**
**Visit number**	**20**	**21**	**22**	**23**	**24**	**25**	**26**	**27**	**28**	**29**	**30**	**31**	**32**	**33**	**34**	**35**	**36**	**37**	**38**	**39**	**40**	**41**	**42**
Safety labs (7 ml)**	7													7***							7	7	7
Smear and PCR (2 ml)			2	2	2	2	2	2	2	2	2	2	2	2	2‡	2‡	2‡	2‡	2‡	2‡	2‡	~~2‡~~	
Humoral immunity (ELISA) (20 ml)	20																				20	20	20
Cellular immunity (60 mL)	60																				60	60	60
Exploratory cytokine/chemokine analysis (Luminex) (2 ml)	2	2	2																		2		
Microarray and deep sequencing analysis (12 ml)	12	12	12																				
**Blood volume in ml per visit**	**101**	**14**	**16**	**2**	**2**	**2**	**2**	**2**	**2**	**2**	**2**	**2**	**2**	**9****	**2**	**2**	**2**	**2**	**2**	**2**	**91**	**87~~9~~**	**87**
Cumulative blood vol. - Immunized groups (ml)	609	623	639	641	643	645	647	649	651	653	655	657	659	668	670	672	674	676	678	680	771	858~~60~~	945~~7~~
Cumulative blood vol. – Infectivity controls (ml)	123	137	153	155	157	159	161	163	165	167	169	171	173	182	184	186	188	190	192	194	285	3724	459~~61~~

*DoC: day of challenge. **Safety labs include hemoglobin, WBC, PLT, ALT, AST, creatinine. ***Day of parasitemia and number of parasitemic individuals is undetermined.

‡ For volunteers who develop malaria: blood smears and PCR may be discontinued once the volunteer has 3 consecutive (separated by greater than 12 hours) negative smears following initial treatment.

Section 4.4. Exclusion criteria for enrollment

- Any abnormal baseline laboratory screening tests: ALT, AST, creatinine, hemoglobin, platelet count, total white blood cell count, out of normal range as defined in the protocol ***Table 28*** ~~Table 27~~.
- Evidence of increased cardiovascular disease risk, "moderate" or "high", according to the NHANES I criteria (see Appendix A).

  Note: NHANES I criteria will be applied for all subjects including subjects aged 18-35 years old.

Section 5.4.2.1. Risks associated with vaccination and mitigation of risks

Results from an anti-HIV vaccine trial using an adenovirus type 5 (Ad5) vaccine expressing HIV antigens (ST***E***P trial) tested in 3,000 people was interrupted early because rather than being protective an increased incidence of HIV infections was seen in vaccinated individuals as compared to placebo.

Section 5.4.2.2. Risks associated with malaria challenge and mitigation of risks

Chloroquine phosphate and, if required, other appropriate FDA-approved drugs, such as quinine, doxycycline and **M**~~m~~alarone® currently available in the U.S., will be used in standard doses to treat volunteers who become infected with malaria.

Section 5.4.3.5. Management of Infected Human Volunteers

A volunteer who develops malaria and has 3 consecutive (separated by greater than 12 hours) negative smears following initial treatment may be excused from ***the remaining hotel/inpatient facility visits and*** the late post-challenge clinic visits at Study Day ***97, 99, 101 and 103 (***20, 22, 24~~,~~ ***and*** 26 ***days post-challenge)*** but will be required to come to the clinic center at Study Day 105 (28 days post- challenge). Telephone contact will be made if the volunteer does not keep a scheduled follow-up appointment.

Section 5.4.3.6. Malaria Treatment

It is anticipated that treatment of malaria will be curative, since relapses do not occur after adequate treatment of *P. falciparum* infections. No previous volunteer infected and treated by WRAIR has had a malaria relapse. ~~Nonetheless, once weekly evaluations will be performed for at least four weeks after treatment to evaluate the unlikely possibility of recrudescent infection. In addition, s~~***S***ubjects will be advised to contact the study physician, or to advise their personal physician of their participation in this malaria study, if fever, headache, or other symptoms possibly related to malaria develop at any time within one year after completion of the study.

Section 5.5 Outline of study procedures

Table 7. List of study procedures – Screening epoch (vaccinees and infectivity controls)

Epoch	Screening epoch	
**Study visit**	**1**	**2¶**
**Study day**	**-90 to -3**	**-7 to -1**
~~Assign subject number~~	~~●~~	
Check inclusion/exclusion criteria	●	●
Assign subject number	●	
Blood sampling for safety analysis (7 mL)	●[§, v]	
Blood sampling for additional screening analysis (15 mL)	●[*, v]	
Blood sampling for humoral immunity (20 mL)		●[v]
Blood sampling for CMI (60 mL)		●[v]
Blood sampling for exploratory cytokine/chemokine Luminex (2 mL)		●[v]
Blood sampling for microarray and deep sequencing analysis (12 mL)		●[v]
Blood sampling for plasmablasts FACS and B cell ELISPOT (10 mL)		●[v]
**Total blood volume in mL per visit**	**22**	**104**
Cumulative blood volume – Immunization groups (mL)	22	126
Cumulative blood volume – Infectivity controls (mL)	22	-

● is used to indicate a study procedure that requires documentation in the individual eCRF
○ is used to indicate a study procedure that does not require documentation in the individual eCRF
¶Study visit **NOT** applicable for the infectivity controls
§ Safety labs include hemoglobin, WBC, PLT, ALT, AST, creatinine
*Additional screening analysis include HIV, HCV, HBV
[v]One repeat blood draw to refine or confirm whether a potential participant is eligible or not, or to complete an insufficient blood volume is allowed during the screening, when medically justified, at investigators discretion.

Table 8 List of study procedures - Vaccination epoch (vaccinees)

Epoch	Vaccination epoch																
**Study visit**	**3**	**4**	**5**	**6**	**7**	**8**	**9**	**10**	**11**	**12**	**13**	**14**	**15**	**16**	**17**	**18**	**19**
**Study day**	**0**	**1**	**2**	**3**	**6**	**14**	**28**	**29**	**30**	**31**	**34**	**42**	**56**	**57**	**58**	**59**	**62**
Recording of non-serious AEs ~~within 30 days post-vaccination~~	●	●	●	●	●	●	●	●	●	●	●	●	●	●	●	●	●
Urine analysis for safety**					●		●										
Blood sampling for safety analysis (7 mL)[z]	● §				● §		● §				● §		● §				● §
Blood sampling for humoral immunity (20 mL)[z]							●						●				
Blood sampling for CMI (60 mL)[z]						●						●					
Blood sampling for exploratory cytokine/chemokine Luminex (2 mL)[z]		●	●		●	●	●	●			●		●	●			●
Blood sampling for microarray and deep sequencing analysis (12 mL)[z]		●	●		●	●	●	●			●		●	●			●
Blood sampling for plasmablasts FACS and B cell ELISPOT (10 mL)[z]							●				●		●				●

● is used to indicate a study procedure that requires documentation in the individual eCRF
○ is used to indicate a study procedure that does not require documentation in the individual eCRF
§ Safety labs include hemoglobin, WBC, PLT, ALT, AST, creatinine
One repeat urine sampling is allowed, if necessary, at each time point that a urine sample is taken during the study, at investigators discretion.
[z]***One repeat blood drawn is allowed, if necessary, at each time point that a blood sample is taken during the study, at investigators discretion.***

Table 9 List of study procedures - Challenge epoch (vaccinees and infectivity controls)

Epoch	Challenge																			
Challenge phase	DoC†	Early post-challenge					Hotel/inpatient phase[v]										Late post-challenge[v]				
Study visit	20	21	22	23	24	25	26	27	28	29	30	31	32	33	34	35	36	37	38	39	40
Study day	77	78	82	83	84	85	86	87	88	89	90	91	92	93	94	95	97	99	101	103	105
Challenge day	0	1	5	6	7	8	9	10	11	12	13	14	15	16	17	18	20	22	24	26	28
Blood sampling for PCR and smear (2mL)[z]			●	●	●	●	●	●	●	●	●	●	●	●	●	●	● ‡	● ‡	● ‡	● ‡	● ‡
Blood sampling for safety analysis (7 mL)[z]	● §													● # §							● §
Blood sampling for humoral immunity (20 mL)[z]	●																				●
Blood sampling for CMI (60 mL)[z]	●																				●
Blood sampling for exploratory cytokine/chemokine Luminex (2 mL)[z]	●	●	●																		●
Blood sampling for microarray and deep sequencing analysis (12 mL)[z]	●	●	●																		

§ Safety labs include hemoglobin, WBC, PLT, ALT, AST, creatinine

† DoC: Day of challenge

Day of parasitemia and number of parasitemic individuals is undetermined

‡ For volunteers who develop malaria: blood smears and PCR may be discontinued once the volunteer has 3 consecutive (separated by greater than 12 hours) negative smears following initial treatment.

[v] A volunteer who develops malaria and has 3 consecutive (separated by greater than 12 hours) negative smears following initial treatment may be excused from the remaining hotel/inpatient facility visits and the late post-challenge clinic visits at Study Day ***97, 99, 101 and 103*** (20, 22, 24 ***and*** 26 ***days post-challenge***)

[w] An interim analysis for futility will be performed on safety and efficacy data collected up to 28 days post-challenge (Study Day 105) for the first 46 subjects vaccinated and challenged (Cohort A). A primary analysis will be performed on all efficacy, immunogenicity and safety data collected up to 28 days post-challenge (Study Day 105) on all cohorts.

[z] One repeat blood drawn is allowed, if necessary, at each time point that a blood sample is taken during the study, at investigators discretion.

● is used to indicate a study procedure that requires documentation in the individual eCRF

○ is used to indicate a study procedure that does not require documentation in the individual eCRF.

Table 10 List of study procedures - Follow-up epoch (vaccinees and infectivity controls)

**Epoch**	**Follow-up epoch**	
**Study visit**	**41**	**42**
**Study day**	**140**	**236**
**Challenge day**	**63**	**159**
Physical examination	O	O
Reporting of SAEs	●	●
Reporting of pregnancies	●	●
Record any concomitant medication/vaccination	●	●
Record any intercurrent medical condition	●	●
Blood sampling for safety analysis (7 mL)[z]	● §	● §
Blood sampling for humoral immunity (20 mL)[z]	●	●
Blood sampling for CMI (60 mL)[z]	●	●
**Total blood volume in mL per visit**	**87~~9~~**	**87**
Cumulative blood volume – Immunization groups (mL)	8***58***~~60~~	94***5***~~7~~
Cumulative blood volume – Infectivity controls (mL)	37**2**~~4~~	4***59***~~61~~
Study conclusion		●

● is used to indicate a study procedure that requires documentation in the individual eCRF
○ is used to indicate a study procedure that does not require documentation in the individual eCRF
§ Safety labs include hemoglobin, WBC, PLT, ALT, AST, creatinine
[z]One repeat blood drawn is allowed, if necessary, at each time point that a blood sample is taken during the study, at investigators discretion.

Section 5.6.2.8. Blood sampling for safety and immune response assessments

<u>Subjects in the immunization groups only</u>

- A volume of at least 2 mL of whole blood should be drawn only for exploratory cytokine/chemokines analysis by Luminex. After centrifugation, ***serum*** ~~plasma~~ samples should be kept at ~~–2~~*7*0°C or less until testing.

Section 5.6.2. Procedures during the screening epoch

~~**Section 5.6.2.1 Assign subject number**~~

~~Subject number will be assigned at the screening visit 1.~~

Section *5.6.2.1* ~~5.6.2.2.~~Check inclusion and exclusion criteria

Check all applicable inclusion and exclusion criteria as described in Sections 4.3 and 4.4 before enrollment.

Section 5.6.2.2 Assign subject number

Subject number will be assigned at the screening visit 1.

Section 5.6.3.9. Recording of AEs, SAEs and pregnancies

~~Verification~~ ***Completion*** of diary cards will be done with the investigator on days 1, 2, 3 and 6 after each vaccination.

Section 5.7.2. Biological samples

Table 13. Biological samples

Sample type	Quantity	Unit	Timepoint	Nr. subjects*
Blood	87~~9~~	ml	Visit 41 (D140)	168 (+12 to 18§)

*Estimated number of subjects
§Four to six infectivity controls will be enrolled per challenge day for each of the three cohorts.

Section 5.7.3. Laboratory assays

Table 15. Cell-Mediated Immunity (CMI)

System	Component	Challenge	Method	Unit	Laboratory
Serum ~~Plasma~~	Cytokines and chemokines**	-	30 plex cytokine/chemokine-Invitrogen (Luminex)**	MFV (mean fluorescent value)	WRAIR

† or validated laboratory designated by GSK Biologicals.
$ other exploratory markers of CMI may be assessed.
*or equivalent
**exploratory
***Fresh PBMC
ICS = Intracellular cytokine staining. PBMC = Peripheral blood mononuclear cells
PBMC = Peripheral blood mononuclear cells

Table 16. Gene expression profiling as part of a systems biology approach

System	Component	Method	Kit / Manufacturer	Unit	Laboratory
PBMC	mRNA	Microarray*	Affymetrix GeneChip Human Genome U133 Plus 2.0 Array	Gene expression	Emory University
PBMC	mRNA	Deep sequencing*	Illumina Genome Analyzer *II* array	Gene expression	***Seattle Biomed***~~ISB~~

*exploratory.
PBMC = Peripheral blood mononuclear cells

Section 5.7.4.1. Blood testing plan

Table 20. Summary of blood sampling time points and laboratory assays

Blood sampling timepoint		Component	No. subjects*	Priority ranking
Type of contact and timepoint	Sampling timepoint			
Visit 16 (D57)	***PIII(D57)***	***Safety analysis***		
		Exploratory cytokine (Luminex)	***168***	***1***
		Microarray and deep sequencing	***168***	***2***

Section 6.1 Study vaccines

Table 21 Study vaccines

Treatment name	Vaccine/ product name	Formulation	Presentation	***Injectable*** ~~V~~**volume**	Number of doses
Ad35.CS.01	Ad35.CS.01	$2x10^{11}$ Ad35.CS.01 vp/mL, Tris, NaCl, $MgCl_2$, PolySorbate-80, Sucrose, EDTA, Histidine, Ethanol	Clear to slightly opalescent and colourless solution with no visible particles in type I glass vial	0.5 ml[1, 2]	1
	Diluent	***Tris, NaCl, $MgCl_2$, PolySorbate-80, Sucrose, EDTA, Histidine, Ethanol***	***Clear liquid solution in type I glass vial***		***1***
RTS,S/$AS01_B$	RTS,S	RTS,S antigen: 50 µg RTS,S with sucrose as cryoprotectant	RTS,S antigen: Lyophilized pellet in single dose vial to be reconstituted with $AS01_B$ adjuvant in liquid form	0.5 ml[3]	2/3
	$AS01_B$	$AS01_B$ adjuvant: 50 microgrammes of MPL and 50 microgrammes of Stimulon QS21 (a triterpene glycoside purified from the bark of Quillaja saponaria) with liposomes	$AS01_B$ adjuvant: liquid solution in monodose glass vial		

1 Volume after dilution

2 One dose of 0.5 ml after dilution will contain $5x10^{10}$ Ad35.CS.01 vp.

3 Volume after reconstitution

Note: Due to ***potential visible*** ~~volume differences and potential color~~ differences between the reconstituted RTS,S/$AS01_B$ and the Ad35.CS.01 vaccine, dedicated unblinded staff in each of the investigational sites will be accountable for the reconstitution and administration ***of the vaccine*** to a subject.

Section 6.2.2 Ad35.CS.01 vaccine (in Section 6.2 Storage and handling of study vaccines)

The Ad35.CS.01 vaccine must be stored at the defined temperature range (i.e. ≤-65°C/≤-85°F). ***The diluent must be stored at 2-8°C/36 to 46°F.*** Please refer to the Module on Clinical Trial Supplies in the SPM for more details on storage of the study vaccines. The storage temperature of the vaccine will be monitored daily with temperature monitoring device(s) (at a minimum calibrated) and will be recorded as specified in the SPM.

The storage conditions will be assessed during pre-study activities under the responsibility of the sponsor study contact.

Any temperature deviation >-65°C/-85°F ***for the Ad35.CS.01 vaccine or outside the range 0 to 8°C/32 to 46°F for the diluent*** must be reported to ***the sponsor*** ~~Crucell~~ as soon as detected. ***Once the sponsor becomes aware of a temperature deviation, he must inform Crucell IMMEDIATELY.*** Following an exposure to such a temperature deviation, ***the Ad35.CS.01*** vaccine~~s~~ ***and the diluent*** will not be used until approval has been given by Crucell ***via the sponsor***.

Refer to the Module on Clinical Trial Supplies in the SPM for details and instructions on the Temperature deviation process, packaging and accountability of the Ad35.CS.01 vaccine ***and diluent***.

Section 6.3.3.2 Ad35.CS.01 vaccine (in Section 6.3 Injection instructions)

The Ad35.CS.01 vaccine will be supplied ~~in~~ ***as a*** single use, ***3 mL*** clear ***type I*** glass vial~~s~~ containing ~~frozen liquid product~~ ***0.7 mL vaccine (0.5 mL extractable).*** ~~One 0.5 mL dose will be withdrawn from each vial and used.~~ ***The diluent to be used to dilute the Ad35.CS.01 vaccine to the intended dose for the clinical study is supplied as a single use, 3 mL clear type I glass vial containing 1.4 mL solution (1.0 mL extractable).***

The Ad35.CS.01 vaccine storage condition is ≤-65°C/-85°F. The Ad35.CS.01 vaccine will be thawed at room temperature (15-25°C) ~~for a period of one to two hours~~ prior to dose preparation. When thawed, the liquid material appears as a clear to slightly opalescent solution.

~~Prior to withdrawing the dose of Ad35.CS.01, the vial will be gently swirled and inspected for particulate matter. Remove flip-off cap from the vial and disinfect the rubber closure.~~

~~A maximum 2-hour time period at room temperature is allowed between the time the Ad35.CS.01 vaccine is drawn into the syringe and vaccine administration. The vaccine should be kept at room temperature and should be administrated by~~ ***Each dose (per study protocol) of the vaccine (0.5 mL) will be administered with a sterile, disposable syringe and needle by*** IM injection into the deltoid muscle of the non-dominant arm except for variances like tattoos, rashes, burns or other skin disorders subject to the discretion of the investigator, using a fresh needle.

Detailed instructions for the preparation, dilution and dispensation of the assigned dose of the investigational product will be provided separately in the Pharmacy Manual.

Section 6.7.6.2. Time window for recording concomitant medication/vaccination in the eCRF

All concomitant medications, with the exception of vitamins and/or dietary supplements, administered at ANY time during the period starting with the ***first screening visit*** ~~administration of the first dose of study vaccine~~ and ending 6 months after last dose of study vaccine must be recorded in the eCRF.

Appendix B Neurological examination form

Muscle strength grading (Normal: muscle can move in full range of motion, against full resistance. Abnormal: 0 Complete paralysis, no visible movement ; 1 Trace strength, some visible muscle contraction, with no movement of joint ; 2 poor strength, muscle can move the joint but not against gravity ; 3 Fair strength, muscle can move against gravity, but not against any examiner resistance ; 4 Good strength, muscle can move against moderate resistance, but not examiner full resistance).

Deltoid (non-dominant arm* abduction at shoulder) Normal/Abnormal. If Abnormal: 0/1/2/3/4

Biceps (non-dominant arm* flexion at elbow) Normal/Abnormal. If Abnormal: 0/1/2/3/4

Triceps (non-dominant arm* extension at elbows) Normal/Abnormal. If Abnormal: 0/1/2/3/4

Quadriceps (extension of the non-dominant* knee) Normal/Abnormal. If Abnormal: 0/1/2/3/4

Hamstrings (flexion of the non-dominant* knee) Normal/Abnormal. If Abnormal: 0/1/2/3/4

Note: *If the subject was vaccinated on the dominant side, then this assessment should be done on the dominant side and the information should be recorded on the eCRF accordingly.

Deep tendon reflexes grading (normal will be fromminimal to slightly hyperactive ; abnormal will be no response and hyperactive with clonus/tetany. Biceps (non-dominant* elbow flexion) Normal/Abnormal. If abnormal : No response/Hyperactive with clonus, tetany

Patellar (non-dominant* knee extension) Normal/Abnormal. If abnormal : No response/Hyperactive with clonus, tetany

Note: *If the subject was vaccinated on the dominant side, then this assessment should be done on the dominant side and the information should be recorded on the eCRF accordingly.

Appendix C laboratory assays

Any serum, plasma or blood cells not immediately used in immunological assays will be stored at -20°C or less for serum and in liquid nitrogen storage for blood cells, and would only be used to assess the immune response to vaccination or to malaria antigens, or to assess any potential toxicity of the vaccine. ***Serum for cytokine/chemokines analysis must be stored at -70°C or less.***

Cytokine and chemokine measurement by Luminex Assay

Assessment of cytokines and chemokines in ***serum*** ~~plasma~~ by particle based cytokine assay (Luminex®) at time points listed in Table 6.

Luminex® assays as standardized in commercially available kits (i.e. Invitrogen, Inc.) will be used to assess cytokine and chemokines in sera~~/plasma~~ from subjects at predefined time points as indicated in the protocol.

Gene expression profiling

Microarray Analyses

<u>Step 1:</u> To identify gene signatures that correlate with immunogenicity and protection in a "Training set", using vaccinees in "cohort A" (23 vaccinees per arm).

- Microarray analyses of PBMCs of 23 vaccinees per arm
- 23 vaccinees/arm x 2 arms x 1***4***~~5~~ time points (see Table 6) = 6***44***~~90~~ samples
