# Supplementary material for: Ad35.CS.01 - RTS,S/AS01 Heterologous Prime Boost Vaccine Efficacy against Sporozoite Challenge in Healthy Malaria-Naïve Adults
Source: PLoS One. 2015 Jul 6;10(7):e0131571. doi: 10.1371/journal.pone.0131571 (PMC4492580; doi:10.1371/journal.pone.0131571)
Supplement: S1 Table — (DOCX) [file pone.0131571.s004.docx]

**S1 Table. Unsolicited symptoms following dose 1**

|  | | **ARR  N = 28** | | | | **RRR  N = 27** | | | |
| --- | --- | --- | --- | --- | --- | --- | --- | --- | --- |
|  | |  | | **95% CI** | |  | | **95% CI** | |
| **Primary System Organ Class** | **Preferred Term** | **n** | **%** | **LL** | **UL** | **n** | **%** | **LL** | **UL** |
| At least one symptom |  | 11 | 39.3 | 21.5 | 59.4 | 4 | 14.8 | 4.2 | 33.7 |
| Blood and lymphatic system disorders | Lymphadenopathy | 1 | 3.6 | 0.1 | 18.3 | 0 | 0.0 | 0.0 | 12.8 |
| Gastrointestinal disorders | Oral pain | 1 | 3.6 | 0.1 | 18.3 | 0 | 0.0 | 0.0 | 12.8 |
| General disorders and administration site conditions | Malaise | 1 | 3.6 | 0.1 | 18.3 | 0 | 0.0 | 0.0 | 12.8 |
|  | Pain | 3 | 10.7 | 2.3 | 28.2 | 0 | 0.0 | 0.0 | 12.8 |
| Hepatobiliary disorders | Hepatitis | 0 | 0.0 | 0.0 | 12.3 | 1 | 3.7 | 0.1 | 19.0 |
| Infections and infestations | Nasopharyngitis | 1 | 3.6 | 0.1 | 18.3 | 1 | 3.7 | 0.1 | 19.0 |
|  | Tonsillitis | 1 | 3.6 | 0.1 | 18.3 | 0 | 0.0 | 0.0 | 12.8 |
|  | Upper respiratory tract infection | 1 | 3.6 | 0.1 | 18.3 | 0 | 0.0 | 0.0 | 12.8 |
|  | Urinary tract infection | 1 | 3.6 | 0.1 | 18.3 | 0 | 0.0 | 0.0 | 12.8 |
| Musculoskeletal and connective tissue disorders | Back pain | 2 | 7.1 | 0.9 | 23.5 | 0 | 0.0 | 0.0 | 12.8 |
|  | Musculoskeletal stiffness | 1 | 3.6 | 0.1 | 18.3 | 0 | 0.0 | 0.0 | 12.8 |
|  | Neck pain | 0 | 0.0 | 0.0 | 12.3 | 1 | 3.7 | 0.1 | 19.0 |
| Nervous system disorders | Dysgeusia | 1 | 3.6 | 0.1 | 18.3 | 0 | 0.0 | 0.0 | 12.8 |
|  | Hypoaesthesia | 1 | 3.6 | 0.1 | 18.3 | 0 | 0.0 | 0.0 | 12.8 |
|  | Paraesthesia | 1 | 3.6 | 0.1 | 18.3 | 0 | 0.0 | 0.0 | 12.8 |
| Reproductive system and breast disorders | Dysmenorrhoea | 1 | 3.6 | 0.1 | 18.3 | 0 | 0.0 | 0.0 | 12.8 |
| Respiratory, thoracic and mediastinal disorders | Oropharyngeal pain | 0 | 0.0 | 0.0 | 12.3 | 1 | 3.7 | 0.1 | 19.0 |
| Skin and subcutaneous tissue disorders | Hyperhidrosis | 0 | 0.0 | 0.0 | 12.3 | 1 | 3.7 | 0.1 | 19.0 |
|  | Pruritus | 1 | 3.6 | 0.1 | 18.3 | 0 | 0.0 | 0.0 | 12.8 |
