# Supplementary material for: Ad35.CS.01 - RTS,S/AS01 Heterologous Prime Boost Vaccine Efficacy against Sporozoite Challenge in Healthy Malaria-Naïve Adults
Source: PLoS One. 2015 Jul 6;10(7):e0131571. doi: 10.1371/journal.pone.0131571 (PMC4492580; doi:10.1371/journal.pone.0131571)
Supplement: S2 Table — (DOCX) [file pone.0131571.s005.docx]

## S2 Table. Frequency of CS-specific CD8+ T-cells expressing at least 2 cytokines/activation markers between IL- 2, IFN-γ, TNF-a and CD40L, per million PBMC (ATP cohort for immunogenicity)

|  | ARR | | | RRR | | | |
| --- | --- | --- | --- | --- | --- | --- | --- |
| TIMING | GM | 95% CI (LL) | 95% CI (UL) | GM | 95% CI (LL) | 95% CI (UL) | p-value |
| Day 0 | 7.1 | 3.2 | 15.8 | 2.8 | 1.3 | 6.3 | 0.1129 |
| Day 14 (Post 1) | 5.6 | 2.5 | 12.6 | 3.2 | 1.6 | 6.3 | 0.3205 |
| Day 42 (Post 2) | 14.1 | 5 | 39.8 | 4.8 | 2 | 10 | 0.0889 |
| Day 77 (Post 3) | 25.7 | 10 | 63.1 | 9.8 | 4 | 25.1 | 0.1584 |
| Day 105 (Post 3) | 31.6 | 10 | 100 | 6.6 | 2 | 20 | 0.0618 |
| Day 140 (Post 3) | 17.4 | 5 | 50.1 | 6.6 | 2.5 | 15.8 | 0.1666 |
| Day 236 (Post 3) | 10 | 3.2 | 31.6 | 4.5 | 1.6 | 12.6 | 0.2786 |
|  | Not Protected | | | Protected | | | |
| TIMING | GM | LL | UL | GM | LL | UL | p-value |
| Day 0 | 6.5 | 2.5 | 15.8 | 4 | 1.6 | 10 | 0.462 |
| Day 14 (Post 1) | 5.5 | 2 | 15.8 | 3.6 | 1.6 | 7.9 | 0.5037 |
| Day 42 (Post 2) | 11.2 | 4 | 31.6 | 7.2 | 2.5 | 20 | 0.5287 |
| Day 77 (Post 3) | 22.9 | 7.9 | 63.1 | 11.2 | 4 | 31.6 | 0.3181 |
| Day 105 (Post 3) | 14.5 | 4 | 50.1 | 16.2 | 4 | 63.1 | 0.8881 |
| Day 140 (Post 3) | 11.5 | 4 | 39.8 | 11.2 | 3.2 | 39.8 | 0.9703 |
| Day 236 (Post 3) | 10.5 | 3.2 | 31.6 | 4.6 | 1.3 | 15.8 | 0.2907 |

GM; Geometric mean
