# Supplementary material for: Ad35.CS.01 - RTS,S/AS01 Heterologous Prime Boost Vaccine Efficacy against Sporozoite Challenge in Healthy Malaria-Naïve Adults
Source: PLoS One. 2015 Jul 6;10(7):e0131571. doi: 10.1371/journal.pone.0131571 (PMC4492580; doi:10.1371/journal.pone.0131571)
Supplement: S3 Table — (DOCX) [file pone.0131571.s006.docx]

## S3 Table. Logistic regression analysis modeling the probability of being protected

| **Model Information** | **Immunologic variable** | Odds ratio (95%CI) | ***P-*value** |
| --- | --- | --- | --- |
| **ARR and RRR (N = 37)** | M1: CS Ab alone | 3.55 (1.30-9.72) | 0.014 |
|  | M2: CS Ab | 3.61 (1.25-10.45) | 0.018 |
|  | M2: polyfunctional T-cells | 0.79 (0.37-1.67) | 0.387 |
|  | M2: IFN-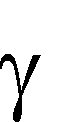ELISpotS | 1.09 (0.59-2.02) | 0.777 |
| Log-likelihood ratio | M1 – M2 |  | 0.821 |
| **ARR (N = 21)** | M1: CS Ab alone | 50.4 (0.97-26.31) | 0.055 |
|  | M2: CS Ab | 3.22 (0.44-23.69) | 0.252 |
|  | M2: polyfunctional T-cells | 27.77 (0.62->1000) | 0.086 |
|  | M2: IFN-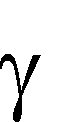ELISpotS | 0.02 (<0.01-1.41) | 0.071 |
| Log-likelihood ratio | M1-M2 |  | 0.034 |
| **RRR (N = 16)** | M1: CS Ab alone | 4.68 (0.65-33.71)) | 0.126 |
|  | M2: CS Ab | 21.87 (0.56-856.27) | 0.099 |
|  | M2: polyfunctional T-cells | 0.32 (0.09-1.16) | 0.083 |
|  | M2: IFN-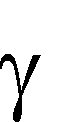ELISpotS | 0.77 (0.26-2.28) | 0.632 |
| Log-likelihood ratio | M1-M2 |  | 0.114 |

M1; Model 1 - anti-CS antibodies alone


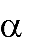

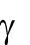
M2; Model 2 – anti-CS antibodies + polyfunctional T-cells + IFN-
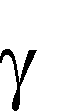
ELISpotS

Polyfunctional T-cells; CD4+ cells > 2 cytokine/activation markers (IL2, TNF- , IFN- , CD40L)

OR of being protected per 1 log-e increase in immune response
