# Supplementary material for: Ad35.CS.01 - RTS,S/AS01 Heterologous Prime Boost Vaccine Efficacy against Sporozoite Challenge in Healthy Malaria-Naïve Adults
Source: PLoS One. 2015 Jul 6;10(7):e0131571. doi: 10.1371/journal.pone.0131571 (PMC4492580; doi:10.1371/journal.pone.0131571)
Supplement: S1 Text — Supplementary tables and figure are available within in the Supplementary Information files. (DOCX) [file pone.0131571.s007.docx]

**S1 Text**

# Safety evaluation

Primary safety endpoints for this study were:


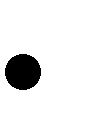
 Occurrence of each solicited adverse event within 7-day follow-up period (day of vaccination and 6 subsequent days) after each vaccination.


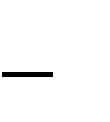
 Solicited local adverse event (any, grade 3).


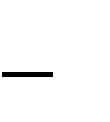
 Solicited general adverse event (any, grade 3, related).


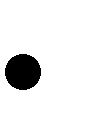
 Occurrence of unsolicited adverse events within 30 days (day of vaccination and 29 subsequent days) after each vaccination, according to the Medical Dictionary for Regulatory Activities (MedDRA) classification.


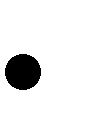
 Occurrence of unsolicited adverse events within 30 days (day of challenge and 29 subsequent days) after challenge, according to the Medical Dictionary for Regulatory Activities (MedDRA) classification.


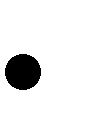
 Occurrence of serious adverse events (SAEs) within 30 days (day of vaccination and 29 subsequent days) after each vaccination, according to the Medical Dictionary for Regulatory Activities (MedDRA) classification.


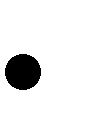
 Occurrence of SAEs during the whole study period (from dose 1 up to study conclusion), according to the Medical Dictionary for Regulatory Activities (MedDRA) classification.

Local injection site AEs included pain at the injection site and was graded as follows: 0 (absence of pain), 1 (painful on touch), 2 (painful when limb is moved), or 3 (spontaneously painful).

Redness and swelling were independently measured at the greatest surface diameter and were assigned the following grades: 0 (denoting 0 mm), 1 (> 0 - ≤ 50 mm), 2 (> 50 - ≤ 100 mm), or 3 (> 100 mm). General systemic symptoms included fever which was defined according to oral temperature assigned one of the following grades: 0 (38oC), 1 (≥38.0- < 38.5oC, 2 (≥ 38.5 - < 39.0oC), or 3 (≥39.0oC). Other general symptoms were graded as follows: 0 (normal), 1 (easily tolerated), 2 (interferes with normal activity), or 3 (prevents normal activity) and included

headache, fatigue, chills, and gastrointestinal symptoms (nausea, vomiting, diarrhea, abdominal pain).

# Immunomonitoring

### Serology

Antibodies levels against hepatitis B surface antigen (HBsAg) using an in-house validated ELISA assay. Results are expressed in mIU/ml and the cut-off for seroprotection is 10 mIU/ml. Ad35-specific neutralizing antibody titers were assessed by luciferase-based virus neutralization assays as described previously (ref: Sprangers MC, Lakhai W, Koudstaal W, Verhoeven M, Koel BF, Vogels R, et al. Quantifying adenovirus-neutralizing antibodies by luciferase transgene detection: addressing preexisting immunity to vaccine and gene therapy vectors. J Clin Microbiol. 2003;41(11):5046-52). Recently, the Adenovirus neutralization assay has been


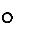
optimized and validated for human serum. In short, serum is heat inactivated and serially diluted by 2-fold (starting dilution is 1/16). Ad35.Luc (108 vp/ml) is added to each well at 500 virus particles per cell. A549 cells are added at 1x104 cells/ well and plates are incubated at 37 C/10% CO2 for 24 to 26 hours. After incubation, medium is discarded, PBS is added, and plates are stored frozen overnight. Plates are allowed to thaw at RT, Luciferase Steady-Lite substrate is added and the lysate is transferred into B&W isoplates. Luminescence counts are recorded on a 1450 MicroBeta Trilux. Titers were determined by validated software ANAM (Adenovirus neutralization Assay Macro). The limit of detection for the Ad35 neutralizing antibody assay was 16 IC90. Values below this threshold were set at 50% of the limit of detection.

### ELISpot assay

All experiments were performed on cryopreserved peripheral blood mononuclear cells (PBMC). Thawed and rested PBMC samples were stimulated with 59 CS peptides (2μg/ml/peptide), consisting of 15-mer peptides overlapping by 11 amino-acids, representing the CS protein. For mock stimulated wells, DMSO was used. For ELISpot assay, precoated human IFN-γ ELISpot plates (MabTech, Sweden) were blocked and PBMCs were added to the blocked plates at a concentration of 2 x 105 viable cells/well for peptide and mock stimulated wells. After overnight incubation the plates were washed and the detection antibody 7-B6-1-ALP (MabTech, Sweden) was added. After washing the spots were visualized with BCIP/NBT (MabTech, Sweden) and counted using a digital imager and automated spot counting (AID ELISpot reader, AutoImmun-Diagnostika). In each assay an internal control was included to determine if the assay run was valid. Results for ELISpot assay are presented as the number of spots per million

PBMC.

### Intracellular Cytokine Staining (ICS)

ICS assays on PBMC collected from subjects was performed according to methods previously published [13]. T cells were analyzed using 6-color panels on a FACSC (Becton Dickinson), and data analyzed for CD40L, IL-2, TNF-a, and IFN-y. Results were expressed as the number of CD4 or CD8+ T cells expressing > 2 of the immune markers per million cells.

# Statistical analysis

The interim analysis for futility was performed by a statistician external to the GSK-MVI- Crucell-WRAIR clinical teams. A blinded report at the subject level was provided for internal review by the GSK-MVI-Crucell-WRAIR clinical teams and an unblinded report generated for Safety Monitoring Committee review only. The co-primary study objectives were to compare the efficacy (occurrence of *P. falciparum* parasitemia, assessed by blood slide) and safety (reactogenicity and incidence of AEs/SAEs) of an Ad35.CS.01 prime, RTS,S/AS01 boost regimen to RTS,S/AS01 alone.

### Safety

The Intention-to-treat (ITT) cohort for safety included all subjects with at least one vaccine administration documented. The ATP population for efficacy included all subjects in the ITT population who received all vaccinations according to protocol procedures within the protocol specified intervals, met all eligibility criteria, did not use any medication or blood products forbidden by the protocol, did not report any underlying medical condition influencing immune responses, and underwent challenge.

All subjects who had received at least one dose of study vaccine were included in the safety analysis (Total vaccinated cohort). The percentage of doses followed by at least one solicited local/general AE was evaluated with exact 95% confidence intervals (CI). The percentage of subjects with at least one report of an unsolicited AE classified by the Medical Dictionary for Regulatory Activities (MedDRA) was evaluated with exact 95% CI. The same evaluations were performed for Grade 3 AEs and for AEs with a causal relationship to vaccination, as assessed by the investigator. Results from standardized neurological evaluations and urine analyses were

tabulated. Hematologic and biochemical laboratory values were assessed according to predefined grading scales.

### Humoral immunogenicity Analysis

Seropositivity rates for CS (cut-off ≥0.5 EU/mL) and neutralizing rates for Ad35 (cut-off 16 IC90) with exact 95% CI were assessed. Antibody titers were summarized by geometric mean titers (GMT) with 95% CI for both antigens. A statistical comparison was conducted to compare the geometric means of CS antibodies at post Dose 3 (Day 77; DOC) between the 2 groups ARR and RRR, and at post Dose 3 (Day 77; DOC) in the ARR group compared to post Dose 2 (Day

56) in the RRR group using a Student’s t-test (log transformed data).

***Cell mediated immune response (CMI)***

Descriptive statistics (geometric mean, mean, standard deviation, median, minimum, Q1, Q3 and maximum) were calculated per group and Box-plots generated. A statistical comparison was conducted to compare the geometric means of CS specific CD4+/CD8+ T-cells expressing at least 2 cytokines/activation markers (IFN-
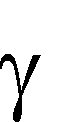
and/or IL-2 and/or TNF-α and/or CD40L) between the 2 groups ARR and RRR, and at post Dose 3 (Day 77; DOC) in the ARR group compared to post Dose 2 (Day 56) in the RRR group using a Student’s t-test (log transformed data). The same statistical comparisons were conducted for the geometric means of CS specific T-cells producing IFN-γ by ELISpot.

For intra-cellular staining (ICS) assays, the frequency of CD4+ or CD8+ T cells per million PBMC expressing a given cytokine was evaluated. For analyses of poly-functionality, data are presented as the frequency of CD4+ or CD8+ expressing each combination of cytokines.

### Modeling analysis

Modelling analysis was conducted to explore the relationship between the probability of being protected against malaria infection and several measured immune responses at the Day of Challenge (DoC). The analysis uses logistic regression to model these probabilities, and two models can be tested and compared: model 1 (M1) includes only the anti-CS antibody levels (thought to be the most important correlate of protection), while model 2 (M2) in addition also includes CD4 polypositives and IFN-γ (CS full length ELISpot) responses. Comparison of the

two models by means of a log-likelihood ratio test allows drawing a conclusion regarding the question whether CD4 polypositives and IFN-γ (CS full length ELISpot) responses explain anything over and beyond what is already explained by anti-CS antibody levels alone. A significant log-likelihood ratio test suggests that CD4 polypositives, and IFN-γ (CS full length ELISpot) responses add significantly to the explanation of protection probability over and above what is explained by anti-CS antibody levels alone, while a non-significant log-likelihood ratio test suggests that they don’t.

# IV. Eligibility Criteria

### Inclusion criteria for enrollment

All subjects must satisfy ALL the following criteria at study entry:


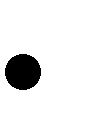
 Subjects who the investigator believes can and will comply with the requirements of the protocol (e.g. completion of the diary cards, return for follow-up visits).


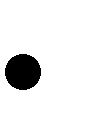
 A male or non-pregnant female 18 to 50 years of age (inclusive) at the time of first vaccination.


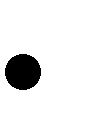
 Written informed consent obtained from the subject before screening procedures.


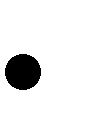
 Free of obvious health problems as established by medical history and clinical examination before entering into the study.


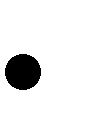
 Available to participate for the duration of the study (approximately 11 months per vaccinated subject or approximately 8 months per infectivity control).


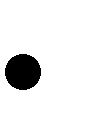
 Female subjects of non-childbearing potential may be enrolled in the study.


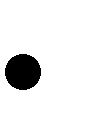
 Non-childbearing potential is defined as pre-menarche, current tubal ligation, hysterectomy, ovariectomy or post-menopause.


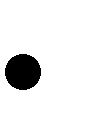
 Female subjects of childbearing potential may be enrolled in the study, if the subject:


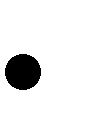
 has practiced adequate FDA-approved contraception for 30 days prior to vaccination, and


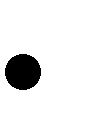
 has a negative pregnancy test on the day of vaccination, and


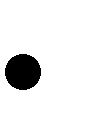
 has agreed to continue adequate FDA-approved contraception during the entire treatment period and for 2 months after completion of the vaccination series and/or malaria challenge.


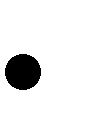
 Prior to entry into this study, subjects must score at least 80% correct on a short multiple- choice quiz that assesses their understanding of this study. If they do not score 80% on the initial quiz, the protocol information will be reviewed with them to ensure comprehension and they will have the opportunity to retest. Subjects who fail the Comprehension Assessment for the second time will not be enrolled.

### Exclusion criteria for enrollment

If ANY exclusion criterion applies, the subject must not be included in the study:


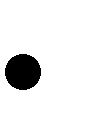
 Use of any investigational or non-registered product (drug or vaccine) within 30 days preceding the first dose of study vaccine, or planned use of any investigational or non- registered product (drug or vaccine) other than the study vaccines during the study period.


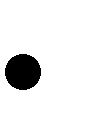
 Planned administration/ administration of a vaccine not foreseen by the study protocol within 7 days of the first dose of vaccines.


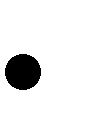
 Prior receipt of an investigational malaria or adenovirus vaccine.


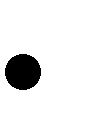
 Chronic use of antibiotics with antimalarial effects (e.g., tetracyclines for dermatologic patients, sulfa for recurrent urinary tract infections, etc.).


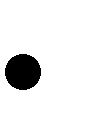
 History of malaria chemoprophylaxis within 60 days prior to vaccination.
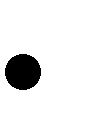
 Any history of malaria.


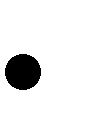
 Planned travel to malaria endemic areas during the study period.


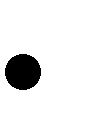
 History of allergic disease or reactions likely to be exacerbated by any component of the vaccine(s) including latex.


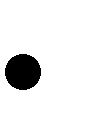
 History of allergic disease or reactions likely to be exacerbated by chloroquine.


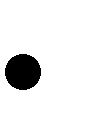
 History of psoriasis and porphyria, which may be exacerbated after chloroquine treatment.


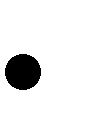
 Current use of medications known to cause drug reactions to chloroquine, such as antacids and kaolin.


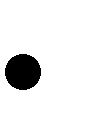
 Any history of anaphylaxis in reaction to any previous vaccination.
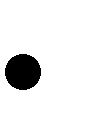
 History of severe reactions to mosquito bites.


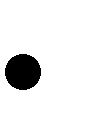
 Administration of immunoglobulins and/or any blood products within the three months preceding the first dose of study vaccine or planned administration during the study period.


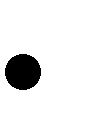
 Chronic administration (defined as more than 14 days) of immunosuppressants or other immune modifying drugs within six months prior to first vaccine dose. For corticosteroids, this will mean prednisone, or equivalent, greater than or equal to 20 mg /day. Inhaled and topical steroids are allowed.


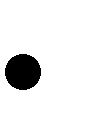
 Any confirmed or suspected immunosuppressive or immunodeficient condition, including immunodeficiency virus (HIV) infection.


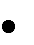

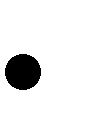
 Family history of congenital or hereditary immunodeficiency.

- History of splenectomy

Major congenital defects or serious chronic illness.


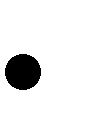
 History of any neurological disorders or seizures.


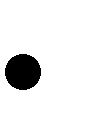
 Acute disease and/or fever at the time of enrollment.


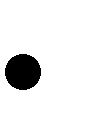
 Acute disease is defined as the presence of a moderate or severe illness with or without fever. Subjects with a minor illness (such as mild diarrhoea, mild upper respiratory infection) without fever may be enrolled at the discretion of the investigator.


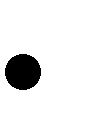
 Fever is defined as temperature ≥ 38.0°C (100.4°F) on oral, axillary or tympanic setting. The preferred route for recording temperature in this study will be oral.


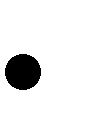
 Acute or chronic, clinically significant pulmonary, cardiovascular, hepatic or renal functional abnormality, as determined by physical examination or laboratory screening tests.


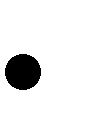
 Any abnormal baseline laboratory screening tests: ALT, AST, creatinine, hemoglobin, platelet count, total white blood cell count


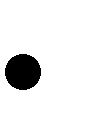
 Evidence of increased cardiovascular disease risk, “moderate” or “high”, according to the NHANES I criteria.


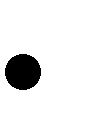
 An abnormal baseline screening electrocardiogram (EKG), defined as one showing pathologic Q waves and significant ST-T wave changes; left ventricular hypertrophy; any non-sinus rhythm excluding isolated premature atrial contractions; right or left bundle branch block; or advanced (secondary or tertiary) A-V heart block.


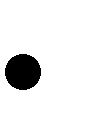
 Hepatomegaly, right upper quadrant abdominal pain or tenderness.
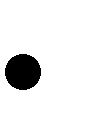
 Personal history of autoimmune disease.


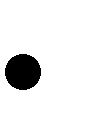
 Seropositive for hepatitis B surface antigen or Hepatitis C virus (antibodies to HCV).
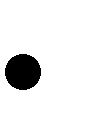
 Pregnant or lactating female.


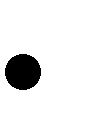
 Female who intends to become pregnant during the study or planning to discontinue contraceptive measures.


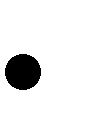
 Suspected or known current alcohol abuse.
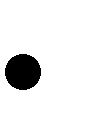
 Chronic or active intravenous drug use.


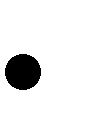
 History of blood donation within 56 days preceding enrolment.


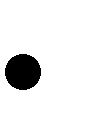
 Any other significant finding that in the opinion of the investigator would increase the risk of having an adverse outcome from participating in
